# Quinazolinones as Bioisosteres of Naphthoquinones: A Path to Potent *Hs*DHODH Inhibitors with Optimized Properties

**DOI:** 10.1021/acsmedchemlett.5c00237

**Published:** 2025-09-16

**Authors:** Bruna F. Godoi, Jéssica D. Bueno, Wemenes J. L. Silva, Aline D. da Purificação, Pedro I. P. Leite, Thiago dos Santos, Murillo Freitas, Daniel G. Silva, Tais C. Silva, Josué de Moraes, Caroline S. Freitas, Mayara Mattos, Thiago M. L. Souza, Bianca A. Martin, Renata F. V. Lopez, M. Cristina Nonato, Carolina H. Andrade, Flavio S. Emery

**Affiliations:** † Center for the Research and Advancement in Fragments and Molecular Targets (CRAFT), School of Pharmaceutical Sciences at Ribeirao Preto, 67782 University of São Paulo, Ribeirão Preto 14040-903, SP, Brazil; ‡ Laboratory of Heterocyclic and Medicinal Chemistry (QHeteM), Department of Pharmaceutical Sciences, School of Pharmaceutical Sciences at Ribeirao Preto, University of São Paulo, Ribeirão Preto 14040-903, SP, Brazil; § Laboratory for Molecular Modeling and Drug Design (LabMol), Faculty of Pharmacy, 488645 Universidade Federal de Goiás, Goiânia 74605-170, GO, Brazil; ∥ Protein Crystallography Laboratory, Department of Biomolecular Sciences, School of Pharmaceutical Sciences at Ribeirao Preto, University of São Paulo, Ribeirão Preto 14040-903, SP, Brazil; ⊥ Center for Excellence in Artificial Intelligence (CEIA), Institute of Informatics, 67824 Universidade Federal de Goiás, Goiânia 74605-170, GO, Brazil; # Research Center on Neglected Diseases, 92928 Guarulhos University, Guarulhos 07023-070, SP, Brazl; 7 Research Center on Neglected Diseases, Scientific and Technological Institute, Brazil University, São Paulo 08230-030, SP, Brazil; 8 Laboratory of Immunopharmacology, Oswaldo Cruz Institute (IOC), Oswaldo Cruz Foundation (Fiocruz), Rio de Janeiro 21040-360, RJ, Brazil; 9 National Institute for Science and Technology on Innovation in Diseases of Neglected Populations (INCT/IDPN), Center for Technological Development in Health (CDTS), Fiocruz, Rio de Janeiro 21040-900, RJ, Brazil; 10 Innovation Center in Nanostructured Systems and Topical Administration (NanoTop), School of Pharmaceutical Sciences at Ribeirao Preto, University of São Paulo, Ribeirão Preto 05508-060, SP, Brazil

**Keywords:** human dihydroorotate dehydrogenase, quinazol­inones, host-directed therapy, SBDD, SARS-CoV-2

## Abstract

Human dihydroorotate dehydrogenase (*Hs*DHODH) is a key enzyme in pyrimidine biosynthesis and a target for antiviral therapies against RNA viruses like SARS-CoV-2. Building on prior quinone-based inhibitors, we explored quinazol­inones as bioisosteric replacements to reduce cytotoxicity and off-target effects. Through structure-based design, we synthesized quinazol­inone derivatives aimed at maintaining critical binding interactions. First-generation compounds showed moderate *Hs*DHODH inhibition (up to 60% at 250 μM), with compound **10c** having an IC_50_ of 25 μM. Using computational modeling, we optimized second-generation derivatives, with **10e** showing the highest potency (IC_50_ = 0.59 ± 0.03 μM) and significant antiviral activity against SARS-CoV-2 (EC_50_ = 0.15 ± 0.03 μM). These compounds demonstrated improved selectivity compared to naphtho­quinone analogs, though challenges with aqueous solubility remain. These results highlight quinazol­inones as promising scaffolds for further development of anti-SARS-CoV-2 therapies targeting *Hs*DHODH.

## Graphical abstract

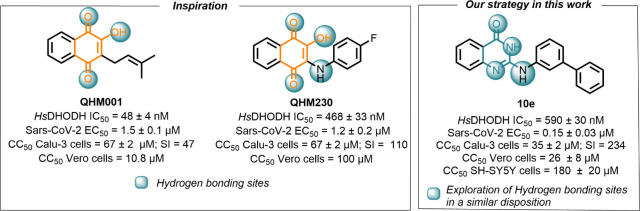

The *de novo* synthesis of pyrimidine nucleotides represents a conserved metabolic pathway across bacteria, protozoans, and animals, providing essential building blocks for DNA and RNA biosynthesis.^1^ Dihydroorotate dehydrogenase (DHODH) catalyzes the fourth and rate-limiting step in this pathway.^2,3^ Located on the outer surface of the inner mitochondrial membrane, human DHODH (*Hs*DHODH) catalyzes the oxidation of dihydroorotate to orotate through a flavin-dependent redox reaction (Figure 1A), which is subsequently utilized for the production of uridine monophosphate (UMP) and downstream pyrimidine nucleotides.^1,4^

**Figure 1:**
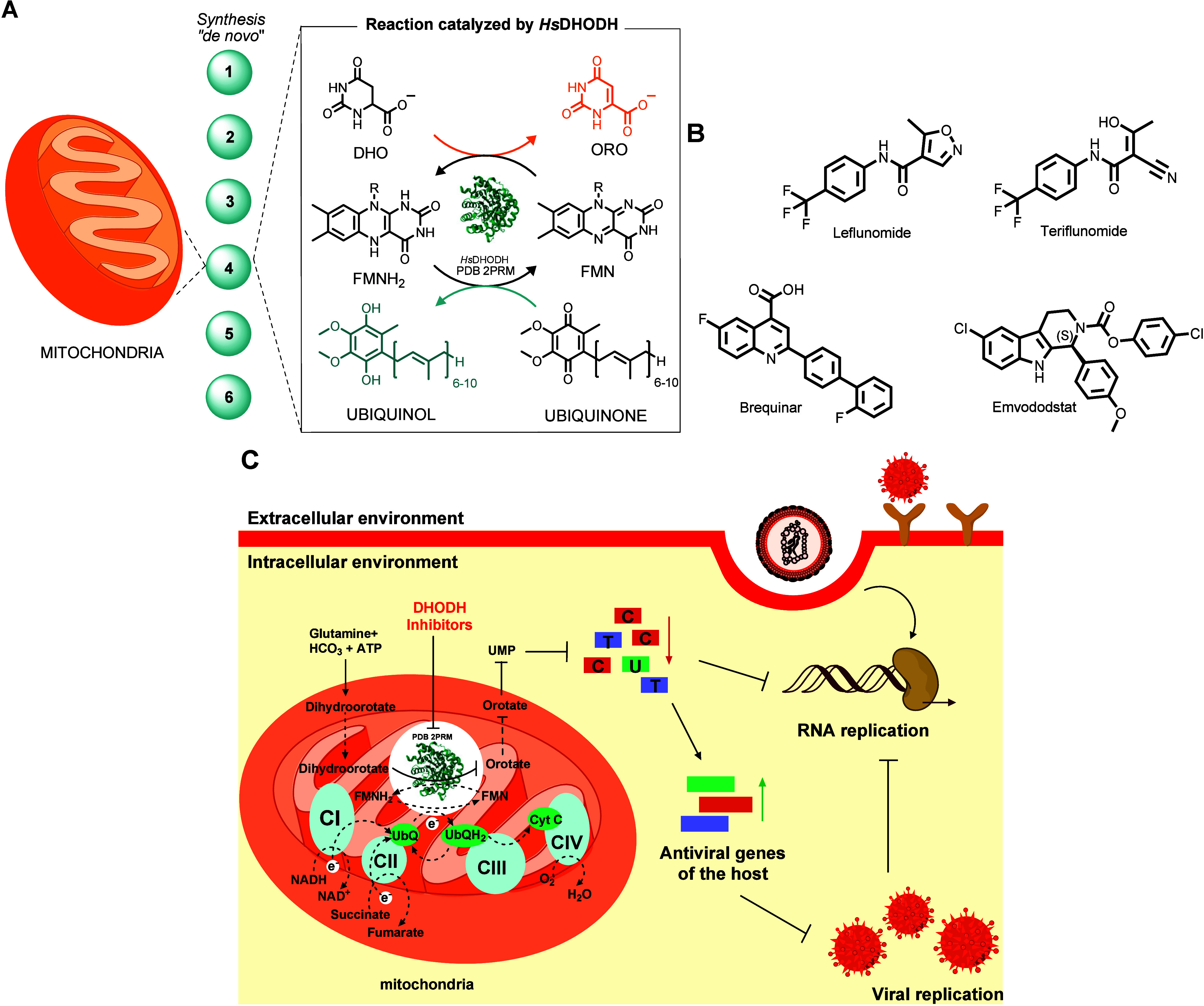
A) Ping-pong mechanism of the fourth step of pyrimidine biosynthesis, performed by the human DHODH. B) Known inhibitors of *Hs*DHODH. C) Strategy of broad-spectrum antivirals through *Hs*DHODH inhibition. The inhibition of DHODH reduces pyrimidine pools, which consequently inhibits RNA replication and activates antiviral genes of the host.

Although they are recognized as validated targets for drug discovery, a limited number of *Hs*DHODH inhibitors have reached clinical trials and advanced to the market. For instance, the prodrug leflunomide, metabolized to teriflunomide, is a potent *Hs*DHODH inhibitor that was approved for the treatment of rheumatoid arthritis (Figure 1B).^5–7^

Recent investigations have explored *Hs*DHODH as a target to discover drug candidates for COVID-19.^6,8,9^ This metabolic pathway has gained significant attention as a promising target for antiviral therapeutics, as viruses rely extensively on host pyrimidine biosynthesis to support their replication cycles. This host-directed approach offers potential advantages for developing broad-spectrum antivirals with reduced susceptibility to viral resistance mechanisms (Figure 1C).^10–12^ Brequinar, originally developed as an anticancer agent,^11^ exhibits highly specific and potent inhibitory activity against *Hs*DHODH and shows low micromolar anti-SARS-Cov-2 activity (Figure 1B).^13,14^

Emvododstat (PTC299) emerged during the COVID-19 pandemic as a promising drug candidate targeting *Hs*DHODH, demonstrating dual therapeutic mechanisms through direct inhibition of pyrimidine biosynthesis and suppression of pro-inflammatory cytokine production (Figure 1B).^15^ Beyond SARS-CoV-2, emvododstat exhibits broad-spectrum antiviral activity against other RNA viruses, including Ebola virus and Hepatitis C virus.^13^ Although Phase 2/3 clinical trials for hospitalized COVID-19 patients initially showed encouraging results, these studies were ultimately terminated before completion.^16^ The clinical trial failure of *Hs*DHODH inhibitor monotherapy for SARS-CoV-2 is not discouraging the attempts to find clinical candidates targeting this enzyme, as combination therapy with antivirals is a promising approach to fight infections with RNA viruses.^17,18^ These preliminary data nonetheless contributed valuable insights regarding the therapeutic potential of inhibiting *Hs*DHODH as a host-directed antiviral drug discovery approach.

In our previous studies, we identified a series of quinone-based compounds as potent inhibitors of *Hs*DHODH (as Lapachol and QHM230, Figure 2A), exhibiting nanomolar IC_50_ values and demonstrating in vitro activity against SARS-CoV-2 in the micromolar range.^19^ These findings highlight the therapeutic potential of *Hs*DHODH inhibition as a host-directed antiviral strategy. Molecular docking analyses revealed that these quinoidal inhibitors establish binding interactions similar to Brequinar at the enzyme’s ubiquinone binding site. Despite these promising results, the naphtho­quinone series displayed significant limitations including high cytotoxicity, poor aqueous solubility, and inherent scaffold liabilities related to promiscuous reactivity and metal chelation.^19–21^

**Figure 2:**
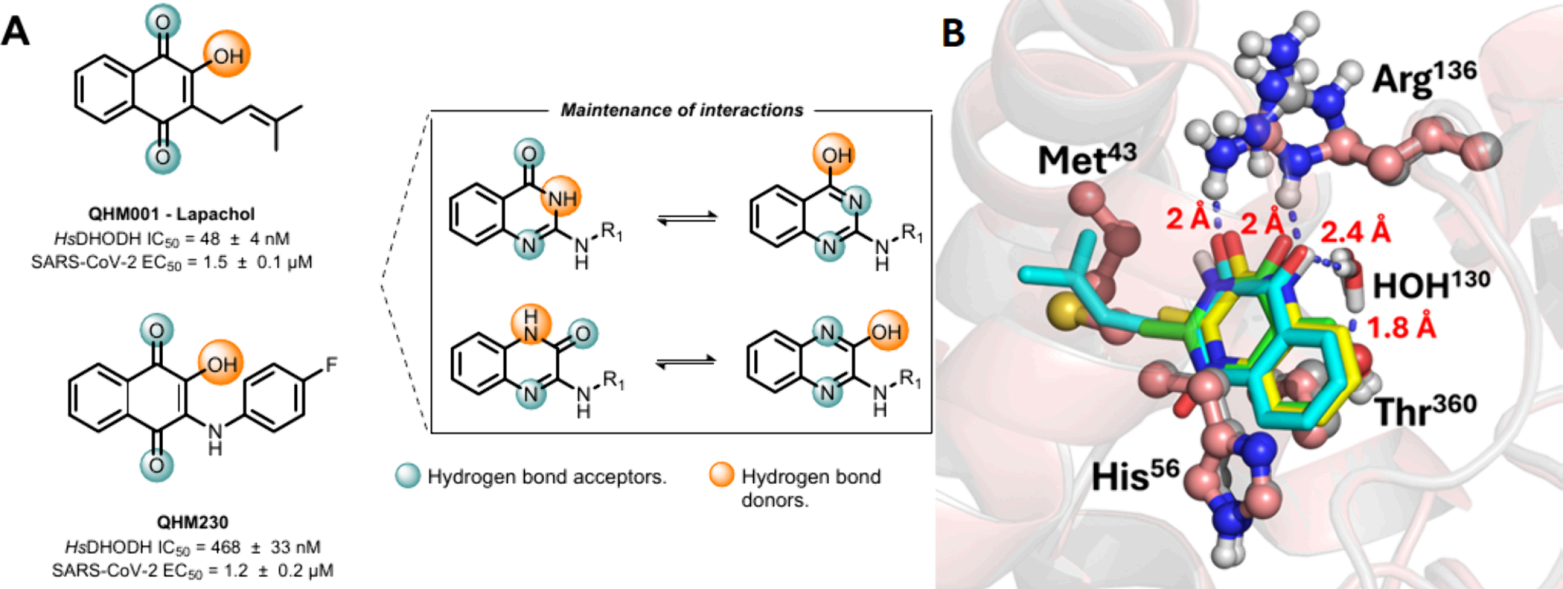
A) Structural conservation of hydrogen bond donor and acceptor groups in quinazol­inones and quinoxalinones, compared to the characteristic electronic configuration of napthoquinones described in prior studies (Lapachol and QHM230). B) Structural superposition of the *Hs*DHODH–Lapachol complex in its crystal conformation (PDB ID 9CCC), represented in cyan, with the resulting molecular docking models of the quinazol­inones (green) and quinoxalinones (yellow). Docking calculations were performed using the crystal structure PDB ID 6LP6 as a target. Hydrogen bonds between lapachol and residues in the *Hs*DHODH binding site are highlighted in blue dashed lines, and the interatomic distances are indicated.

To address these liabilities while maintaining potent inhibitory activity, we hypothesized that replacing the hydroxynaphtho­quinone core with isosteric quinazol­inone or quinoxalinone scaffolds could enhance drug-like properties while preserving critical binding interactions. In this work, through structure-based drug design (SBDD), we identified and validated quinazol­inones as promising bioisosteres of the naphtho­quinone core. Herein, we report the design, synthesis, molecular interactions, and biological evaluation of novel *Hs*DHODH inhibitors that demonstrate anti-SARS-CoV-2 activity with improved physicochemical and toxicological profiles. Our optimization strategy focused on enhancing both polar and hydrophobic interactions within the enzyme’s binding site. In the first screening, we selected matched pairs (such as fluorine and chlorine in the para position, the most potent compounds in the previous paper) in order to compare the inhibitory activity of quinones, quinazol­inones, and quinoxalinones. Moreover, since electron-donating groups were not assessed in the past paper, we also added *p*-methoxy and 2,3-dihydrobenzo­[*b*]­[1,4]­dioxin-6-yl groups to better understand the SAR.

We established two linear synthetic routes to obtain the designed quinoxalinones (**5a**–**d**) and quinazol­inones (**10a**–**c**) as depicted in Scheme 1. For quinoxalinones (Route A), we conjugated oxalic acid with 1,2-diaminobenzene under acidic conditions to afford 1,4-dihydroquinoxaline-2,3-dione (**2**) in 95% yield,^22^ which was further chlorinated via thionyl chloride to yield 2,3-dichloroquinoxaline (**3**).^23^ Selective hydrolysis of **3** using lithium hydroxide yielded 3-chloroquinoxalin-2­(1*H*)-one (**4**), which was then reacted with anilines to provide the desired set of 3-amino-substituted quinoxalinones with yields ranging from 48 to 85%.^23,24^ For 2-amino-substituted quinazol­inones (Route B), we reacted 2-aminobenzoic acid and urea according to Gong and co-workers’ procedure^25^ to produce quinazoline-2,4-diol (**7**, 71%) on a gram scale. After dichlorination with phosphoryl chloride, selective hydrolysis with sodium hydroxide, and nucleophilic substitution with the respective anilines, we obtained 2-amino-quinazolin-4-(3*H*)-ones with yields ranging from 61 to 82%.^25–28^

**Scheme 1:**
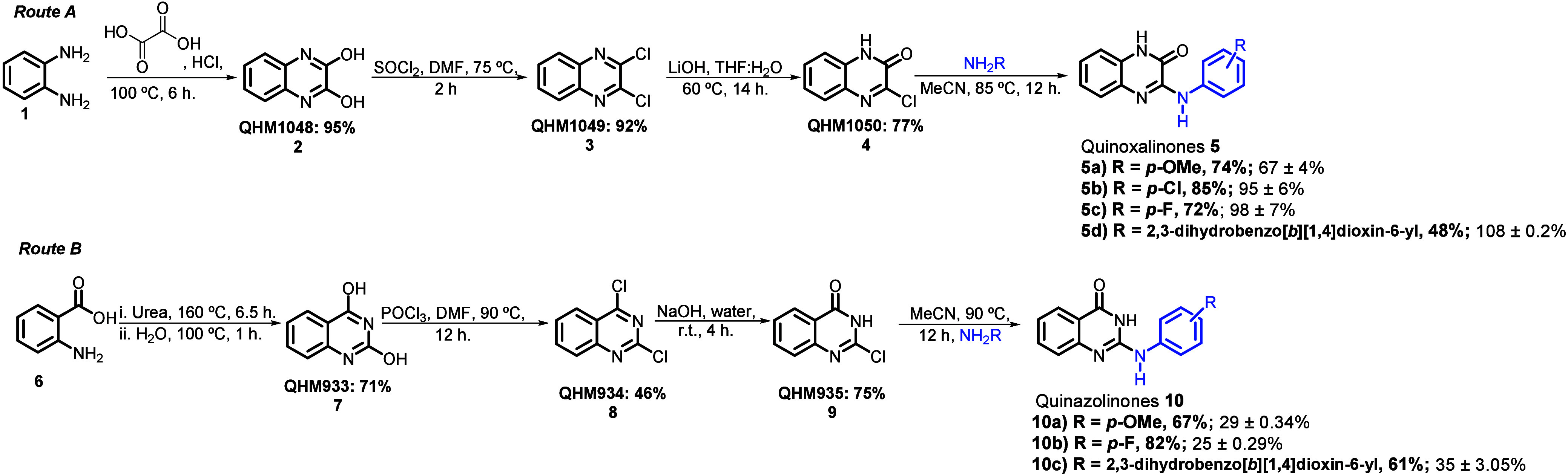
Synthetic Routes for the Synthesis of Quinoxalinones (Route A) and Quinazolinones (Route B)^a^

a The percentages highlighted in bold show isolated yields of compounds. The percentages in normal font show the enzymatic activity and standard deviation at 250 μM (100% activity = 0% inhibition).

Compounds **5a**–**d** and **10a**–**c** were evaluated for *Hs*DHODH inhibition in a single-dose assay at 250 μM, with enzymatic inhibition [±SD (%)] reported without preincubation. Notably, while quinazol­inones demonstrated significant inhibitory activity under the assay conditions, the quinoxalinone scaffold failed to inhibit the enzyme. The differences in inhibition profiles can be rationalized by computational analysis revealing suboptimal hydrogen bonding distances between quinoxalinones and the conserved water molecule (3.1 Å, Figure S3) compared to the more favorable interactions observed with Lapachol (2.4 Å) and quinazol­inones (2.3 Å, Figure S3). The altered positioning and orientation of the quinoxalinone carbonyl hydrogen bond acceptor, compared to the optimal geometry observed in lapachol (Figure 2B), appears to be a critical factor preventing effective enzyme inhibition, thus failing to mimic the essential interaction pattern established by our previous hits.^19^ The quinazol­inone derivatives (**10a**–**c**), however, exhibited promising inhibitory activity, with up to 75% inhibition of *Hs*DHODH in a single-dose assay. Compound **10c** demonstrated dose-dependent inhibition with an IC_50_ value of 25 ± 1 μM. Despite showing higher percent inhibition at the screening conditions, compounds **10a** and **10b** exhibited nonsigmoidal dose–response relationships that precluded reliable IC_50_ determination.

Figure 3A presents the dose–response curve for compound **10c**, calculated IC_50_ for *Hs*DHODH inhibition, and a 2D interaction analysis derived from a 50 ns molecular dynamics (MD) simulation, illustrating the relative frequency (%) of molecular interactions between compound **10c** and *Hs*DHODH. Throughout the MD simulation, **10c** maintained stable binding conformation, as evidenced by a root-mean-square deviation (RMSD) of less than 2 Å (Figure S1A). The analysis reveals a highly conserved hydrogen bond (98% occupancy) between the carbonyl group in **10c** and the side chain of Arg^136^ in *Hs*DHODH (Figure 3B). Additionally, transient hydrogen bonds (9% occupancy) were observed between the two N–H groups of **10c** and residues Gln^47^ and Thr^360^. Water-mediated interactions were also identified, bridging the N–H moieties of **10c** with residues Leu^46^ and Thr^360^. Notably, this hydrogen-bonding network between **10c** and residues Arg^136^, Thr^360^, and Gln^47^ corresponds to interactions previously reported for naphtho­quinoidal inhibitors,^19^ suggesting a conserved binding mechanism despite the scaffold modification.

**Figure 3:**
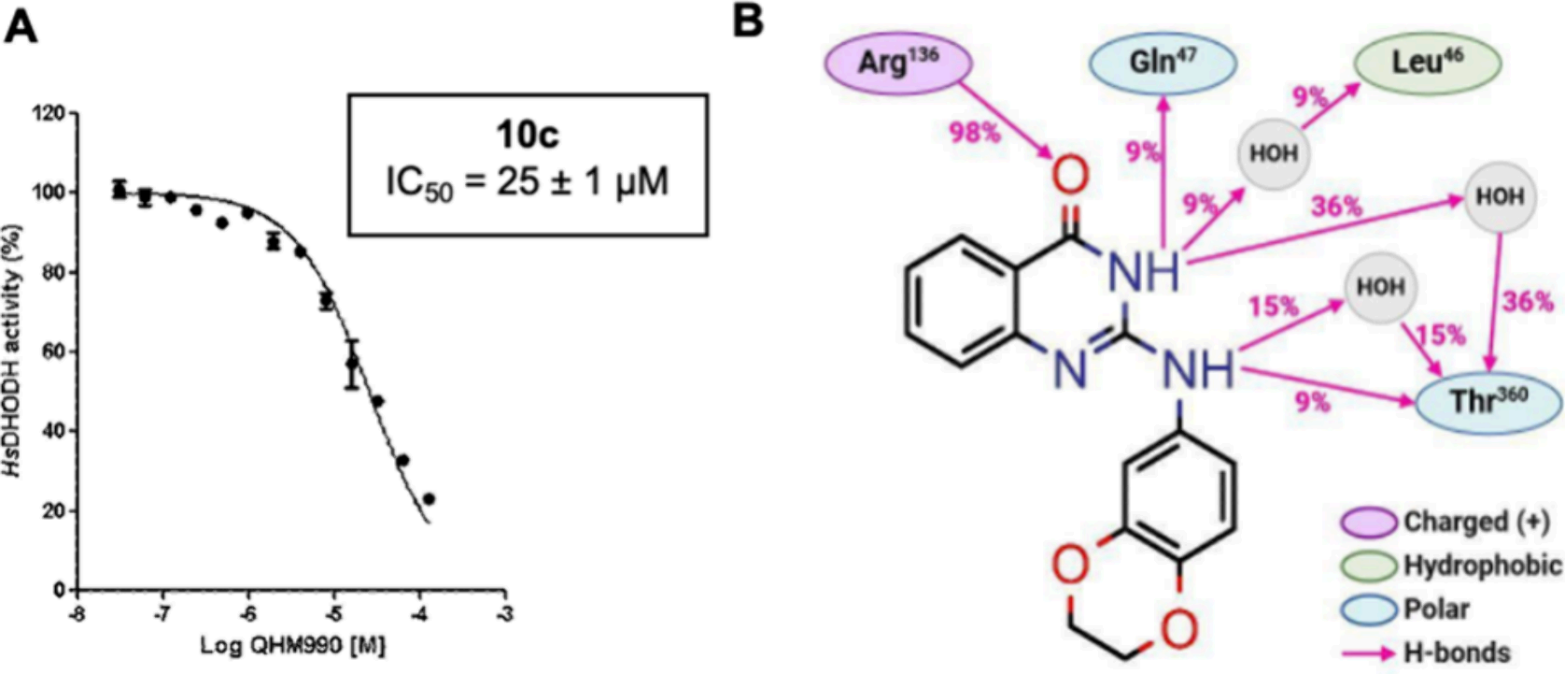
A) **10c** dose–response curve and IC_50_ for *Hs*DHODH inhibition. B) 2D MD analysis, highlighting the relative frequency (%) of molecular interactions between compound **10c** and the enzyme *Hs*DHODH.

Interestingly, during MD analysis, while the benzodihydrodioxine moiety exhibited no observable interactions within the *Hs*DHODH enzyme active site, the 2-amino­quinazol­inone core showed multiple interactions (Figure 3B). These observations support our hypothesis that the quinazol­inone scaffold effectively mimics the essential binding features of quinones, as demonstrated for lapachol (Figure 2B, PDB: 9CCC). This structural mimicry, coupled with no benzo­dihydro­dioxine-mediated interactions, guided the computational design of a second-generation quinazol­inone series to extend binding interactions toward unexplored hydrophobic regions of the enzyme binding pocket. This rational new design focused on enhancing *Hs*DHODH inhibitory potency through strategic optimization of key pharmaco­phoric elements while improving drug-like properties, particularly aqueous solubility, and preserving structural features critical for target engagement.

To accomplish this goal, we employed computational analysis of **10c**’s binding mode and developed compounds using two distinct optimization strategies (Figure 4):

**Figure 4:**
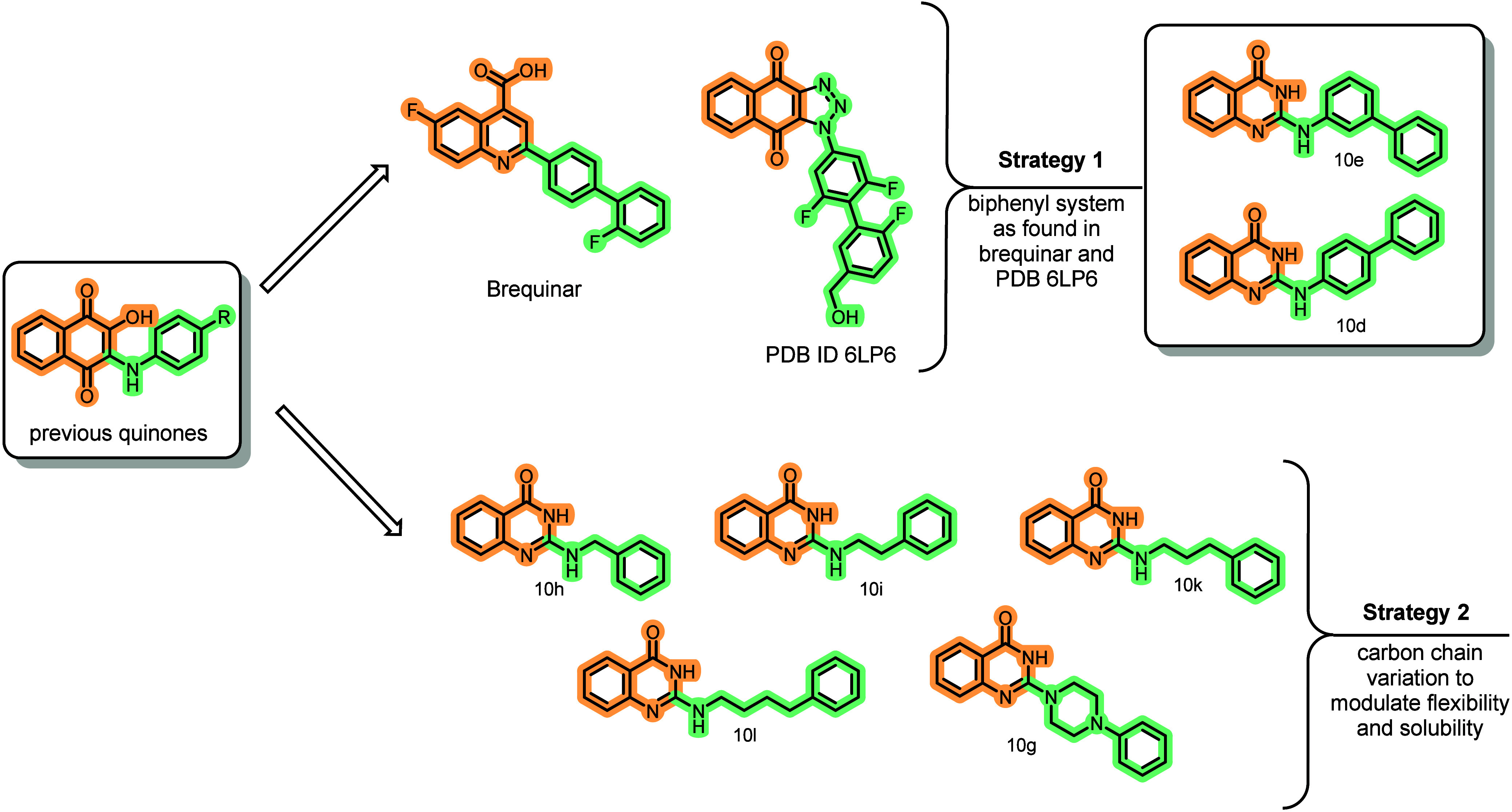
Design of the second generation of compounds. Quinazolinone ring as a bioisostere of naphtho­quinone core (highlighted in orange), and two different strategies planned to increase SAR understanding (substitutions highlighted in green).

The first approach was based on structural insights from the potent naphthotriazoledione inhibitor (IC_50_ = 1.2 nM), cocrystallized with *Hs*DHODH (PDB ID: 6LP6),^29^ which exhibits interaction patterns analogous to our previously characterized quinone-based inhibitors. The naphtho­triazole­dione scaffold incorporates a biphenyl ring system that effectively occupies subsite 1, a nomenclature adopted by Baumgartner et al. in 2006 ^30^ (Figure 4). Notably, this biphenyl motif is also present in Brequinar,^31^ further validating the significance of this pharmaco­phoric element in establishing favorable hydrophobic interactions within this region of the binding pocket (Strategy 1 in Figure 4).

The second approach evaluated the importance of an aliphatic linker between the amine −NH and the phenyl substituent to understand whether the added flexibility could improve potency by enabling different interaction patterns (Strategy 2 in Figure 4). Moreover, increasing the sp^3^ fraction (Fsp^3^) of designed compounds could modulate druglike properties, including solubility.^32^ Furthermore, the strategy also contemplates the ability of the series to occupy the hydrophobic subpocket of *Hs*DHODH.

The new designed quinazol­inones (**10d**–**l**) were obtained according to the same synthetic route, and the isolated yields varied from 28 to 75% (Table 1). Table 1 summarizes the enzymatic activity, IC_50_ of the most potent compounds, cytotoxicity in Vero and SH-SY5Y cells, and water solubility. Compound **10c**, the most potent inhibitor from our initial screening, demonstrates substantially lower potency compared to our second-generation compounds, validating our strategic scaffold optimization approach. The enzymatic inhibition data confirm the efficacy of our computational design strategy, with compound **10e** emerging as the most potent inhibitor of this series, exhibiting 96% inhibition of *Hs*DHODH and an IC_50_ of 0.59 ± 0.03 μM at 250 μM (Table 1). The 2D interaction analysis derived from a 50 ns MD simulation of compound **10e** reveals critical binding interactions (Figure 3C). Root mean square deviation (RMSD) analysis demonstrates that **10e** maintains a stable binding conformation throughout the simulation, with minimal positional fluctuations (<1.5 Å) (Figure S2C). In addition to preserving the key interactions with Arg^136^ and the water-mediated hydrogen bond with Thr^360^ observed in **10c**, compound **10e** establishes an additional hydrogen bond between the quinazol­inone carbonyl group and Gln^47^, which contributes significantly to binding stability. Furthermore, a novel water-mediated hydrogen bond forms between Ala^55^ and the N–H from the substituted aniline, providing additional anchoring points within the binding pocket.

**Table 1:**
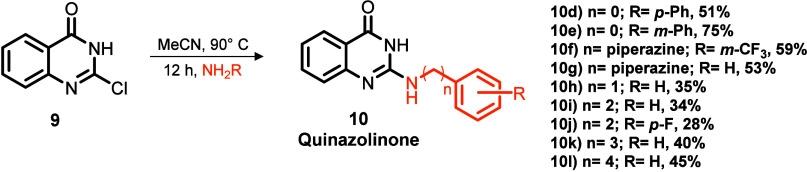
Experimental Results for Enzymatic Activity at 250 μM, *Hs*DHODH IC_50_, Cytotoxicity, and Solubility of the Second Generation of Compounds

			CC_50_ (μM)^a^		
Compound	Enzymatic Activity ± SD (%)	IC_50_ ± SD (μM)	Vero Cells	SH-SY5Y Cells	Water Solubility (μM)^b^
**10c**	15.1 ± 0.8	25 ± 1	390 ± 20	100 ± 19	26.0 ± 0.6
**10d**	49 ± 3	NA^c^	70 ± 10	130 ± 20	n.d.^d^
**10e**	4.2 ± 0.2	0.59 ± 0.03	26 ± 8	180 ± 20	n.d.^d^
**10f**	83 ± 7	NA^c^	161 ± 8	200 ± 20	n.d.^d^
**10g**	126 ± 1	NA^c^	150 ± 20	290 ± 20	n.d.^b^
**10h**	47 ± 3	NA^c^	50 ± 10	160 ± 20	35 ± 0.5
**10i**	2.5 ± 0.1	12 ± 1	110 ± 20	140 ± 20	57.0 ± 0.7
**10j**	8.2 ± 0.4	39 ± 2	40 ± 10	290 ± 30	30 ± 2
**10k**	18 ± 1	20 ± 1	40 ± 10	290 ± 20	30.0 ± 0.3
**10l**	34 ± 2	30 ± 2	60 ± 10	200 ± 20	n.d.^d^

a Values are expressed as a percentage of the control, and the 50% cytotoxic concentration (CC_50_) values were calculated based on three experiments using a 95% confidence interval.

b Water solubility determined using Procedure 2 (SI).

c NA stands for not applicable, out of the cutoff limit of 70% inhibition.

d n.d. stands for not determined, out of the detection limit.

More significantly, the enhanced potency of the *m*-substituted **10e** can be attributed to two key π–π stacking interactions formed between Phe^62^, Tyr^38^, and the terminal aromatic ring of the biphenyl substituent, interactions that are coherent with those observed in the cocrystal structure of the naphtho­triazole­dione inhibitor (PDB: 6LP6).^29^ The structural features of Brequinar and the tricyclic derivative^29^ further corroborate the critical importance of this biphenyl system in establishing optimal contacts with key amino acid residues. The computational prediction of diminished interactions with residues Ala^55^ and Tyr^38^ can rationalize the substantially reduced potency observed with the *p*-substituted analog.

To analyze the influence of aliphatic spacer groups between the −NH substitution of quinazol­inone ring and the phenyl on inhibitory potency, we systematically varied the carbon chain length in a homologous series (**10h**–**l**). Comparison of compound **10b** (*p*-fluor-aniline, lack of dose–response) with compound **10j** (*p*-fluor-phenethyl-amine), which incorporates an ethylene linker, revealed the latter as a moderate inhibitor, with an IC_50_ of 29 ± 2 μM for **10j**. Interestingly, these results did not align precisely with computational predictions which indicated that a larger spacer group would increase potency. Our experimental results suggest that an ethylene linker represents the optimal substitution pattern. Extension of this linker to three (**10k**) or four (**10l**) methylene units resulted in progressively diminished potency, despite MD analysis predicting a π-stacking interaction between compound **10l** and Phe^62^ (Figure S2G,H).

Compounds incorporating the piperazine moiety (**10f** and **10g**) exhibited favorable computational docking scores (Table S1) but demonstrated negligible inhibitory activity against *Hs*DHODH. This discrepancy suggests that the absence of a −N-H hydrogen bond donor at the C-2 position of the quinazol­inone scaffold is critical for activity. Furthermore, the nonaromatic heterocycle is more rigid that the linear ethylene linker. The loss of this key hydrogen bond donor eliminates essential interactions with active site residues, particularly Ala^55^ and Gln^47^. This structure–activity observation underscores the importance of maintaining specific hydrogen bonding capabilities within this region of the inhibitor scaffold for effective target engagement (Figure 5A).

**Figure 5:**
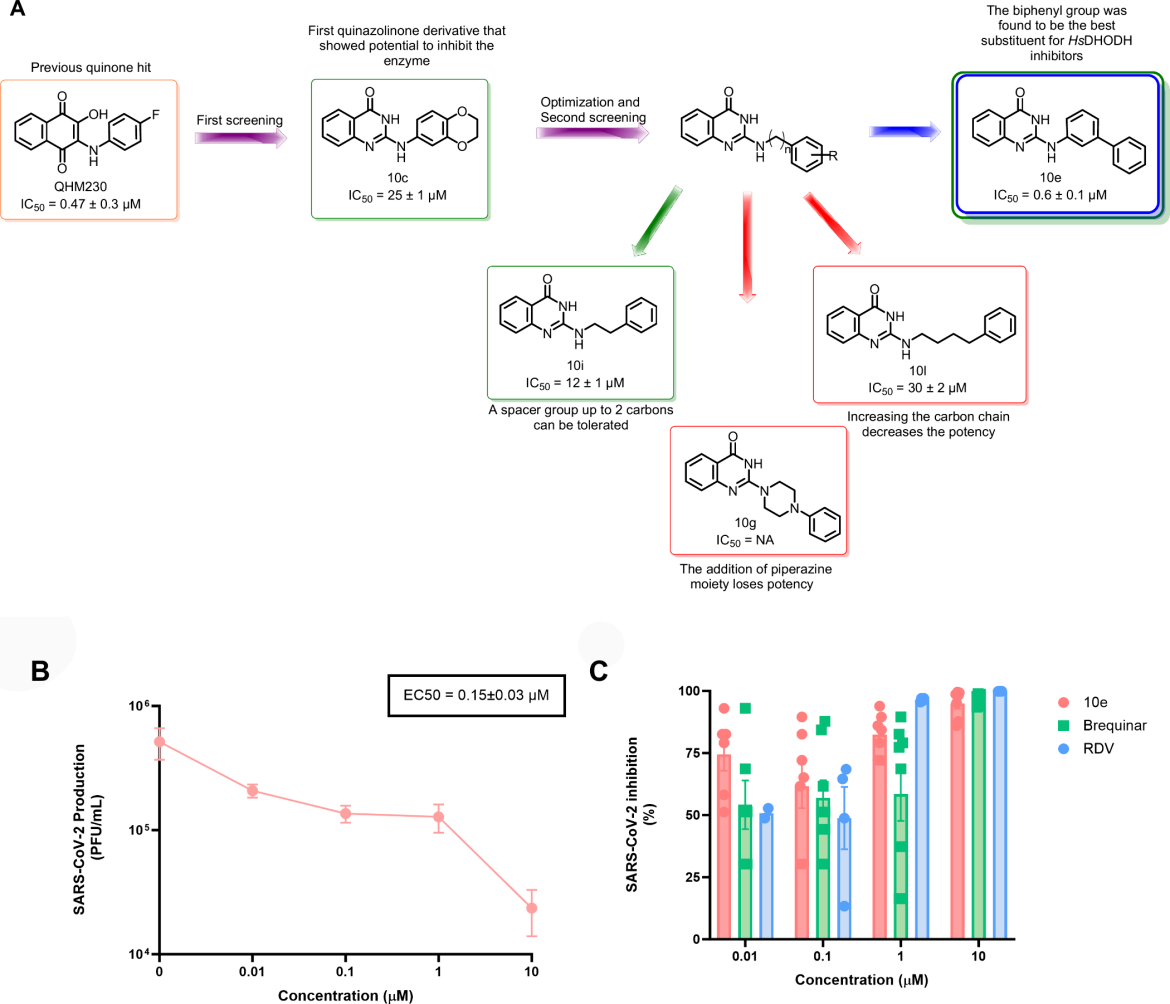
A) SAR diagram summarizing gains and losses of potency for *Hs*DHODH inhibitors. Purple arrows represent the screening. Red arrows represent a decrease in activity. Green arrows represent potency improvement. The blue arrow shows the hit of the work. B) Antiviral activity against SARS-CoV2 in Calu-3 cells of **10e** (orange dot, EC_50_ = 0.15 ± 0.03 μM) and C) percentage of SARS-CoV-2 inhibition, compared with the positive controls Brequinar (green square, EC_50_ = 0.8 ± 0.2 μM) and Remdesivir (blue dot, EC_50_ = 0.030 ± 0.002 μM).

In addition to the enhanced potency observed within our second-generation quinazol­inone series, a notable advantage emerged in their improved cytotoxicity profiles compared to our naphtho­quinone inhibitors (like Lapachol).^19^ Although compounds **10d** and **10e** exhibited moderate cytotoxicity in Vero cell lines, they demonstrated substantially reduced toxicity toward human SH-SY5Y neuroblastoma cells, confirming that the quinazol­inone scaffold provides optimized toxicological properties for *Hs*DHODH inhibition (Table 1). However, regarding aqueous solubility, the quinazol­inone series did not demonstrate significant improvement compared to the naphtho­quinone precursors. Consequently, enhancement of water solubility remains a critical parameter requiring further optimization in subsequent medicinal chemistry efforts.

Following the identification of **10e** as a potent inhibitor of *Hs*DHODH, we evaluated its potential as an antiviral candidate by assessing its cytotoxicity and antiviral activity against SARS-CoV-2 and determining the selectivity index (SI). As shown in Table 2, compound **10e** demonstrates potent anti-SARS-CoV-2 activity (EC_50_ = 0.15 ± 0.03 μM) in infected Calu-3 cells, with SI equal to 234, indicating a favorable safety profile. Notably, while **10e** exhibited comparable *Hs*DHODH inhibitory potency to our previously inhibitors,^19^ it demonstrated a 10-fold enhancement in antiviral activity compared to lapachol (EC_50_ = 1.5 ± 0.1 μM)—the most potent antiviral quinone in our studies—coupled with significantly reduced cytotoxicity.

**Table 2:** Antiviral Activity against SARS-CoV-2 in Calu-3 Cells of **10e** and **10i**

Compound	CC_50_ (μM) Calu-3	EC_50_ (μM)	SI
**10e**	36 ± 2	0.15 ± 0.03	234.7
Brequinar	–	0.8 ± 0.2	–
Remdesivir	–	0.030 ± 0.002	–

Figure 5B and C illustrates the comparative inhibition of SARS-CoV-2 production (PFU/mL) by compounds **10e**, Brequinar (EC_50_ = 0.8 ± 0.2 μM), and Remdesivir (EC_50_ = 0.03 ± 0.0015 μM), the latter two used as positive controls. The results highlight the exceptional antiviral potential of quinazol­inone derivative **10e**, demonstrating approximately 5-fold greater potency in suppressing viral replication compared to Brequinar under identical assay conditions and concentrations. This enhanced antiviral efficacy, combined with favorable selectivity indexes, positions this scaffold as promising candidates for further optimization as SARS-CoV-2 inhibitors targeting host pyrimidine biosynthesis.

In conclusion, this study demonstrates the successful design, synthesis, and anti-SARS-CoV-2 evaluation of novel quinazol­inone derivatives as potent inhibitors *Hs*DHODH. Employing bioisosteric replacement of the naphtho­quinone scaffold, we developed quinazol­inones that effectively mimic critical binding interactions within the ubiquinone binding site of the studied enzyme while addressing cytotoxicity and limitations of promiscuous properties inherent to quinone-based inhibitors. Through structure-based design and SAR analysis, we identified **10e** as the most promising compound, exhibiting high *Hs*DHODH inhibition (IC_50_ = 0.59 ± 0.03 μM) and antiviral activity against SARS-CoV-2 (EC_50_ = 0.15 ± 0.03 μM) with a favorable SI (234). MD simulations revealed key determinants of binding affinity, including conserved hydrogen bonding networks with Arg^136^, Thr^360^, and Gln^47^, as well as critical π–π stacking interactions with Tyr^38^ and Phe^62^ facilitated by the optimized biphenyl substituent. While our quinazol­inone derivatives demonstrate significantly improved potency and reduced cytotoxicity compared to their naphtho­quinone counterparts, aqueous solubility remains suboptimal and represents a primary focus for subsequent optimization efforts. Overall, these findings establish quinazol­inones as a promising core for *Hs*DHODH inhibition and advance the development of anti-SARS-CoV-2 candidates targeting host pyrimidine biosynthesis.

## Safety Statement

No unexpected or unusually high safety hazards were encountered.

## Supplementary Material



## Notes

Tables (docking scores, ligand efficiency, CC50, and solubility), figures (molecular dynamic simulation, docking models, and complex of ligand and *Hs*DHODH), and experimental description (synthetic, biological, and computational procedures and spectroscopic/spectrometric data) (PDF)

## ††

Universidade de São Paulo (USP) Faculdade de Ciências Farmacêuticas (FCF) Departamento de Tecnologia Bioquímico-Farmacêutica (FBT) Av. Prof. Lineu Prestes, 580 - Butantã, São Paulo - SP, 05508-000

## Abbreviations

Calu-3: human lung epithelium
CC_50_: cytotoxic concentration 50%
COVID-19: coronavirus disease 19
Da: Dalton
DCIP: 26-dichlorophenolindophenol
DCM: dichloromethane
DEMEM: Dulbecco’s Modified Eagle Medium
DHO: dihydroorotate
DMSO: dimethyl sulfoxide
DNA: deoxyribonucleic acid
EC_50_: half-maximal effective concentration
FBS: fetal bovine serum
FMN: flavin mononucleotide
GP: General Procedure
*Hs*DHODH: human dihydroorotate dehydrogenase
HEPES: (4-(2-hydroxyethyl)-1-piperazine­ethane­sulfonic acid)
HTS: high-throughput screening
IC_50_: half-maximal inhibitory concentration
IR: infrared
LC-MS/MS: liquid chromatography–tandem mass spectrometry
MD: molecular dynamics
*m*: *meta*
MTT: thiazolyl blue tetrazolium bromide
MOI: multiplicity of infection
NADPH: nicotinamide adenine dinucleotide phosphate hydrogen
Ni-NTA: nickel–nitrilo­triacetic acid
NMR: nuclear magnetic resonance
NPT: constant number of particles (N), system pressure (P), and temperature (T)
NVT: constant number of particles (N), system volume (V), and temperature (T)
*p*: *para*
PDB: Protein DataBank
PBS: phosphate-buffered saline
RDV: Remdesivir
RNA: ribonucleic acid
RMSD: root-mean-square deviation
SAR: structure–activity relationship
SARS-CoV-2: severe acute respiratory syndrome coronavirus 2
SD: standard deviation
SI: selectivity index
THF: tetrahydrofuran
TLC: thin-layer chromatography
UHPLC: ultra-high-performance liquid chromatography
UV: ultraviolet
Vero: African green monkey kidney
WHO: World Health Organization
