# Supplementary material for: Quinazolinones as Bioisosteres of Naphthoquinones: A Path to Potent HsDHODH Inhibitors with Optimized Properties
Source: ACS Med Chem Lett. 2025 Sep 16;17(1):99–108. doi: 10.1021/acsmedchemlett.5c00237 (PMC12794095; doi:10.1021/acsmedchemlett.5c00237)

Quinazolinones as Bioisosteres of Naphthoquinones: A Path to Potent HsDHODH Inhibitors with Optimized Properties

SUPPORTING INFORMATION

Bruna F Godoi[1,2], Jéssica D Bueno[1,2], Wemenes J L Silva[1,3,5], Aline D da Purificação[1,4], Pedro I P Leite[1,2], Thiago dos Santos[1,2], Murillo Freitas[1,3], Daniel G Silva[1,2], Tais C Silva[6], Josué de Moraes[6,7], Caroline S Freitas[8,9], Mayara Mattos[8,9], Thiago M L Souza[8,9], Bianca A Martin[10], Renata F V Lopez[10], M Cristina Nonato[1,4], Carolina H Andrade[1,3,5] and Flavio S Emery*[1,2]

1 Center for the Research and Advancement in Fragments and Molecular Targets (CRAFT), School of Pharmaceutical Sciences at Ribeirao Preto, University of São Paulo, Ribeirão Preto 14040-903, SP, Brazil

2 Laboratory of Heterocyclic and Medicinal Chemistry (QHeteM), Department of Pharmaceutical Sciences, School of Pharmaceutical Sciences at Ribeirao Preto, University of São Paulo, Ribeirão Preto 14040-903, SP, Brazil

3 Laboratory for Molecular Modeling and Drug Design (LabMol), Faculty of Pharmacy, Universidade Federal de Goiás, Goiânia 74605-170, GO, Brazil

4 Protein Crystallography Laboratory, Department of Biomolecular Sciences, School of Pharmaceutical Sciences at Ribeirao Preto, University of São Paulo, Ribeirão Preto 14040-903, SP, Brazil

5 Center for Excellence in Artificial Intelligence (CEIA), Institute of Informatics, Universidade Federal de Goiás, Goiânia 74605-170, GO, Brazil

6 Research Center on Neglected Diseases, Guarulhos University, Guarulhos, 07023-070, SP, Braz

7 Research Center on Neglected Diseases, Scientific and Technological Institute, Brazil University, São Paulo, 08230-030, SP, Brazil

8 Laboratory of Immunopharmacology, Oswaldo Cruz Institute (IOC), Oswaldo Cruz Foundation (Fiocruz), Rio de Janeiro, RJ, Brazil.

9 National Institute for Science and Technology on Innovation in Diseases of Neglected Populations (INCT/IDPN), Center for Technological Development in Health (CDTS), Fiocruz, Rio de Janeiro, RJ, Brazil.

10 Innovation Center in Nanostructured Systems and Topical Administration (NanoTop), School of Pharmaceutical Sciences at Ribeirao Preto, University of São Paulo, Ribeirão Preto 05508-060 SP, Brazil

* Corresponding Author:

Flavio S Emery: flavioemery@usp.br

Supplementary Table 1. Docking Scores and Ligand Efficiency of Second Generation of Quinazolinones

Title	Docking Score (kcal/mol)	Ligand Efficiency*
**10c**	-9.072	-0.412
10d	-11.972	-0.499
10e	-11.146	-0.464
10f	-11.017	-0.408
10g	-9.974	-0.435
10h	-7.905	-0.416
10i	-9.077	-0.454
10j	-9.695	-0.462
10k	-9.892	-0.471
10l	-10.301	-0.468

*[illegible] = [illegible], where N is the number of non-hydrogen atoms.

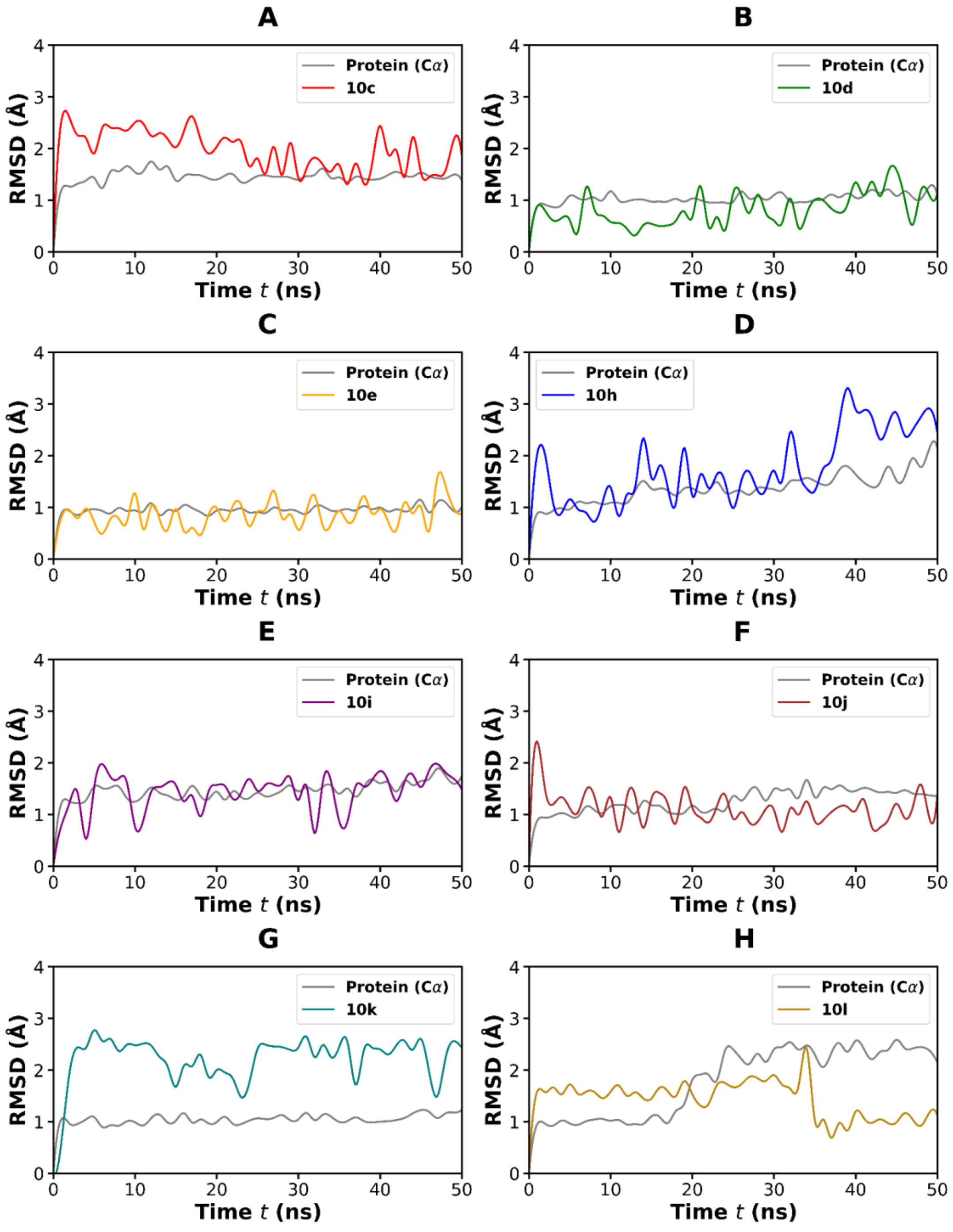

Supplementry Figure 1 - Root mean square deviation (RMSD) analysis of 50ns MD simulation trajectory for compounds 10c-10e, 10h-10l with HsDHODH. A) Compound 10c. B) Compound 10d. C) Compound 10e. D) Compound 10h. E) Compound 10i. F) Compound 10j. G) Compound 10k. H) Compound 10l. The gray line represents the RMSD calculated for the C-alpha atoms of HsDHODH, while the red, green, orange, blue, purple, brown, teal, and darkgoldenrod lines represent the RMSD calculated for the heavy atoms of the ligands relative to the last frame of the equilibration step.

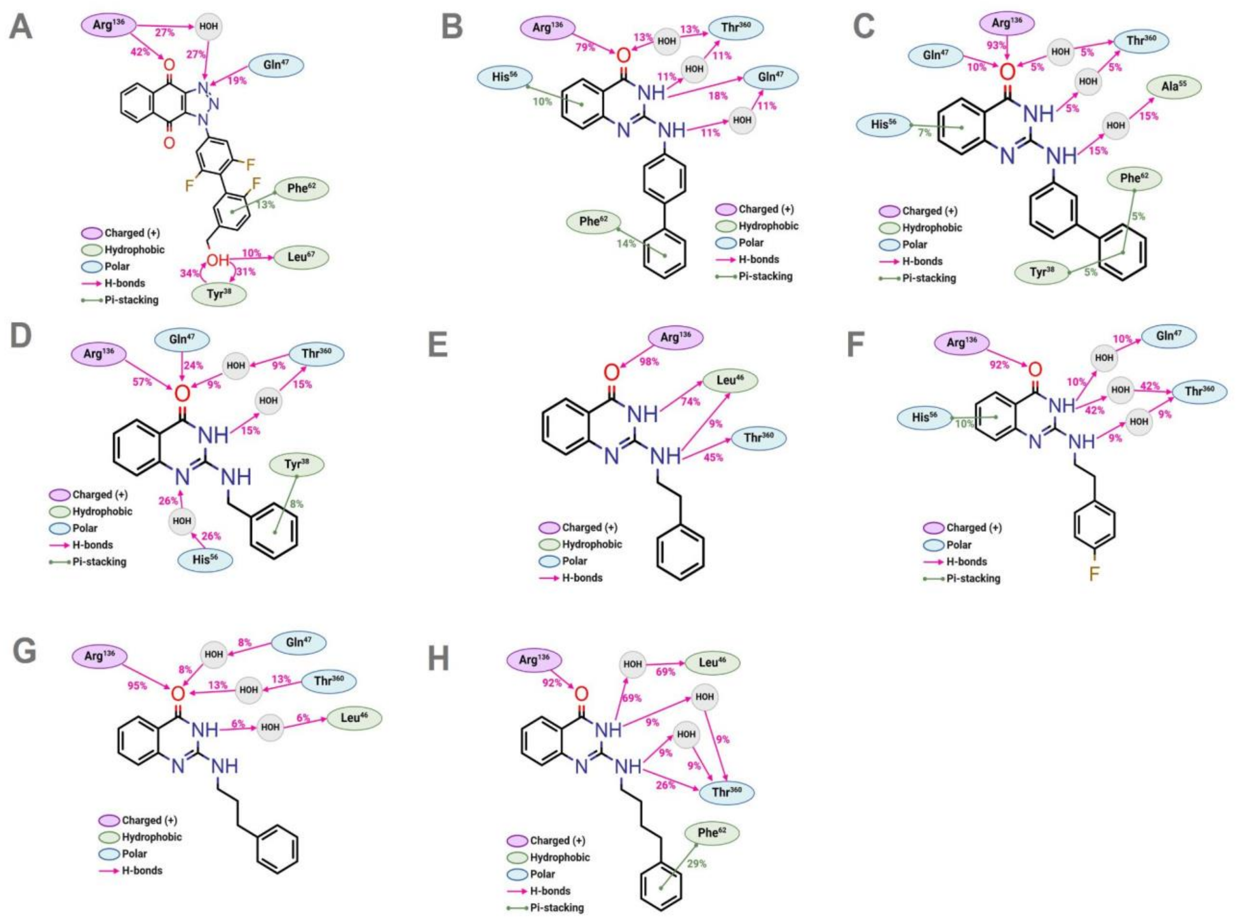

Supplementary Figure 2 - Key interactions observed for the selected compounds A) B6U, B)10d, C)10e, D)10h, E)10i, F)10j, G)10k and H)10l during 50 ns of MD simulation. The percentage values shown in pink and green indicate the fraction of time that specific residues interacted with the ligand. Only interactions with binding percentages exceeding 5% are shown. Pink lines represent hydrogen bonds, while green lines denote pi-pi stacking interactions. The charged, polar, and hydrophobic amino acids are depicted as light pink, blue, and green circles, respectively.

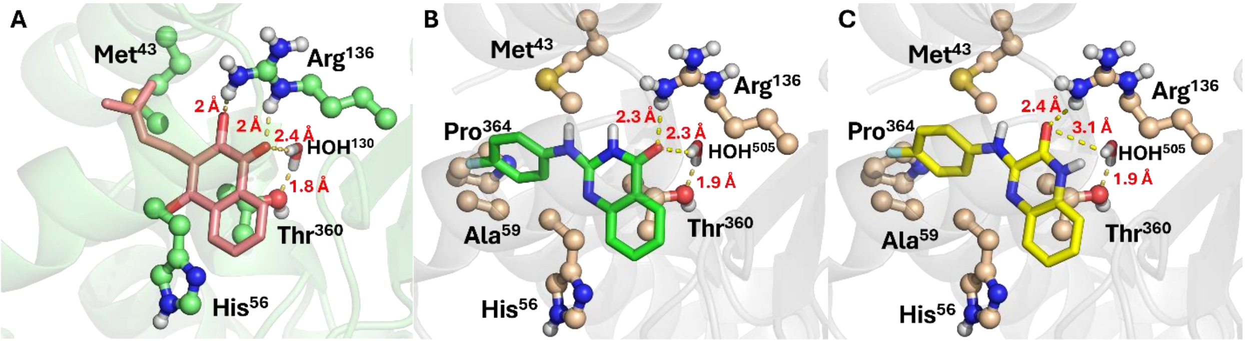

Supplementary Figure 3. A) Crystal structure of the Lapachol-enzyme complex (PDB ID: 9CCC) highlighting key molecular interactions. The hydroxyl group of the ligand remains deprotonated

at physiological pH 7.4, under the experimental conditions of the assay. B,C) Molecular docking models of the pair quinazolinone **10b** (B) and quinoxalinone **5c** (C) in the crystallographic structure of *Hs*DHODH (PDB ID: 6LP6), highlighting the hydrogen bonding interactions (dashed lines) between the amino acid residues of the enzyme and the atoms of the ligands, with interatomic distances indicated (in Å).

Supplementary Table 2. Cytotoxic concentration 50% (CC_{50}) of quinoxalinone and quinazolinone derivatives

Compounds	Vero cells CC_{50} (µM)	SH-SY5Y cells CC_{50} (µM)
Quinones		
Lapachol	10 ± 1	-
QHM230	100 ± 10	-
Quinoxalinones		
5a	411 ± 18	74 ± 11
5b	> 500	165 ± 13
5c	386 ± 15	201 ± 23
5d	> 500	188 ± 23
Quinazolinones		
10a	427 ± 24	165 ± 13
10b	392 ± 18	107 ± 19
10c	390 ± 20	100 ± 19
10d	70 ± 10	130 ± 20
10e	26 ± 8	180 ± 20
10f	161 ± 8	200± 20
10g	150 ± 20	290 ± 20
10h	50 ± 10	160 ± 20
10i	110 ± 20	140 ± 20
10j	40 ± 10	290 ± 30
10k	40 ± 10	290 ± 20
10l	60 ± 10	200 ± 20

Values were expressed as a percentage of the control, and the 50% cytotoxic concentration (CC_{50}) values were calculated based on three experiments using a 95% confidence interval.

<u>Experimental Section</u>

Computational Studies: MD e Docking

Protein and ligand preparation

The selected 3D X-ray crystal structure of the *Hs*DHODH complex with a ligand (PDB ID: 6LP6)[1] were prepared using the Protein Preparation Wizard[2] available in Schrödinger Suite (Schrödinger, L. Maestro Schrödinger 2021-4)[3] . The module filled missing atoms, adjusted side chains, and ensured the accuracy of atomic charges. Protonation and tautomeric states of amino acids were modified to match a pH of 7.4. Hydrogen bond sampling and adjustment of water molecule orientations were performed using PROPKA at pH 7.4. Structural water within 3 Å of the co-crystallized ligand was preserved, and a minimization process with the OPLS4 force field[4] was executed until an average root mean square deviation (RMSD) of 0.3 Å for the non-hydrogen atoms was achieved.

The compounds were prepared using the LigPrep (Schrödinger, L. Schrödinger Release 2021-4: LigPrep)[5] in Schrödinger Suite (Schrödinger, L. Maestro Schrödinger Release 2021-4)[3]. OPLS4 force field[4] was used in all preparation steps. Each compound was assigned to a protonation and ionization state at a pH of 7.4.

Molecular docking

Docking simulations were performed using the prepared compounds and protein structures, with receptor grids generated employing the OPLS4 force field[4]. The grid was centered precisely on the ubiquinone-binding site and the centroid Arg136, respectively, and the cubic grid had a side length of 20 Å. No constraints were used in the receptor grid. Glide[6] program was used to perform the XP (Extra Precision[7]) docking mode score in the Schrödinger Suite (Schrödinger, L. Maestro Schrödinger Release 2021-4)[3]. All other parameters were set to defaults for the Glide docking process. During the docking simulations, ligands were treated as flexible structures, while the protein remained rigid.

Molecular dynamics (MD) simulations

MD simulations were performed using the Desmond[8,9] for 50 ns. The TIP3P water model[10] was used to simulate water molecules in an orthorhombic box under periodic boundary conditions, positioned such that the walls were at a minimum 10 Å distance from any system atom. The counter ions of Na^+ and/or Cl^- were also added to neutralize the system and to mimic the physical salt concentration of 0.15 M. The particle-mesh Ewald method was used to calculate long-range electrostatic interactions. A cut-off radius of 9.0 Å was applied for short-range van der Waals and

Coulomb interactions. Each solvated system was minimized and equilibrated using Desmond's default protocol in Maestro, which includes 2 NVT and 2 NPT restrained short simulations. All equilibrated systems were subjected to an MD run with periodic boundary conditions in the NPT ensemble using an OPLS4 force field[4] for 5 ns. The temperature of 310 K and the pressure of 1 atm of the systems were maintained by the Nosè–Hoover chain thermostat[11] and Martyna–Tobiase–Klein barostat[11,12] methods, respectively. The simulation interaction analysis module was used to derive statistical data on the ligand-protein interactions during the MD simulations.

Biochemical assays

Inhibitory assays were conducted by indirectly measuring enzymatic activity, monitoring the reduction of 2,6-dichlorophenolindophenol (DCIP), according to a previously established protocol.[49] Readings were performed on a 96-well microplate reader, in a reaction buffer containing 60 µM DCIP, 50 mM Tris pH 8.15, 150 mM KCl, 0.1% Triton X-100, 500 µM DHO, 100 µM Coenzyme-Q0, and inhibitor at varying concentrations. Inhibitors were previously diluted in 100% DMSO. All assays were conducted in a reaction medium containing 2.5% DMSO. For single-dose assays, inhibitors were used at a concentration of 250 µM. For concentration-response assays, the initial inhibitor concentration was determined for each inhibitor based on the percentage inhibition in the single-dose assay. The dose-response curve was obtained from serial dilution of compounds in the reaction buffer. The reaction was performed in triplicate, initiated by adding 195 µL of reaction buffer containing or not containing inhibitors to 5 µL of HsDHODH protein solution for a final concentration of 20 nM enzyme. As a blank, 195 µL of reaction buffer containing or not containing inhibitors was added to 5 µL of protein purification buffer. The reaction was monitored at 610 nm every 3 s over 60 s for each concentration, and each compound was tested. As a control, 5 µL of enzyme was added to 195 µL of 2.5% DMSO reaction medium without inhibitors. Enzymatic velocity was calculated for each reaction, and the percentage of relative enzymatic

activity was determined relative to the control (DMSO). The IC50 value was determined from the graph of relative enzyme activity percentage versus the logarithm of the inhibitor concentration. The dose-response curve was fitted according to Equation 1 using GraphPad Prism 5 software.

$$Y = 100/(1+10(X\text{-}\log IC_{50}))$$ **Eq. (1)**

Cell assays: cytotoxicity in Vero and SH-SY5Y cells

Drugs and reagents.

Dulbecco's Modified Eagle Medium (DMEM), heat-inactivated fetal calf serum, penicillin G sodium salt, and streptomycin sulfate were obtained from Vitrocell (Campinas, SP, Brazil). Thiazolyl blue tetrazolium bromide (MTT), HEPES (4-(2-hydroxyethyl)-1-piperazineethanesulfonic acid) buffer, doxorubicin hydrochloride, and dimethyl sulfoxide (DMSO) were obtained from Sigma (St. Louis, MO, USA). In all experiments, compounds were solubilized in DMSO and used at a maximum concentration of 0.5% (v/v).

Cytotoxicity assay. The cytotoxicity of the compounds was evaluated using Vero cells (African green monkey kidney cells obtained from the American Type Culture Collection, ATCC; Manassas, VA, USA) and SH-SY5Y cells (human neuroblastoma obtained from Banco de Células do Rio de Janeiro, BCRJ, RJ, Brazil). The cells were cultured in DMEM medium supplemented with 10% fetal calf serum, penicillin (100 U/mL), and streptomycin (100 µg/mL) and 2 mM of L-glutamine at 37 °C in a 5% CO2 humidified incubator (Panasonic Healthcare Corporation, Tokyo, Japan) as previously described[13]. The DMEM contained 1 or 4 mg/mL glucose for Vero and SH-SY5Y cells, respectively. For toxicity determination, cells were seeded in 96-well plates (Corning, New York, NY, USA) using DMEM supplemented with 10% heat-inactivated fetal calf serum and 2 mM L-glutamine[14]. After 24 hours of cell adhesion at 37 °C and 5% CO2, the test compounds were added using a concentration range of 500-6.1 µM. Doxorubicin at 20 µM served as the positive control, while cells in DMEM medium with 0.5% DMSO were used as negative controls. Seventy-two hours post-incubation, MTT solution was added, and the plates were incubated for another three hours[15]. Subsequently, the plates were read using an Epoch Microplate

Spectrophotometer (BioTek Instruments, Winooski, VT, USA) at 595 nm. The experiments were conducted in triplicate and repeated three times. Values were expressed as a percentage of the control, and 50% cytotoxic concentration (CC50) values were calculated[16].

Antiviral in vitro assays

Cells, Viruses, and Reagents

African green monkey kidney (Vero, subtype E6) and human lung epithelium (Calu-3) cells were cultured in DMEM (Dulbecco's Modified Eagle Medium) high glucose medium. Culture media were supplemented with 10% fetal bovine serum (FBS), 100 U/mL penicillin and 100 µg/mL streptomycin. The cells were cultivated at 37 °C in a humidified atmosphere with 5% CO2. The SARS-CoV-2 was isolated on Vero E6 cells from a nasopharyngeal swab of a confirmed case from Rio de Janeiro, Brazil. The virus strain D614G was sequenced to confirm the virus identity, and its complete genome was publicly deposited (GenBank #MT710714; Institutional Review Broad approval, 30650420.4.1001.0008). All procedures related to virus culture were handled at biosafety level 3 (BSL3) multiuser facility, according to WHO guidelines.[60]

Antiviral *in vitro* assays

SARS-CoV-2 replication inhibition assays were performed in Calu-3 cells. To this end, cells were seeded in 96-well culture plates (2x104 cells per well), and, after 96 h, Calu-3 cells were infected with a MOI of 0.1 of SARS-CoV-2 for 1h at 37ºC. The inoculum was removed, and the cells were treated with the compounds at 10, 1, 0.1 and 0.01 µM diluted in fresh culture medium. Brequinar and Remdesivir (RDV) was used as positive controls for inhibition of viral replication. After 48 h, virus-containing supernatants were collected for quantification of viral replication using plaque formation assay.

All experiments were carried out at two independent times, and each data was analyzed from Prism GraphPad software 10 (Windows GraphPad Software, San Diego, California USA).

Triplicate experiments were performed for each data point, and the value was presented as mean ± standard deviation (SD).

Quantification of viral titers by plaque assay

Viral production in the supernatants of Calu-3 cultures used in in vitro assays was quantified through plaque formation assays. Vero E6 cells were infected with different dilutions of supernatants for 1h at 37ºC and, after this period, culture medium containing 2.4% carboxymethylcellulose was added. After 72h, cells were fixed with 3.7% formalin and stained with 0.04% crystal violet. Cell lysis plaques were counted, and viral titters were determined in plaque-forming units per mL (PFU/mL).[61]

Cell viability assay

Monolayers of Calu-3 cells (1.5 x 10^4 cells/well) in 96-well culture plates were incubated with Compound 10e at different concentrations (50, 100, 300, 500 and 700 μM) in DMEM High medium with 10% FBS for 48 h. Then, the dye resazurin was added (20 μl/ well) to the wells. After incubation for 3 h at 37 °C, the plates were read in a fluorimeter at 560 nm for excitation and 590 nm for emission. The 50 % cytotoxic concentration (CC_{50}) was calculated by performing a regression analysis on the dose–response curves generated from the data.

Solubility assays

Procedure 1: The procedure was the same described for our previous reported quinones[17].

Quartz cuvettes with 1 cm of the optical path were used for spectrophotometric quantification. Scans of known concentrations of the compounds were performed in DMSO/H2O 1:1, in the wavelength range (λ) between 220 and 600 nm, to determine the λ of maximum absorption (λmax)

of each of them in the UV/vis. The λmax was used to determine the solubility constant of the compounds from analytical calibration curves. The curves were constructed from known concentrations of the compounds in DMSO/H2O (1:1) in the concentration range from 0.1 to 50 µg/mL.

Each concentration absorbance (A) was plotted on the ordered axis, and the respective concentration was on the abscissa's axis. The least-squares linear regression method was used to fit the data points. The first-order equation $A = ax + b$ (where a is the slope and b is the linear coefficient, given by the line intersection point with the ordinate axis) was used to convert the sample's A in concentration (x). Linear ranges were calculated using the linear correlation coefficient (r) as the minimum acceptable criterion of $r = 0.99$.

Supplementary Table 3. Water:DMSO 1:1 solubility of quinoxalinone and quinazolinone derivatives using the spectrophotometric method (Procedure 1)

Compounds	Solubility (µM)
Quinoxalinones	
5a	59.8 ± 4.5
5b	26.1 ± 1.7
5c	n.d
5d	29.5 ± 2.5
Quinazolinones	
10a	11.0 ± 1.3
10b	31.5 ± 0.5
10c	26.2 ± 0.6
10d	n.d
10e	n.d
10f	n.d
10g	n.d
10h	35.3 ± 1.5
10i	57.0 ± 2.3
10j	29.7 ± 2.4
10k	30.2 ± 0.8
10l	n.d

nd. stands for not determined, out of the detection limit.

Procedure 2: The solubility of selected compounds was examined in two different aqueous buffers and are shown in Table 1 of the main text. First, to mimic the pH of blood, 1X phosphate buffered saline (PBS) was adjusted to a pH of 7.4. Second, to mimic the conditions of the stomach, 0.10 M HCl with a pH of 2.0 was used. In each of the two buffers, 1mg/1.5mL solutions of the test

compound were prepared. The solutions were incubated at 37°C overnight while agitating on a plate shaker (1500 rpm). After the incubation period, the solutions were centrifuged at 13500 rpm for 15 minutes at room temperature, and filtered using a syringe and PTFE 13mm membrane filter. Triplicate aliquots of 500 µL each solution's supernatant were isolated, diluted with 500 µL of methanol and analyzed using Liquid Chromatography Mass Spectrometry (LC-MS). Method development for the characterization of compounds was carried out with a LCMS system. The detector was set at 254 nm. The system was controlled, and data analyses were performed using the LC solutions software. The solvents were filtered through a 0.45 µm Merck-Millipore filter before use and degassed in an ultrasonic bath. Volumes of 50 µL (analytical) were injected. Quantification was carried out at 254 nm and the chromatographic run time varied according to each sample. The calibration curve was constructed using five different concentrations to determine the sample solubility, following Procedure 1 (as described above).

Method:

Equipment: UFLC Shimadzu Prominence equipment; UV detector: SPD-M20A; auto sampler: SIL-20A; pump: LC-20AD, LCMS-2020 Single Quadrupole LC/MS

solvent: A: water with 0.05 % (v/v) trifluoroacetic acid

B: CH_3CN

guard column: Shim-pack GIST C18 Guard Column

column: Shim-pack GIST C18 Capillary Column – 250 mm × 4.6 mm × 5 µm, Endcapped, pore size 10 nm or 100 Å; surface area 350 m²/g, pH range: 1.0 to 10.0, carbon loading: 10%.

temperature: 35 °C

gradient:

time [min]	solvent A [%]	solvent B [%]
6.0	95.0	5.0
15.0	0.0	100
19.0	95.0	5.0
20.0	95.0	5.0
40.0	90	10

flow rate:	0.5 mL/min
injection:	50.0 mL
detection:	wavelength: 254 nm
	stop time: 20 min
calculation:	integration: manual
	calculation method: area %

Chemistry section

General Remarks

Unless otherwise stated, all the solvents and reagents were obtained from commercial suppliers and used without prior purification. Chromatographic purification of the products was performed by flash column chromatography on silica gel (Sigma–Aldrich, particle size 0.040 – 0.063 nm).

Combiflash® and Biotage® were used for purification. Melting point values were collected in open capillaries using a Buchi, model B-545, to determine the melting point range (40-400 °C, 10.0 °C/min). Thin-layer chromatography (TLC) was carried out on silica plates (TLC Silica 60 F254 by Merck) and analyzed by UV light or by staining upon heating with vanillin or ninhydrin. NMR spectra were recorded on a Bruker Ultrashield 300-MHz NMR system (1H-NMR: 300 MHz, 13C-NMR: 75 MHz) or on a Bruker Ultrashield Avance 400-MHz NMR system (1H-NMR: 400 MHz, 13C-NMR: 101 MHz). Chemical shifts are referenced to residual solvent signals (DMSO-d6: 2.50 ppm and 3.33 for residual water in 1H-NMR, and 39.52 ppm in 13C-NMR, respectively), and reported in parts per million (ppm). Coupling constants (*J*) are reported in Hz, and multiplicities of NMR signals are abbreviated as follows: bs = broad singlet, s = singlet, d = doublet, dd= doublet of doublets, ddd = doublet of doublet of doublets, t = triplet, td = triplet of doublets, m = multiplet and combinations thereof, app = apparent. Melting points were determined in open capillary tubes by using a BÜCHI Labortechnik M-560 melting point meter. The record of high-resolution masses (HRMS) was made via a Bruker Daltonics micrOTOF QII/ESI-TOF in positive mode. SHIMADZU-IR-Tracer-100-Fourier Transform Infrared Spectrophotometer (solid sample + KBr) spectrometer fitted with an ATR accessory. Purity of compounds was calculated by qNMR (SI).

Synthetic procedures

Route A – Quinoxalinones

1.2. Synthesis of 1,4-dihydroquinoxaline-2,3-dione (7)

A 25 mL round-bottom flask was charged with oxalic acid (1.4 equiv., 1.0083 g, 11.2 mmol), 1,2-diaminobenzene (1.0 equiv., 0.8651 g, 8 mmol), and water (11 mL). Concentrated HCl min. 32 % (4.0 equiv., 32 mmol, 3.0 mL) was added dropwise, and the resulting solution was heated at 100 ˚C for 5 hours. Then, the reaction mixture was cooled to room temperature, and the resulting precipitate was isolated by filtration, washed with water, and dried (off-white solid, 2.4570 g, Yield = 95 %). **MP:** 152.6 – 153.2 ºC. **^{1}H-NMR (300 MHz, DMSO-d_6): δ [ppm]** = 11.92 (s, 2H), 7.14 – 7.06 (m, 4H). **^{13}C NMR (75 MHz, DMSO-d_6): δ [ppm]** = 155.2, 125.6, 123.0, 115.1. **HRMS (ESI-TOF) m/z**: $[M + H]^+$ calculated for $C_8H_7N_2O_2^+$: 163,0502; found 163,0500. Data in accordance with the literature.[1]

1.3. Synthesis of 2,3-dichloroquinoxaline (8)

A round-bottom flask (25 mL) was charged with 1,4-dihydroquinoxaline-2,3-dione (1.3612 g, 8.3948 mmol) and purged with N_2 two times. Then, DMF (1.0913 mmol, 13 mmol%, 84 µl) and $SOCl_2$ (9 equiv., 75.55 mmol, 5.5 mL) were consecutively added to the flask. The mixture was kept under stirring and N_2 atmosphere at 75 ˚C for 2 hours. After cooling, the reaction mixture was diluted with DCM (30 mL) and kept at 0 ˚C. Then, cold water was cautiously added (Warning: gas evolution may be observed with temperature increase) and the organic fraction separated. After two more extractions with DCM (2x30 mL), it was washed with brine (1x30 mL), dried over Na_2SO_4, and concentrated to obtain a white solid (1.6435 g, Yield = 92 %). **MP:** 151.2 – 152.0 ºC. **^{1}H-NMR (300 MHz, DMSO-*d*$_6$): δ [ppm]** = 8.12 – 8.07 (m, 2H), 7.98 – 7.92 (m, 2H). **^{13}C NMR (75 MHz, DMSO-*d*$_6$): δ [ppm] =** 144.7, 140.1, 131.8, 128.0. Data in accordance with the literature.[2]

1.4. Synthesis of 3-chloroquinoxalin-2(1*H*)-one (9)

A tube (10 mL) was charged with 2,3-dichloroquinoxaline (0.0995 g, 0.5 mmol), LiOH (1.0 mmol, 0.0240 g) and THF:H_2O (1.7 ml, 1:1, 0.85 ml of each one, 0.29 M). The tube was sealed, and the mixture was kept at 65 ˚C for 14 hours. The reaction was left to cool to room temperature, brine (30 mL) was added to the mixture, and the mixture was extracted thrice with THF (3x30 mL). The combined organic layers were further washed with brine and dried over Na_2SO_4. The desired product was obtained via column chromatography (Hexanes:AcOEt 4:6) as a white solid (0.0697 g, Yield = 77%). **Mp:** 276.7 – 278.1 ºC. **^{1}H-NMR (300 MHz, DMSO-*d*$_6$): δ [ppm] =** 12.87 (s, 1H), 7.72 (dd, J = 8.5, 1.4 Hz, 1H), 7.60 – 7.54 (m, 1H), 7.35 – 7.30 (m, 2H). **^{13}C NMR (75 MHz, DMSO-*d*$_6$): δ [ppm] =** 151.2, 149.0, 132.2, 130.9, 130.8, 127.9, 123.8, 115.6. **HRMS (ESI-TOF) m/z**: $[M + H]^+$ calculated for $C_8H_6ClN_2O^+$: 181,0163; found 181,0162. Data in accordance with the literature.[2]

1.5. General procedure for the synthesis of 3-amino-substituted quinoxalinones

To a round-bottom flask (25 mL) were added, 3-chloroquinoxalin-2(1*H*)-one (1.0 equiv., 0.66 mmol), the respective aniline (1.1 equiv.), and MeCN (5.5 mL, 0.12 M). The reaction mixture was heated from room temperature to 85 °C and stirred for 12 hours. Then, the solvent was removed and the dry residue was directly purified via column chromatography (Hexanes:AcOEt) to afford the desired product.[2]

3-((4-methoxyphenyl)amino)quinoxalin-2(1H)-one (5a): Employed aniline: 4-methoxyaniline (0.73 mmol, 0.0894 g). Purification via Hexanes:AcOEt (7:3). Beige solid (0.1377 g, Yield = 74%). **MP:** 242.1 – 242.4 ºC. **FTIR (cm^{-1}):** 3377, 1678, 1574, 1543, 1510, 1252, 823, 754. **^{1}H-NMR (300 MHz, DMSO-d_6): δ [ppm]** = 12.40 (s, 1H), 9.34 (s, 1H), 8.07 (app d, *J* = 9.1 Hz, 2H), 7.48 – 7.44 (m, 1H), 7.19 – 7.14 (m, 3H), 6.94 (app d, *J* = 9.1 Hz, 2H), 3.75 (s, 3H). **^{13}C NMR (75 MHz, DMSO-d_6): δ [ppm]** = 154.9, 151.6, 147.1, 132.8, 132.6, 128.4, 125.2, 124.4, 123.5, 121.3, 115.0, 113.7, 55.2. **HRMS (ESI-TOF) m/z**: $[M + H]^+$ calculated for $C_{15}H_{14}N_3O_2^+$: 268,1081; found: 268,1083.

3-((4-chlorophenyl)amino)quinoxalin-2(1H)-one (5b): Employed aniline: 4-chloroaniline (0.44 mmol, 0.0561 g). Purification via Hexanes:AcOEt (8:2) to 100% of AcOEt. White solid (0.0922 g, Yield = 85%). **MP:** 310.4 – 311.2 ºC. **^{1}H-NMR (300 MHz, DMSO-d_6): δ [ppm]** = 12.50 (s, 1H), 9.63 (s, 1H), 8.23 (app d, *J* = 9.0 Hz, 2H), 7.53 – 7.49 (m, 1H), 7.40 (app d, *J* = 9.0 Hz, 2H), 7.28 – 7.18 (m, 3H). **^{13}C NMR (75 MHz, DMSO-d_6): δ [ppm]** = 151.4, 147.2, 138.7, 132.1, 128.6, 128.4, 126.1, 125.5, 125.1, 123.5, 121.3, 115.1. **HRMS (ESI-TOF) m/z**: $[M + H]^+$ calculated for $C_{14}H_{11}ClN_3O^+$: 272,0585; found 272,0588. Data in accordance with the literature.[3]

3-((4-fluorophenyl)amino)quinoxalin-2(1H)-one (5c): Employed aniline: 4-Fluoroaniline (0.44 mmol, 0.0489 g, 42 µL). Purification via Hexanes:AcOEt (7:3). White solid (0.0755 g, Yield = 72 %). **MP:** 265.1 – 266.0 ºC. **^{1}H-NMR (300 MHz, DMSO-d_6): δ [ppm]** = 12.46 (s, 1H), 9.55 (s, 1H), 8.22 – 8.17 (m, 2H), 7.50 – 7.47 (m, 1H), 7.23 – 7.16 (m, 5H). **^{13}C NMR (75 MHz, DMSO-d_6): δ [ppm]** = 157.7(d, J_{C-F} = 239.5 Hz), 151.5, 147.2, 136.1 (d, J_{C-F} = 2.3 Hz), 132.2, 128.6, 125.4, 124.9, 123.5, 121.5 (d, J_{C-F} = 7.7 Hz), 115.1 (d, J_{C-F} = 22.1 Hz), 115.1. **HRMS (ESI-TOF) m/z**: $[M + H]^+$ calculated for $C_{14}H_{11}FN_3O^+$: 256,0881; found 256,0884. Data in accordance with the literature.[3]

3-((2,3-dihydrobenzo[b][1,4]dioxin-6-yl)amino)quinoxalin-2(1H)-one (5d): Employed aniline: 1,4-Benzodioxan-6-amine (0.44 mmol, 0.0665 g). Purification via Hexanes:AcOEt (7:3). Light yellow solid (0.0570 g, Yield = 48%). **MP:** 237.4 – 238.3 ºC. **FTIR (cm^{-1}):** 3373, 2980, 1672, 1602, 1574, 1562, 1503, 1208, 1068, 885, 756. **^{1}H-NMR (300 MHz, DMSO-d_6): δ [ppm] =** 12.41 (s, 1H), 9.31 (s, 1H), 7.86 (d, *J* = 2.6 Hz, 1H), 7.56 (dd, *J* = 8.8, 2.6 Hz, 1H), 7.47 – 7.44 (m, 1H), 7.20 – 7.17 (m, 3H), 6.83 (d, *J* = 8.8 Hz, 1H), 4.23 (q, *J* = 5.0 Hz, 4H). **^{13}C NMR (75 MHz, DMSO-d_6): δ [ppm] =** 152.0, 147.5, 143.2, 139.4, 133.8, 132.9, 128.8, 125.7, 125.0, 123.9, 117.0, 115.4, 113.7, 109.2, 64.6, 64.4. **HRMS (ESI-TOF) m/z**: $[M + H]^+$ calculated for $C_{16}H_{14}N_3O_3^+$: 296,1030; found 296,1030.

Route B - Quinazolinones

2.1. Synthesis of quinazoline-2,4-diol (QHM933 - 7):

2-aminobenzoic acid (20 mmol, 2.7428 g, 1.0 equiv.) and urea (7 equiv.,140 mmol, 8.4084 g) were added to a 25 mL round-bottom flask, and the temperature was adjusted from room temperature to 160 ˚C. After 6.5 h, it was allowed to cool to 100 ˚C, water (10 mL) was added, and the mixture was stirred for an additional 1 h. The reaction mixture was left to cool to room temperature and a precipitate was formed. The solid was filtered and washed with cold water to afford quinazoline-2,4-diol as a white solid (m= 2.3033 g, 14.20 mmol, Yield = 71 %). **MP:** 354.8 – 356.2 ºC. **^{1}H-NMR (300 MHz, DMSO-d_6)**: δ [ppm] = 11.29 (bs, 1H), 11.15 (bs, 1H), 7.88 (app dd, *J* = 8.5, 1.5 Hz, 1H), 7.63 (app td, *J* = 7.8, 1.5 Hz, 1H), 7.19 – 7.14 (m, 2H). **^{13}C NMR (75 MHz, DMSO-d_6):** δ [ppm] = 162.9, 150.3, 140.9, 135.0, 127.0, 122.4, 115.3, 114.4. **HRMS (ESI-TOF) m/z**: $[M + H]^+$ calculated for $C_8H_7N_2O_2^+$: 163,0501; found 163,0509. Data in accordance with the literature.[4]

2.2. Synthesis of 2,4-dichloroquinazoline (QHM934 - 8):

To a round-bottom flask (50 mL) with quinazoline-2,4-diol (2.3 g, 14.18 mmol), in room temperature, was added phosphorus oxychloride (15 mL) and N, N-dimethylformamide (0.1

equiv., 110uL). The reaction mixture was heated to 90°C and stirred for 12h. The reaction was monitored by TLC. After consumption of the starting material, the reaction mixture was evaporated under reduced pressure to afford a brown viscous oil, which was transferred to a beaker with ice cold water, and acetate was added slowly, and a precipitate was formed. The mixture was then filtered to obtain the desired product as a white solid (1.3 g, Yield = 46%). **MP:** 117.9 – 118.8 ºC. **^{1}H-NMR (300 MHz, DMSO-*d*$_6$): δ [ppm] =** 8.29 (d, *J* = 8.4 Hz, 1H), 8.16 (t, *J* = 7.7 Hz, 1H), 8.03 (d, *J* = 8.4 Hz, 1H), 7.90 (t, *J* = 7.7 Hz, 1H). Data in accordance with the literature.[4]

2.3. Synthesis of 2-chloroquinazolin-4(3*H*)-one (QHM935 - 9):

To a round-bottom flask (50 mL) with 2,4-dichloroquinazoline (0.1990 g, 1.0 mmol) was added a 2% solution of sodium hydroxide (30 mL). The reaction mixture was kept stirring at room temperature for 4 hours. Subsequently, it was filtered and acidified with glacial acetic acid (around 5mL or until you do not see any more precipitation of the solid product). The white precipitate was filtered and dried for 1 hour at 65 ºC to afford pure 2-chloroquinazolin-4(3H)-one. White solid (0.1350 g, Yield = 75%). **MP:** 212.1 ºC. **^{1}H-NMR (300 MHz, DMSO-*d*$_6$): δ =** 13.29 (bs, 1H), 8.10 (dd, *J* = 8.1, 1.0 Hz, 1H), 7.84 (app td, *J* = 7.3, 1.5 Hz, 1H), 7.62 (d, *J* = 8.1 Hz, 1H), 7.55 (app td, *J* = 7.3, 1.0 Hz, 1H). **^{13}C NMR (75 MHz, DMSO-*d*$_6$): δ =** 162.0, 147.8, 143.2, 135.1, 127.3, 126.6, 126.3, 120.8. **HRMS (ESI-TOF) m/z:** $[M + H]^+$ calculated for $C_8H_5ClN_2O^+$: 181,0163; found: 181,0163. Data in accordance with the literature.[5]

2.4. General method for the synthesis of 2-amino-substituted quinazolinones

In a round-bottom flask (25 mL), at room temperature, with 2-chloroquinazolin-4(3*H*)-one (1.0 equiv.) and the respective aniline (3.0 equiv.) was added MeCN (10 mL). The reaction mixture was heated to 90 ˚C and stirred for 12 hours or until total consumption of the starting material. Then, the reaction mixture was filtered and the residual solid was washed with acetonitrile and water to afford the desired products.[6]

2-((4-methoxyphenyl)amino)quinazolin-4(3H)-one (10a): Employed aniline: 4-methoxyaniline (102uL, 0,83 mmol). Purple solid obtained (50mg, 0,187mmol, Yield: 67,6%). **MP**: 251.6 ºC. **^{1}H-**

NMR (300 MHz, DMSO-d_6): δ = 11.23 (m, 1H), 8.93 (s, 1H), 7.95 (d, *J* = 7.9 Hz, 1H), 7.67 – 7.53 (m, 3H), 7.37 (d, *J* = 9.4, 8.1 Hz, 1H), 7.22 (t, *J* = 7.5 Hz, 1H), 6.95 (d, *J* = 10.5 Hz, 2H), 3.75 (s, 3H). **^{13}C NMR (101 MHz, DMSO) δ** 160.8, 156.7, 148.9, 144.7, 144.2, 140.0, 135.0, 126.3, 124.1, 123.9, 117.2, 114.5, 55.3 .**HRMS (ESI-TOF) m/z:** [M + H]+ calculated for $C_{15}H_{13}N_3O_2^+$: 268,1086; found: 268,1078. Data in accordance with the literature.[7]

2-((4-fluorophenyl)amino)quinazolin-4(3H)-one (10b): Employed aniline: 4-fluoroaniline (102uL, 0,83 mmol). White solid obtained (75 mg, 0,293 mmol, Yield: 81,6%). **MP**: 296.3 - 297.1 ºC. **^{1}H-NMR (300 MHz, DMSO-d_6): δ =**10.85 (s, 1H), 8.68 (s, 1H), 7.96 (app d, *J* = 7.8 Hz, 1H), 7.75 (app dd, *J* = 8.9, 4.9 Hz, 2H), 7.67 – 7.62 (m, 1H), 7.38 (d, *J* = 8.1 Hz, 1H), 7.25 – 7.16 (m, 3H). **^{13}C NMR (75 MHz, DMSO-d_6): δ =** 161.8, 157.8 (d, $J_{C\text{-}F}$ = 239.3 Hz), 150.0, 147.6, 135.4, 134.6, 126.0, 125.4, 123.2, 121.5 (d, $J_{C\text{-}F}$ = 7.9 Hz), 118.4, 115.5 (d, $J_{C\text{-}F}$ = 22.3 Hz). **HRMS (ESI-TOF) m/z**: [M + H]$^+$ calculated for $C_{14}H_{10}FN_3O^+$:256,0881; found: 256,0879. Data in accordance with the literature.[8]

2-((2,3-dihydrobenzo[b][1,4]dioxin-6-yl)amino)quinazolin-4(3H)-one (10c): Employed aniline: 2,3-dihydrobenzo[b][1,4]dioxin-6-amine (143uL, 1,16mmol). Light brown solid obtained (69mg, 0,23 mmol, Yield: 60,5%). **MP**: 256.1 - 262.0 ºC. **FTIR (cm^{-1}):** 3459, 1669, 1624, 1573, 1507, 1243, 1070, 888, 764. **^{1}H NMR (400 MHz, DMSO-d_6) δ** 10.75 (s, 1H), 8.47 (s, 1H), 7.96 (dd, *J* = 7.8, 1.6 Hz, 1H), 7.64 (ddd, *J* = 8.5, 7.2, 1.7 Hz, 1H), 7.47 (d, *J* = 2.5 Hz, 1H), 7.36 (d, *J* = 8.02 Hz, 1H), 7.21 (ddd, *J* = 8.0, 7.1, 1.1 Hz, 1H), 6.99 (dd, *J* = 8.7, 2.6 Hz, 1H), 6.84 (d, *J* = 8.7 Hz, 1H), 4.31 – 4.19 (m, 4H). **^{13}C NMR (75 MHz, DMSO) δ** 163.3, 150.8, 143.6, 141.3, 139.7, 134.9, 132.7, 127.4, 126.4, 123.4, 118.6, 117.4, 115.8, 109.6, 64.7, 64.4. **HRMS (ESI-TOF) m/z:** [M + H]$^+$ calculated for $C_{16}H_{13}N_3O_3^+$: 296,1030; found: 296,1022.

2-([1,1'-biphenyl]-4-ylamino)quinazolin-4(3H)-one (10d): Employed amine: 4-aminobiphenyl. White solid obtained after washing with acetonitrile, dichloromethane, ethyl acetate and water. Yield: 51%. MP: 310.5-313.8 ºC. **^{1}H NMR (400 MHz, DMSO-d6) δ** 9.95 (br s, 1H), 8.02 (dd, *J* = 7.9, 1.6 Hz, 1H), 7.80 – 7.69 (m, 7H), 7.53 – 7.46 (m, 3H), 7.39 – 7.31 (m, 2H). **^{13}C NMR (101 MHz, DMSO) δ** 161.1, 148.2, 139.5, 134.9, 129.0, 127.3, 127.2, 126.3, 126.2, 123.9, 121.6, 117.7. **HRMS (ESI-TOF) m/z:** [M + H]$^+$ calculated for $C_{20}H_{16}N_3O^+$: 314,1288; found 314,1282.

2-([1,1'-biphenyl]-3-ylamino)quinazolin-4(3H)-one (10e): Employed amine: 3-aminobiphenyl. White solid obtained after washing with acetonitrile, dichloromethane, ethyl acetate and water. Yield: 75%. MP: 275.6 – 278.0 °C**.** **^{1}H NMR (400 MHz, DMSO-d6) δ** 10.76 (s, 1H) 8.05 (dd, *J* = 7.9, 1.5 Hz, 1H), 7.97 (s, 1H), 7.78 (ddd, *J* = 8.5, 7.2, 1.6 Hz, 1H), 7.72 (app d, *J* = 7.2 Hz, 2H), 7.62 – 7.49 (m, 6H), 7.44 – 7.37 (m, 2H). **^{13}C NMR (101 MHz, DMSO) δ** 160.6, 149.3, 141.9, 141.7, 139.8, 136.8, 135.6, 130.7, 130.4, 129.5, 129.3, 128.5, 128.1, 127.0, 126.8, 124.9, 121.7, 117.4. **HRMS (ESI-TOF) m/z:** $[M + H]^+$ calculated for $C_{20}H_{16}N_3O^+$: 314,1288; found 314,1282.

2-(4-(3-(trifluoromethyl)phenyl)piperazin-1-yl)quinazolin-4(3H)-one (10f): Employed amine: 1-(3-(trifluoromethyl)phenyl)piperazine (219uL, 1,16mmol). White solid obtained (85mg, 0,23 mmol, Yield: 58,6%). **MP**: 252.0 - 254.8 °C. **FTIR (cm^{-1}):** 2951, 2860, 1682, 1615, 1450, 1324, 1121, 1001, 767, 694. **^{1}H NMR (300 MHz, DMSO-*d*$_6$) δ** 11.42 (s, 1H), 7.92 (dd, *J* = 7.9, 1.6 Hz, 1H), 7.60 (td, *J* = 7.7, 7.1, 1.6 Hz, 1H), 7.44 (t, *J* = 7.9 Hz, 1H), 7.36 – 7.21 (m, 3H), 7.17 (t, *J* = 7.4 Hz, 1H), 7.10 (d, *J* = 7.6 Hz, 1H), 3.79 (m, 4H), 3.35 (m, 4H). **^{13}C NMR (75 MHz, DMSO**) δ 151.5, 150.8, 141.3, 135.4, 134.7, 130.6 (d, J_{C-F} = 7.7 Hz), 130.2, 127.4, 126.7, 126.4, 122.9 (t, J_{C-F} = 11.4 Hz), 119.4, 117.7, 115.4, 111.7, 47.7, 44.9. **HRMS (ESI-TOF) m/z:** $[M + H]^+$ calculated for for $C_{19}H_{17}F_3N_4O^+$: 375,1427; found: 375,1418.

2-(4-phenylpiperazin-1-yl)quinazolin-4(3H)-one (10g): Employed amine: 1-phenylpiperazine (0,83 mmol). White solid obtained (50mg, 0,17 mmol, Yield: 53%). **MP**: 281.3 – 284.1 °C. **^{1}H NMR (300 MHz, DMSO-*d*$_6$) δ** 11.54 – 11.07 (m, 1H), 7.96 – 7.86 (m, 1H), 7.60 (ddd, *J* = 8.5, 7.0, 1.6 Hz, 1H), 7.32 (d, *J* = 8.2 Hz, 1H), 7.21 (s, 1H), 7.20 – 7.13 (m, 1H), 7.06 – 6.95 (m, 2H), 6.87 – 6.74 (m, 1H), 3.78 (dd, *J* = 6.5, 3.7 Hz, 4H), 3.22 (dd, *J* = 6.3, 3.7 Hz, 4H). **^{13}C NMR (75 MHz, DMSO) δ** 163.3, 151.3, 150.8, 141.3, 135.4, 134.7, 129.5, 127.4, 126.4, 122.9, 122.8, 119.7, 117.7, 116.2, 115.8, 114.8, 48.4, 45.1. **HRMS (ESI-TOF) m/z**: $[M + H]^+$ calculated for $C_{18}H_{19}N_4O^+$: 307,1557; found 307,1558.

2-(benzylamino)quinazolin-4(3H)-one (10h): Employed amine: benzylamine (1,16mmol). 1eq. of triethylamine was added to the reaction mixture. White solid obtained (39mg, 0,15 mmol, Yield: 35,03%). **MP**: 215.9 - 218.3 °C. **^{1}H NMR (400 MHz, DMSO-d6) δ** 10.88 (s, 1H), 7.88 (dd, *J* =

7.9, 1.6 Hz, 1H), 7.58 – 7.53 (m, 1H), 7.39 – 7.31 (m, 4H), 7.28 – 7.22 (m, 2H), 7.14 – 7.07 (m, 1H), 6.70 (s, 1H), 4.55 (d, *J* = 5.7 Hz, 2H). **13C NMR (101 MHz, DMSO) δ** 161.9, 150.5, 139.2, 134.2, 128.4, 127.4, 126.9, 125.9, 124.7, 122.3, 121.7, 117.5, 43.6. **HRMS (ESI-TOF) m/z:** [M + H]$^+$ calculated for $C_{15}H_{14}N_3O^+$: 252,1131; found: 252,1133.

2-(phenethylamino)-4(3H)-quinazolinone (10i): Employed amine: phenethylamine (1,16mmol). 1eq. of triethylamine was added to the reaction mixture. White solid obtained (34mg, 0,15 mmol, Yield: 34,2%). **MP**: 179.4 - 185.7 ºC. **^{1}H NMR (400 MHz, DMSO-d6) δ** 10.90 (s, 1H), 7.88 (dd, *J* = 7.8, 1.6 Hz, 1H), 7.57 (ddd, J = 8.5, 7.0, 1.7 Hz, 1H), 7.36 – 7.26 (m, 5H), 7.23 (td, *J* = 6.9, 1.7 Hz, 1H), 7.14 – 7.08 (m, 1H), 3.58 (q, *J* = 6.7 Hz, 2H). **13C NMR (101 MHz, DMSO) δ** 162.1, 150.6, 140.8, 139.3, 134.9, 134.1, 128.7, 128.4, 126.2, 125.9, 121.7, 117.3, 41.6, 34.8. **HRMS (ESI-TOF) m/z**: [M + H]+ calculated for $C_{16}H_{16}N_3O^+$: 266,1288; found: 266,1284.

2-((4-fluorfenetil)amino)quinazolin-4(3*H*)-ona (10j): Employed amine: (4-fluorophenyl)ethan-1-amine (153uL, 1,16mmol). White solid obtained (31mg, 0,1 mmol, Yield: 28,1%). White solid. **MP**: 235.9 - 239.0 ºC. **FTIR (cm^{-1}):** 3351, 1663, 1527, 1473, 1426, 1342, 1224, 836, 761. **^{1}H NMR (400 MHz, DMSO-*d*$_6$) δ** 10.89 (s, 1H), 7.88 (dd, *J* = 7.9, 1.6 Hz, 1H), 7.58 (ddd, *J* = 8.5, 7.1, 1.7 Hz, 1H), 7.35 – 7.26 (m, 3H), 7.18 – 7.08 (m, 3H), 6.21 (s, 1H), 3.57 (q, *J* = 6.7 Hz, 2H), 2.87 (t, *J* = 7.1 Hz, 2H). **^{13}C NMR (101 MHz, DMSO) δ** 162.1, 161.9, 159.7, 151.0, 150.4, 135.4, 134.1, 130.5, 130.5, 125.8, 124.7, 121.6, 117.4, 115.1, 114.9, 41.6, 33.9. **HRMS (ESI-TOF) m/z**: [M + H]$^+$ calculated for $C_{16}H_{14}FN_3O^+$: 284,1194; found: 284,1193.

2-(3-phenylpropylamino)-4(3H)-quinazolinone (10k): Employed amine: phenylpropylamine (1,16mmol). 1eq. of triethylamine was added to the reaction mixture. White solid obtained (50mg, 0,18 mmol, Yield: 40,08%). **MP**: 167.4 - 169.9 ºC. **^{1}H NMR (400 MHz, DMSO-d6) δ** 11.14 (bs, 1H), 7.89 (dd, *J* = 7.9, 1.6 Hz, 1H), 7.58 (ddd, *J* = 8.5, 7.1, 1.7 Hz, 1H), 7.33 – 7.10 (m, 7H), 6.64 (s, 1H), 3.36 (m, 2H), 2.71 – 2.62 (m, 2H), 1.87 (tt, *J* = 7.6, 6.4 Hz, 2H). **13C NMR (101 MHz, DMSO) δ** 162.1, 150.7, 141.5, 134.3, 128.3, 126.0, 125.8, 122.0, 117.1, 115.3, 114.3, 39.8 (overlap with solvent signal), 32.3, 30.5. **HRMS (ESI-TOF) m/z**: [M + H]$^+$ calculated for $C_{17}H_{18}N_3O^+$: 280,1444; found: 280,1442.

2-(4-phenylbutylamino)-4(3H)-quinazolinone (10l): Employed amine: phenylbutylamine (1,16mmol). 1eq. of triethylamine was added to the reaction mixture. White solid obtained (39mg, 0,15 mmol, Yield: 44,63%). **MP**: 172.5 - 175.0 ºC. **FTIR (cm^{-1}):** 3423, 3027, 2860, 1691, 1635, 1611, 1571, 1476, 1290, 868, 762, 698. **^1H NMR (400 MHz, DMSO-d6) δ** 10.81 (s, 1H), 7.88 (dd, J = 7.9, 1.6 Hz, 1H), 7.56 (ddd, J = 8.5, 7.0, 1.6 Hz, 1H), 7.30 – 7.14 (m, 7H), 7.09 (t, J = 7.5 Hz, 1H), 6.26 (s, 1H), 3.34 (m, 2H), 2.62 (t, *J* = 7.4 Hz, 2H), 1.70 – 1.48 (m, 4H). **13C NMR (101 MHz, DMSO) δ** 161.9, 151.1, 150.5, 142.1, 134.2, 128.3, 128.2, 125.9, 125.7, 124.5, 121.5, 117.3, 39.9 (overlap with solvent signal), 34.8, 28.5, 28.3. **HRMS (ESI-TOF) m/z:** $[M + H]^+$ calculated for $C_{18}H_{20}N_3O^+$: 294,1601; found: 294,1602.

REFERENCES

1. Zuo, Z. *et al.* Erratum: Bifunctional Naphtho[2,3- d][1,2,3]triazole-4,9-dione Compounds Exhibit Antitumor Effects in Vitro and in Vivo by Inhibiting Dihydroorotate Dehydrogenase and Inducing Reactive Oxygen Species Production (Journal of Medicinal Chemistry (2020) 63:14 (7633−7652) DOI: 10.1021/acs.jmedchem.0c00512). *Journal of Medicinal Chemistry* vol. 63 Preprint at https://doi.org/10.1021/acs.jmedchem.0c01455 (2020).

2. Madhavi Sastry, G., Adzhigirey, M., Day, T., Annabhimoju, R. & Sherman, W. Protein and ligand preparation: Parameters, protocols, and influence on virtual screening enrichments. *J Comput Aided Mol Des* 27, (2013).

3. Maestro, S. Schrödinger Release 2021-4. Preprint at (2021).

4. Lu, C. *et al.* OPLS4: Improving force field accuracy on challenging regimes of chemical space. *J Chem Theory Comput* 17, (2021).

5. LigPrep, S. Schrödinger Release 2021-4. Preprint at (2021).

6. Halgren, T. A. *et al.* Glide: A New Approach for Rapid, Accurate Docking and Scoring. 2. Enrichment Factors in Database Screening. *J Med Chem* 47, (2004).

7. Friesner, R. A. *et al.* Extra precision glide: Docking and scoring incorporating a model of hydrophobic enclosure for protein-ligand complexes. *J Med Chem* 49, (2006).

8. Bowers, K. J. *et al.* Scalable algorithms for molecular dynamics simulations on commodity clusters. in *Proceedings of the 2006 ACM/IEEE Conference on Supercomputing, SC'06* (2006). doi:10.1145/1188455.1188544.

9. D. E. Shaw Research. Desmond Molecular Dynamics System, Maestro-Desmond Interoperability Tools. Preprint at (2021).

10. Metropolis, N., Rosenbluth, A. W., Rosenbluth, M. N., Teller, A. H. & Teller, E. ARTICLES YOU MAY BE INTERESTED. *Studies in Molecular Dynamics. I. General Method The Journal of Chemical Physics* 21, (1953).

11. Martyna, G. J., Klein, M. L. & Tuckerman, M. Nosé-Hoover chains: The canonical ensemble via continuous dynamics. *J Chem Phys* 97, (1992).

12. Shinoda, W. & Mikami, M. Rigid-body dynamics in the isothermal-isobaric ensemble: A test on the accuracy and computational efficiency. *J Comput Chem* 24, (2003).

13. Morais, C. S. *et al.* Pyrazoline derivatives as promising novel antischistosomal agents. *Sci Rep* 11, (2021).

14. Sessa, D. P. *et al.* 15β-Senecioyl-oxy- ent-kaur-16-en-19-oic Acid, a Diterpene Isolated from Baccharis lateralis, as Promising Oral Compound for the Treatment of Schistosomiasis. *J Nat Prod* 83, (2020).

15. Rocha, V. C. *et al.* Evaluating the Antischistosomal Activity of Dehydrodieugenol B and Its Methyl Ether Isolated from Nectandra leucantha─A Preclinical Study against Schistosoma mansoni Infection. *ACS Omega* 8, (2023).

16. Brito, J. R. *et al.* Neolignans isolated from Saururus cernuus L. (Saururaceae) exhibit efficacy against Schistosoma mansoni. *Sci Rep* 12, (2022).

17. Purificação, A. D. et al. Unveiling the Antiviral Capabilities of Targeting Human Dihydroorotate Dehydrogenase against SARS-CoV-2. ACS Omega 9, 11418–11430 (2024).

NMR SPECTRA

NMR (300 MHz, DMSO-d_6) of 1,4-dihydroquinoxaline-2,3-dione (**2**)

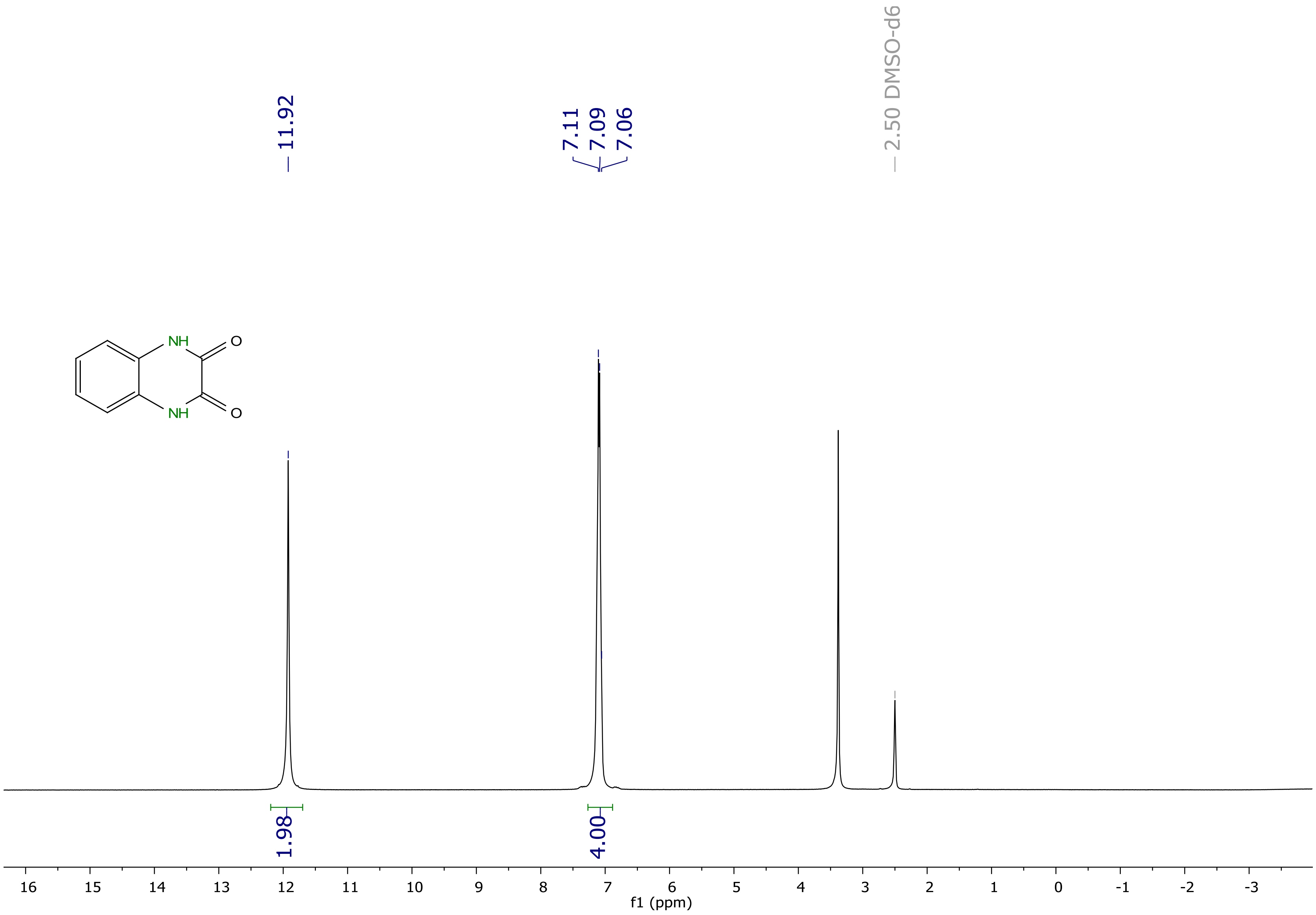

^{13}C NMR (75 MHz, DMSO-d_6) of 1,4-dihydroquinoxaline-2,3-dione (**2**)

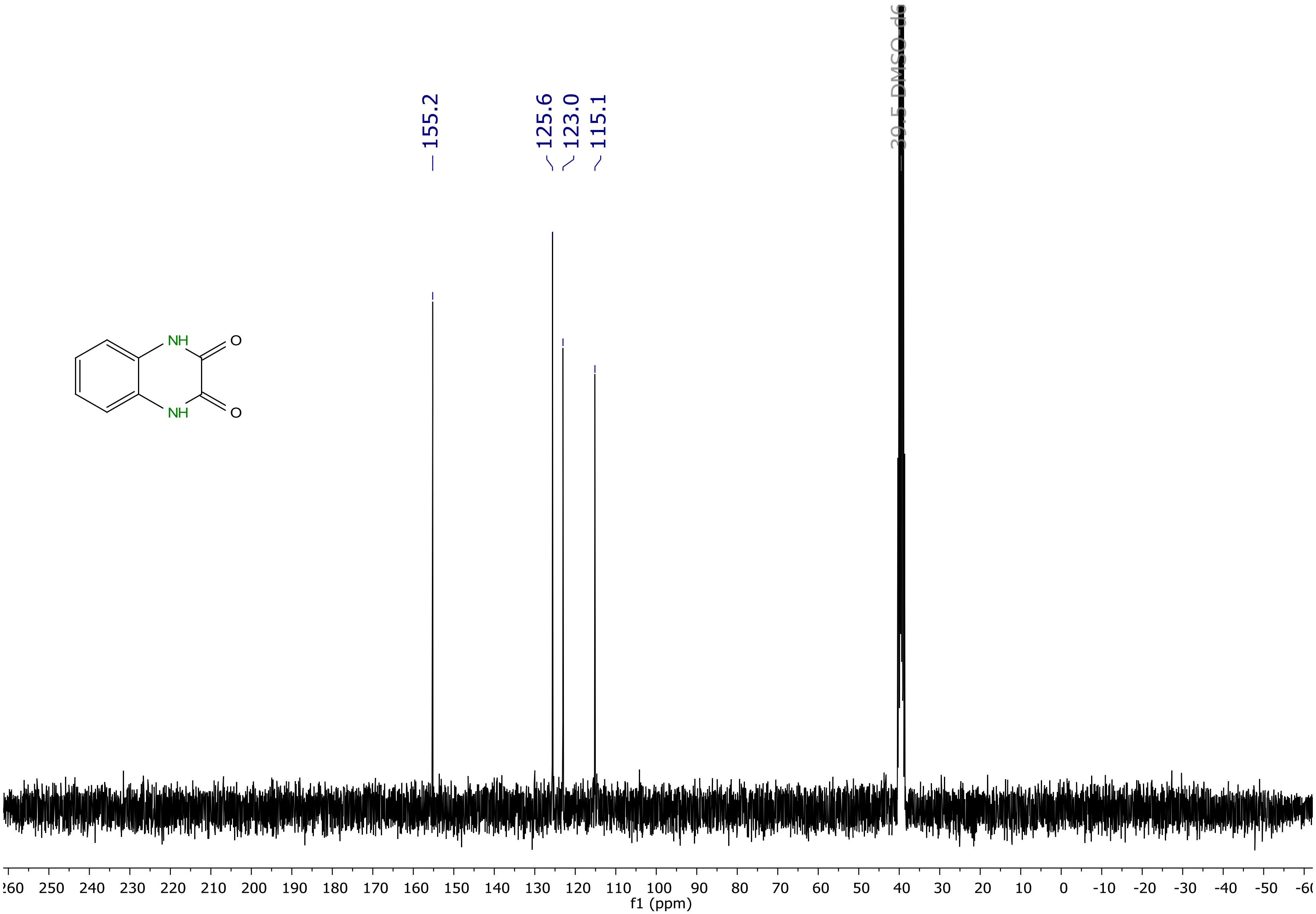

^{1}H NMR (300 MHz, DMSO-d_6) of 2,3-dichloroquinoxaline (**3**)

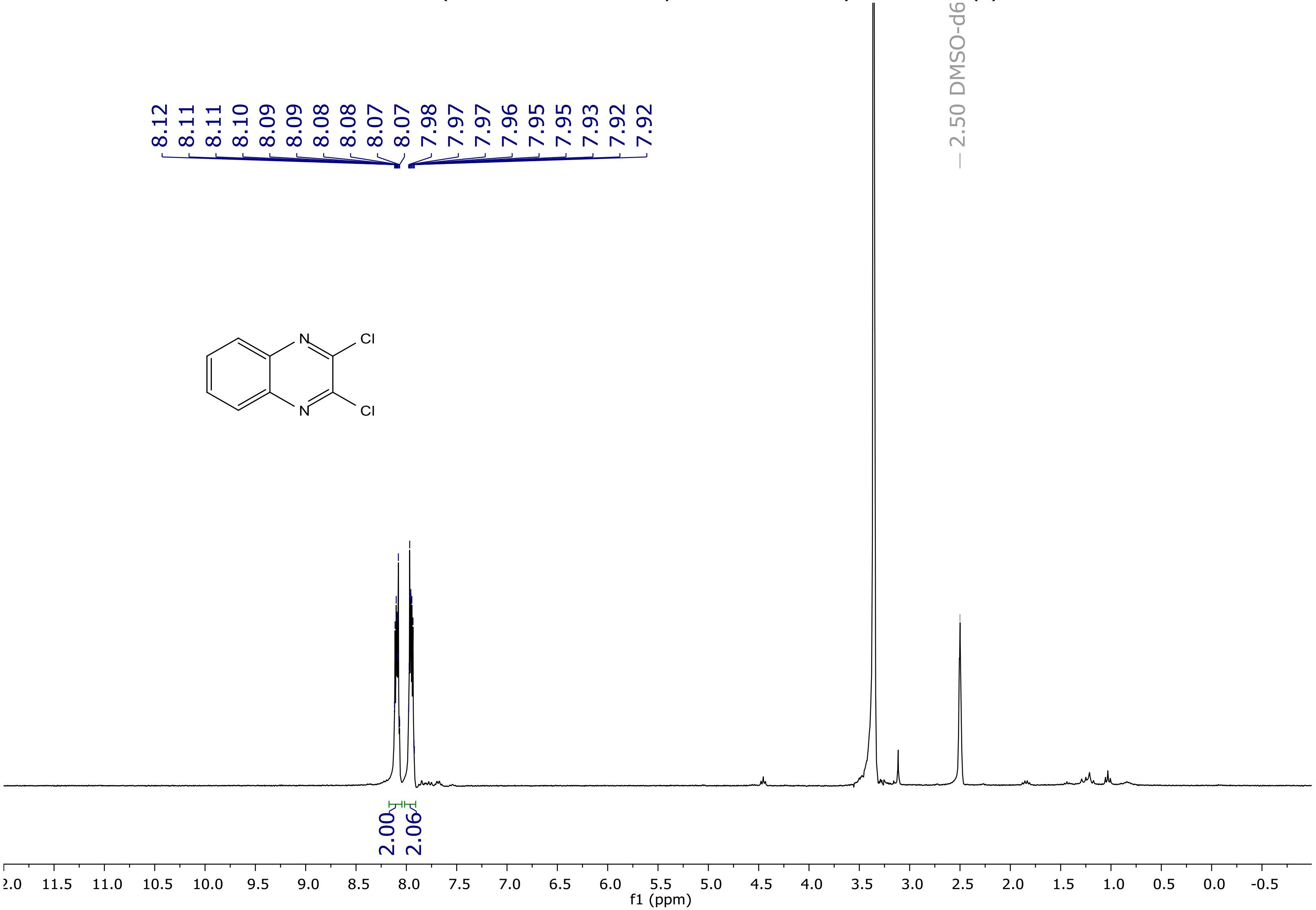

^{13}C NMR (75 MHz, DMSO-d_6) of 2,3-dichloroquinoxaline (**3**)

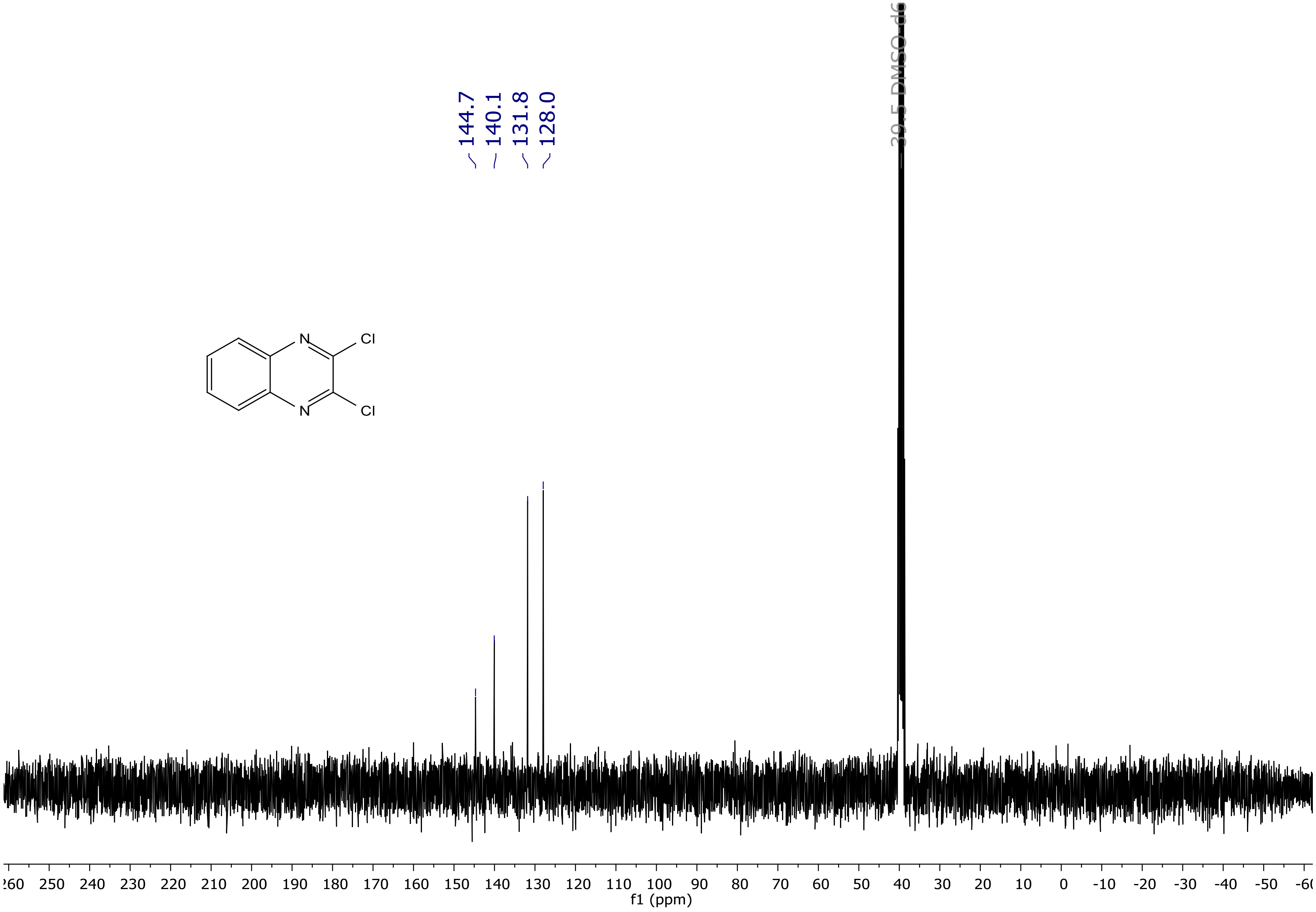

^1H NMR (300 MHz, DMSO-d_6) of 3-chloroquinoxalin-2(1*H*)-one (**4**)

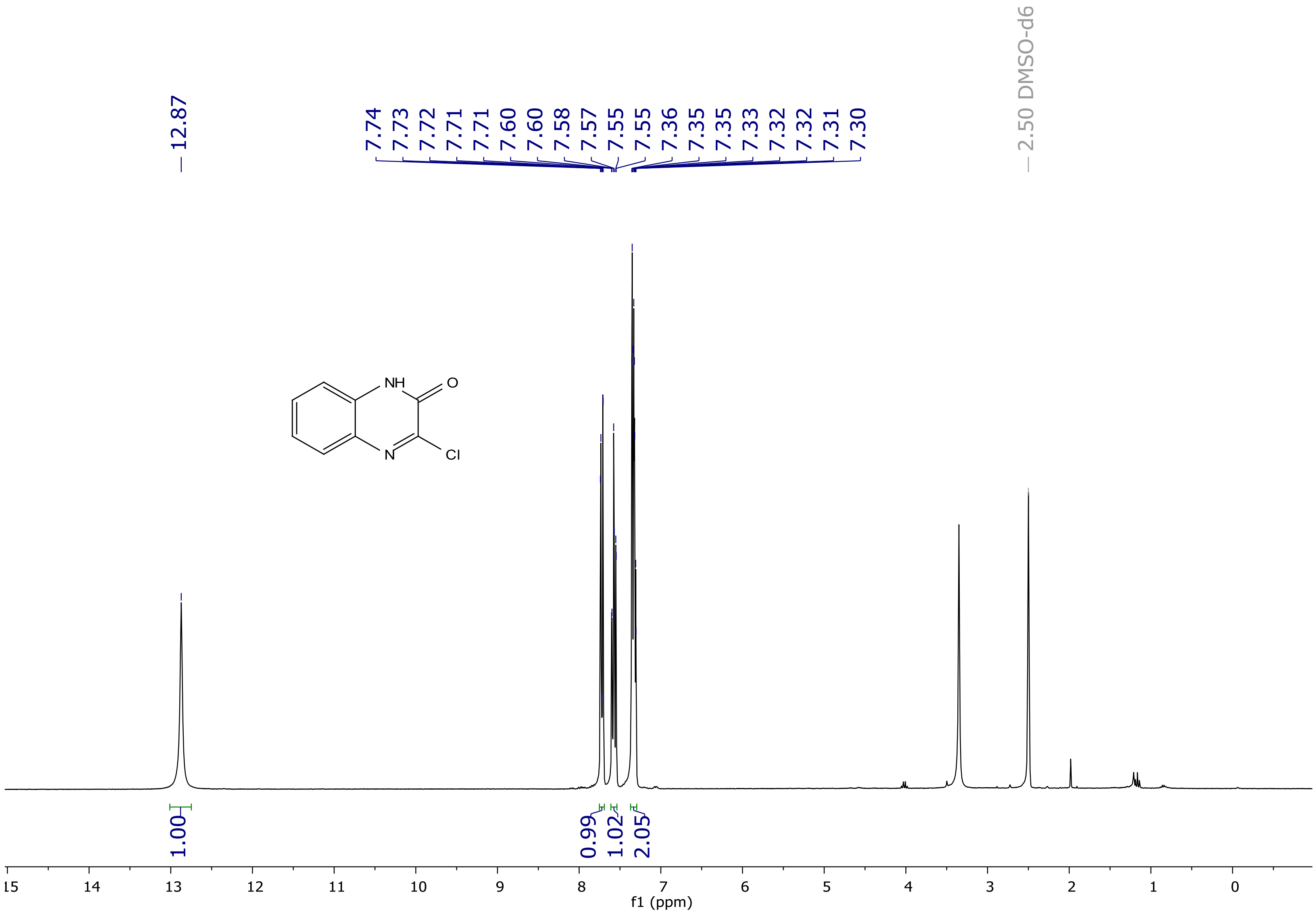

^{13}C NMR (75 MHz, DMSO-d_6) of 3-chloroquinoxalin-2(1*H*)-one (**4**)

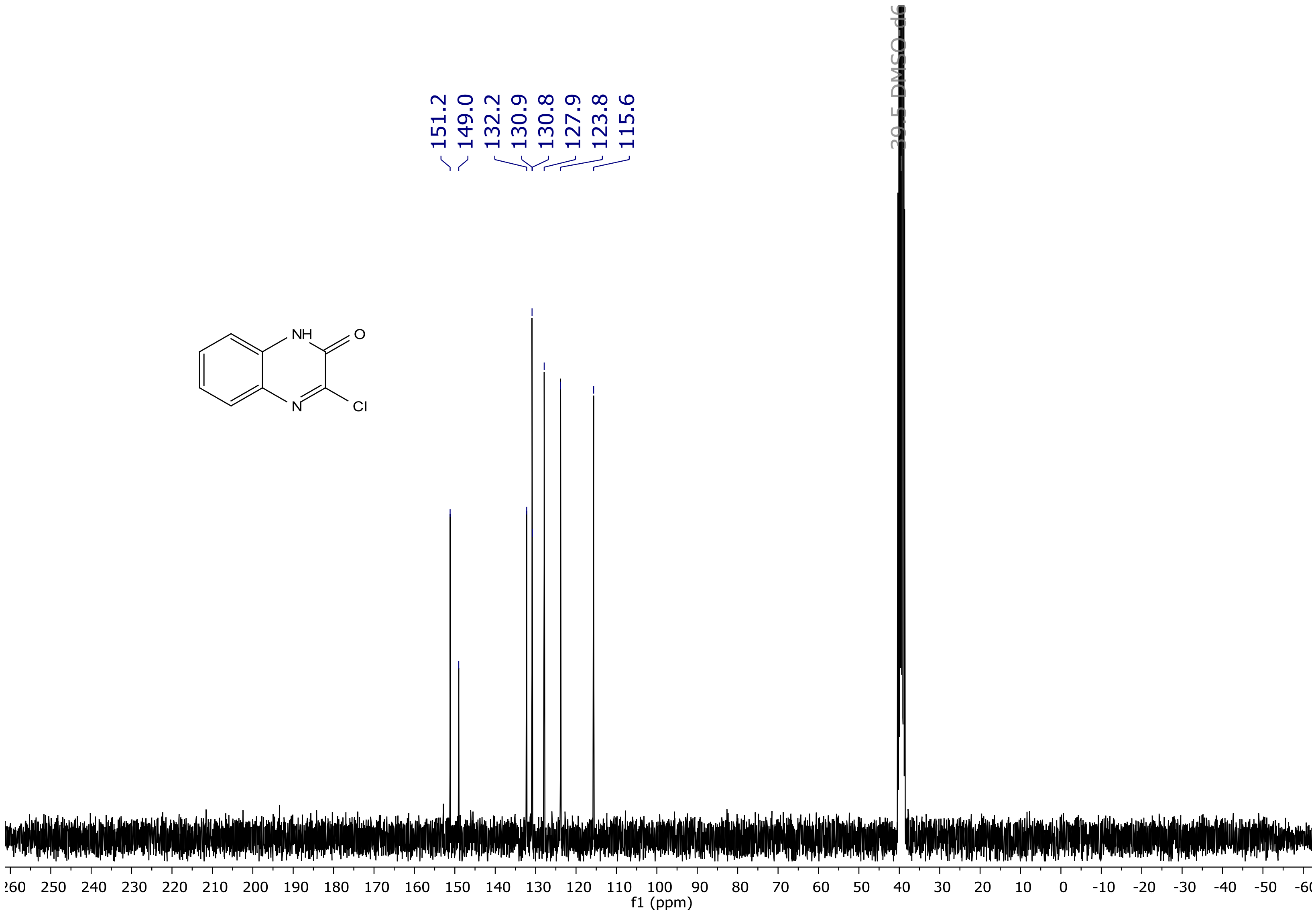

^{1}H NMR (300 MHz, DMSO-d_6) of 3-((4-methoxyphenyl)amino)quinoxalin-2(1H)-one (**5a**)

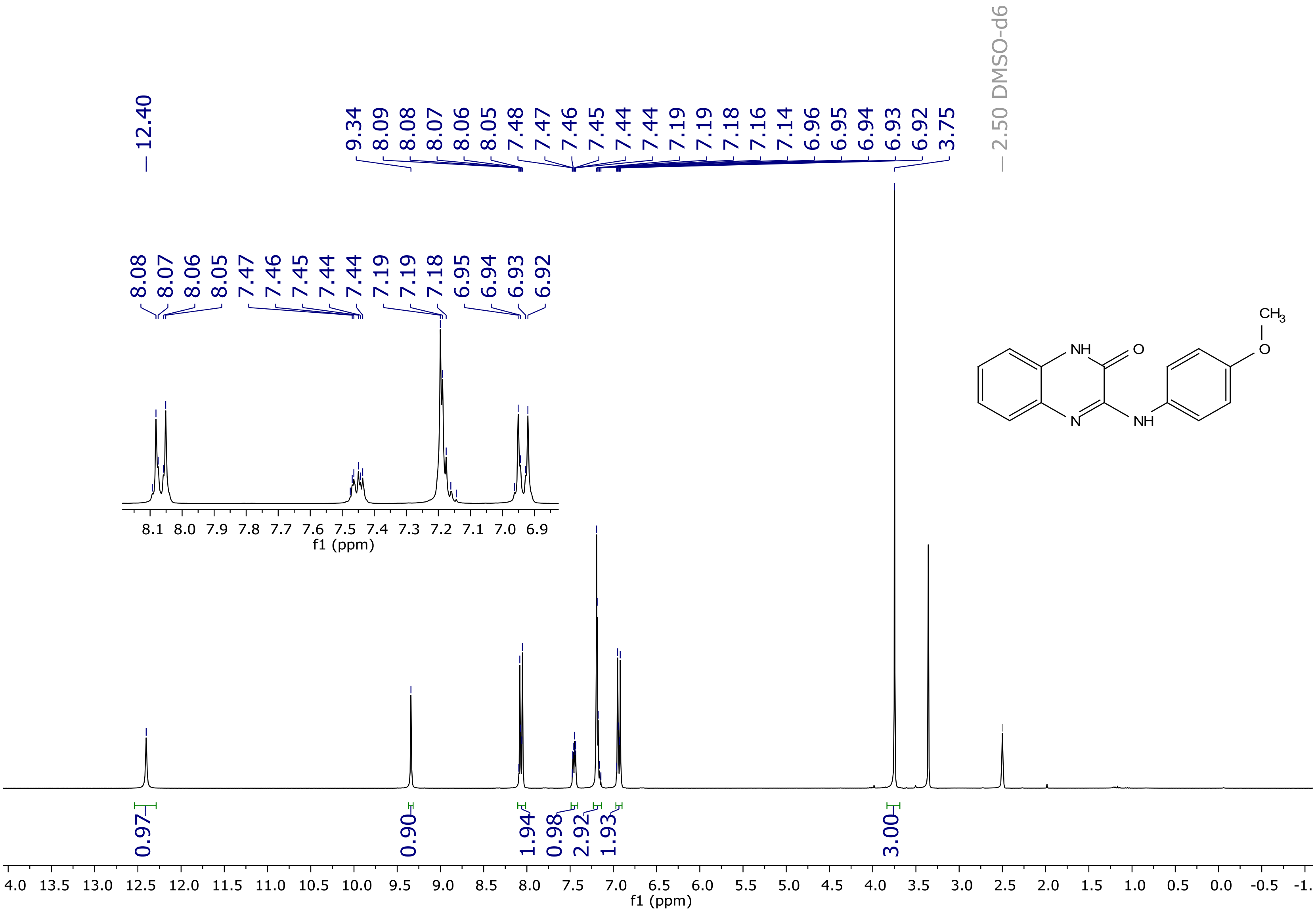

^{13}C NMR (75 MHz, DMSO-d_6) of 3-((4-methoxyphenyl)amino)quinoxalin-2(1*H*)-one (**5a**)

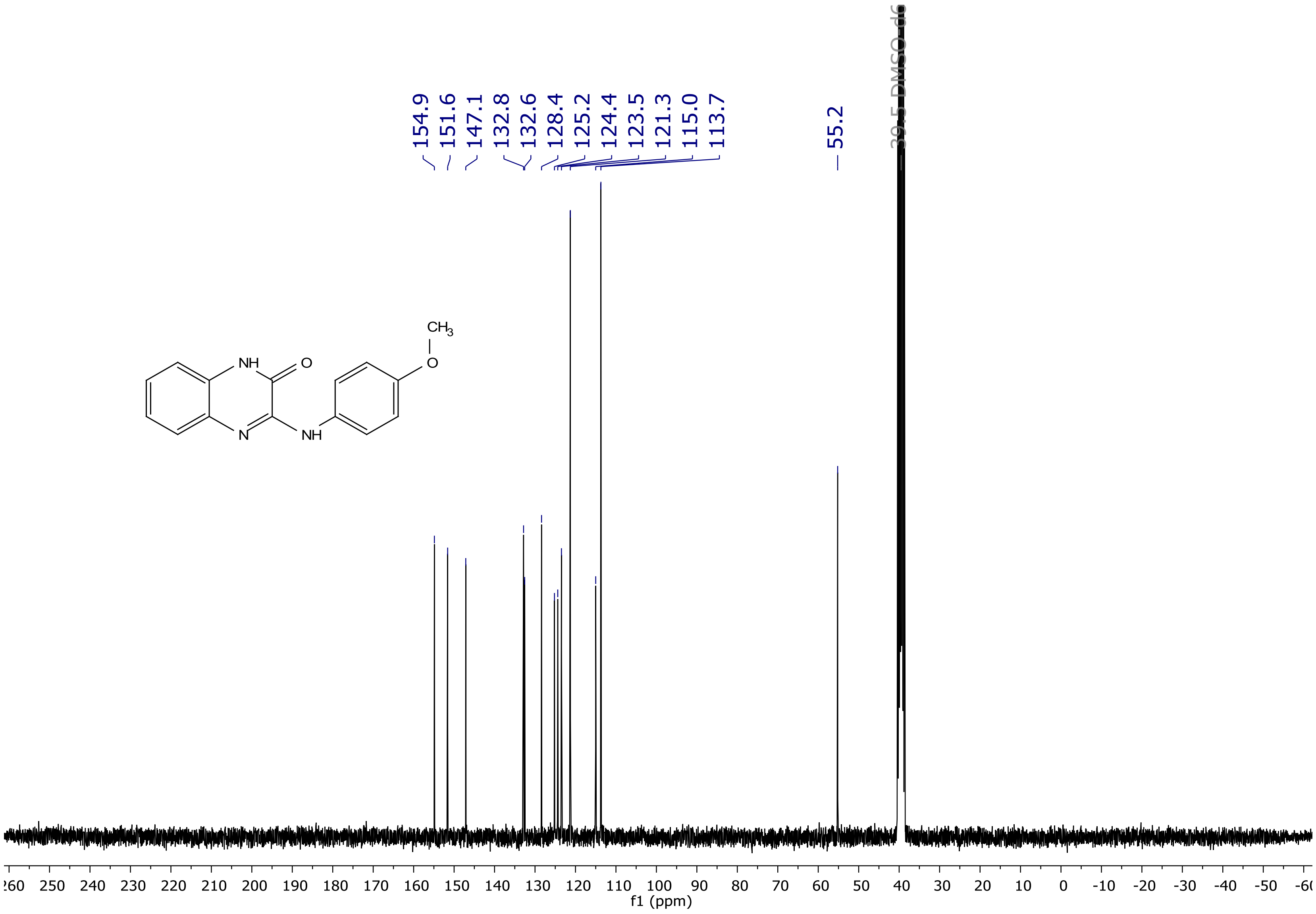

FTIR spectra of 3-((4-methoxyphenyl)amino)quinoxalin-2(1*H*)-one (**5a**)

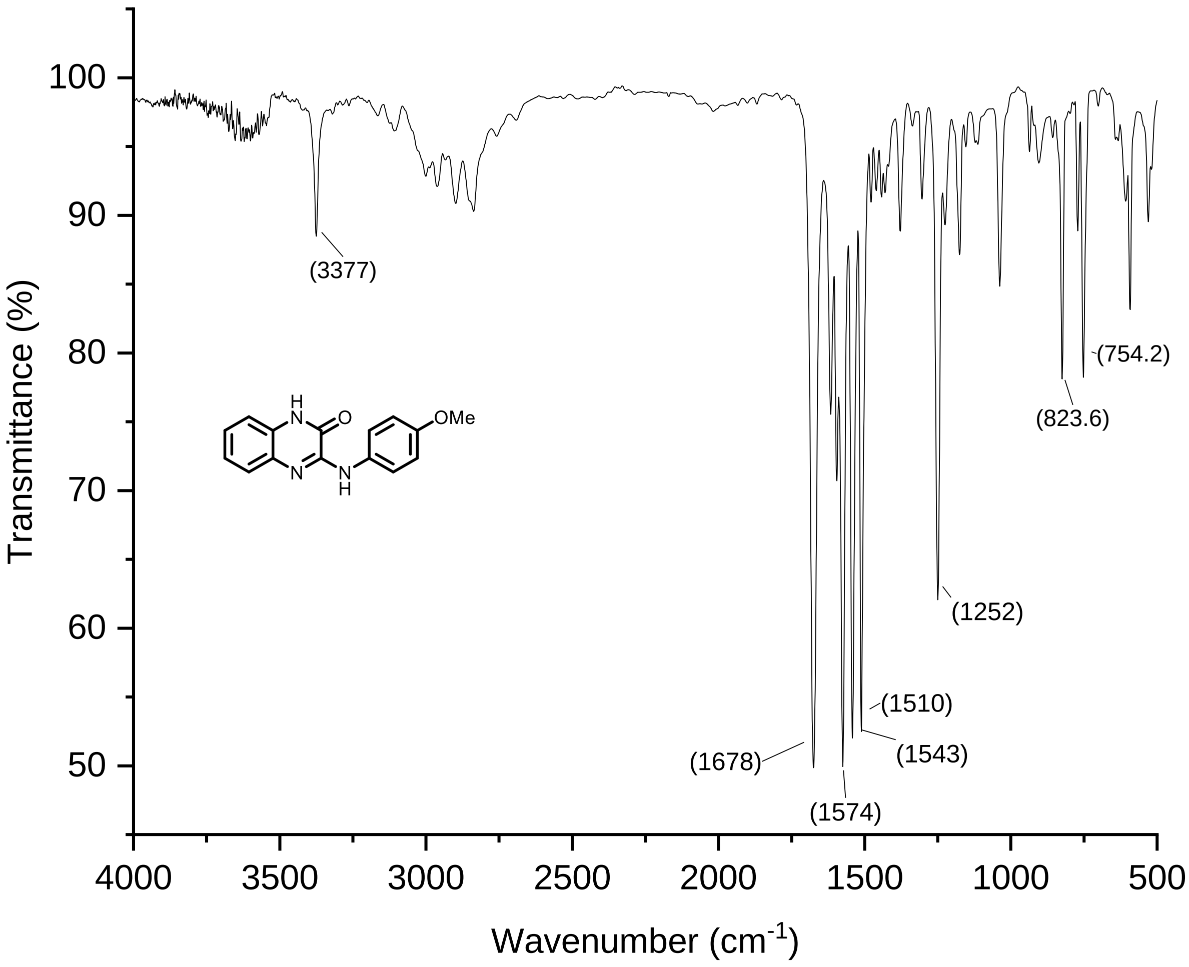

^{1}H NMR (300 MHz, DMSO-d_6) of 3-((4-chlorophenyl)amino)quinoxalin-2(1*H*)-one (**5b**)

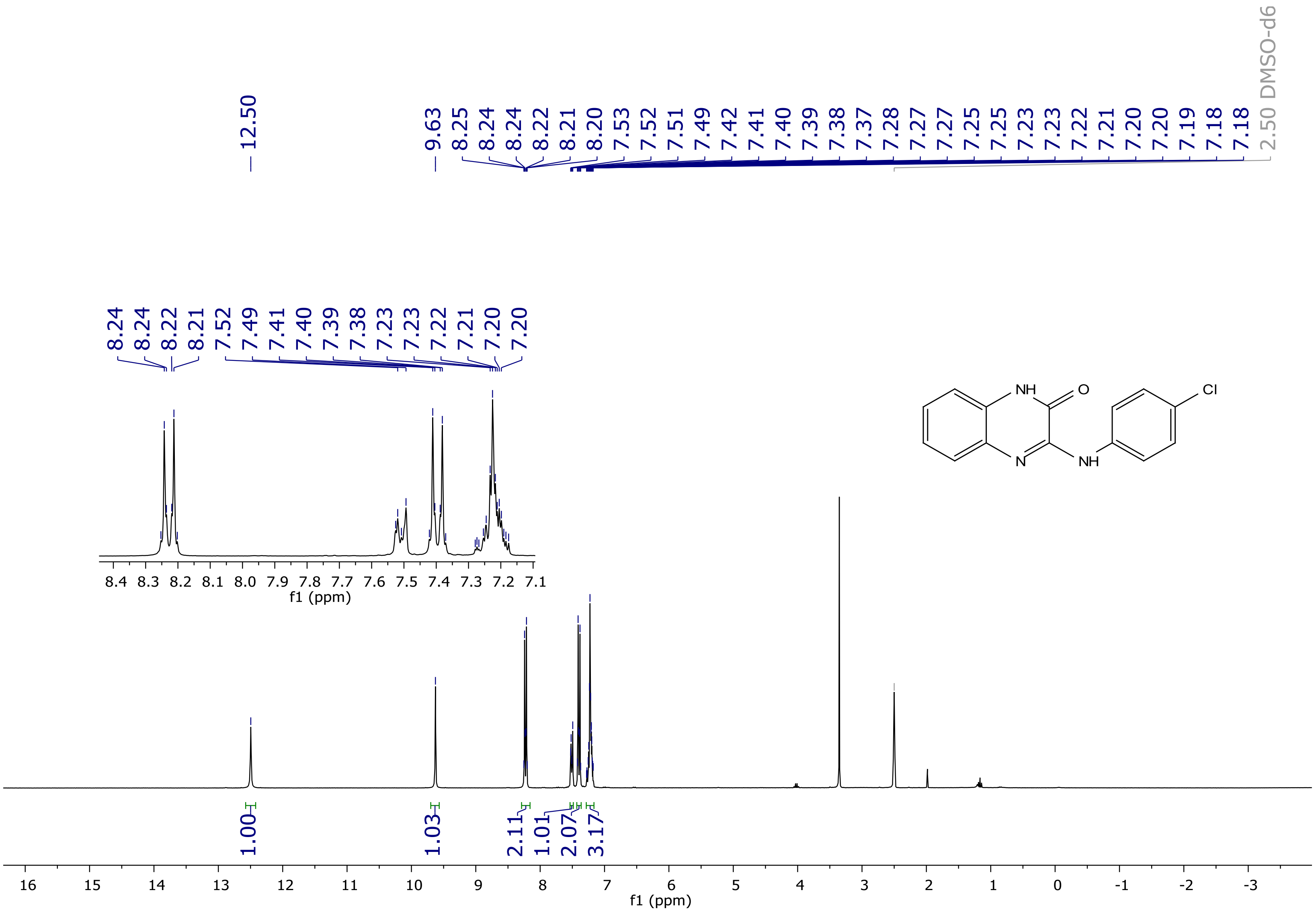

^{13}C NMR (75 MHz, DMSO-d_6) spectrum of 3-((4-chlorophenyl)amino)quinoxalin-2(1*H*)-one (**5b**)

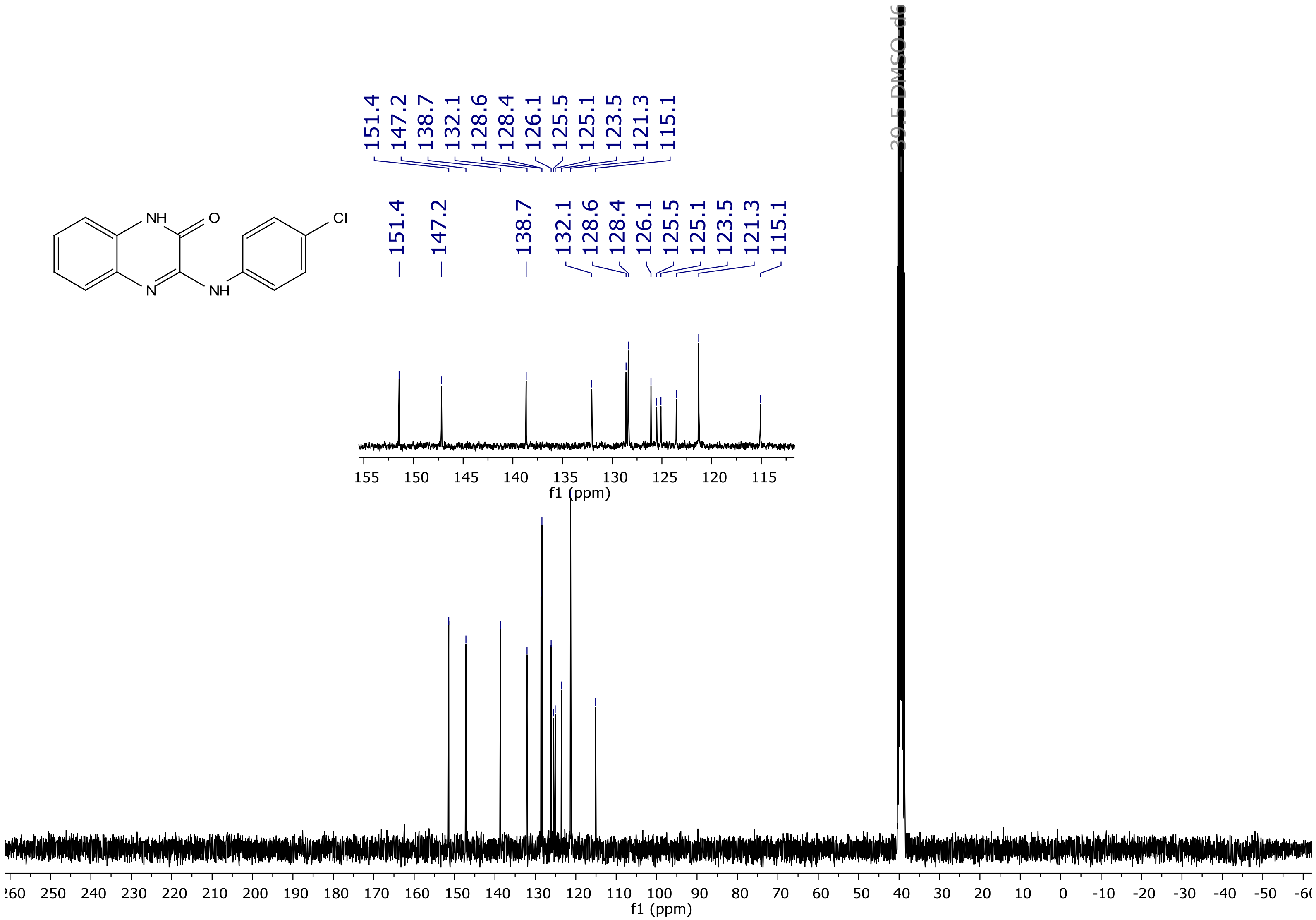

^{1}H NMR (300 MHz, DMSO-d_6) spectrum of 3-((4-fluorophenyl)amino)quinoxalin-2(1*H*)-one (**5c**)

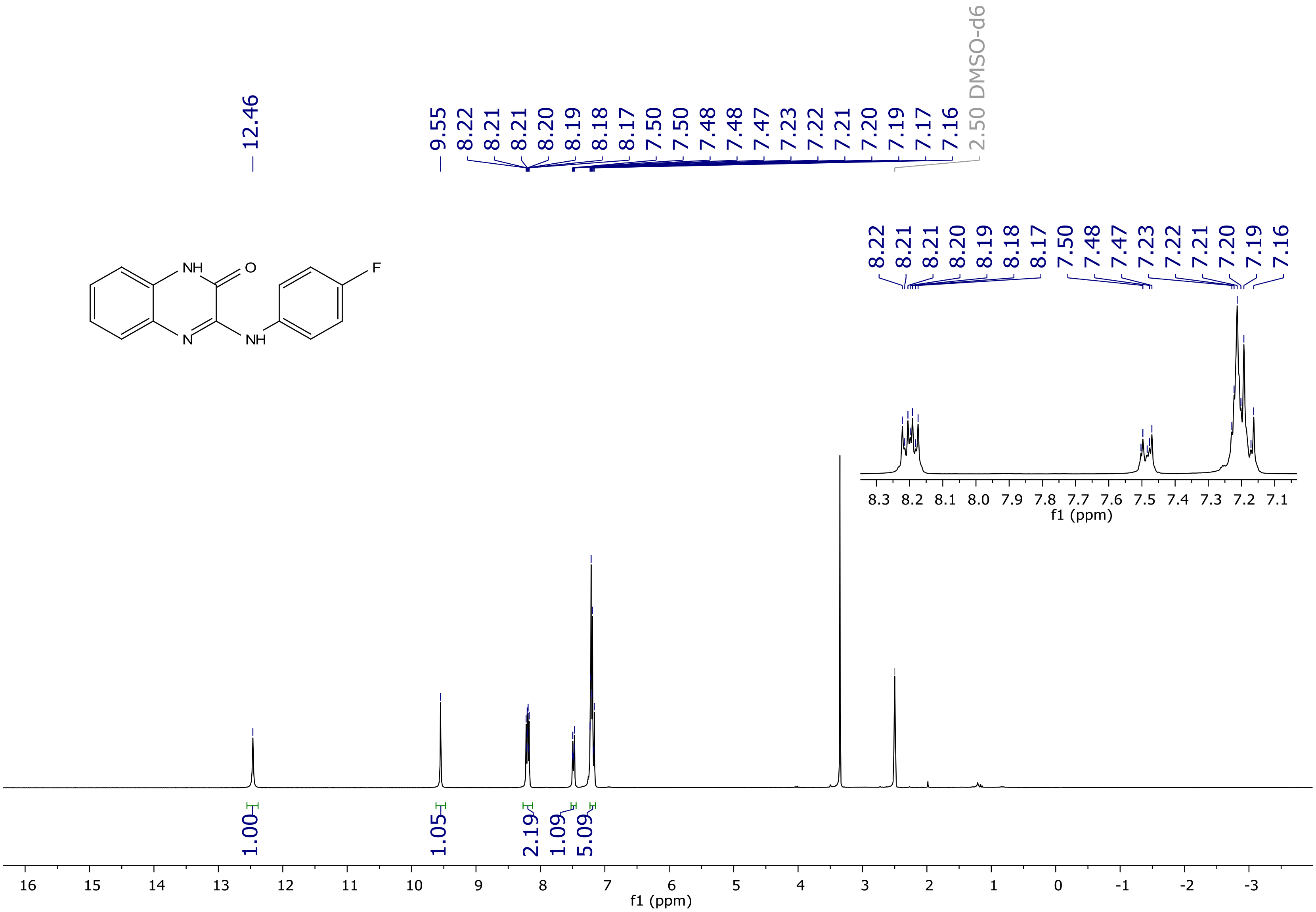

^{13}C NMR (75 MHz, DMSO-d_6) spectrum of 3-((4-fluorophenyl)amino)quinoxalin-2(1*H*)-one (**5c**)

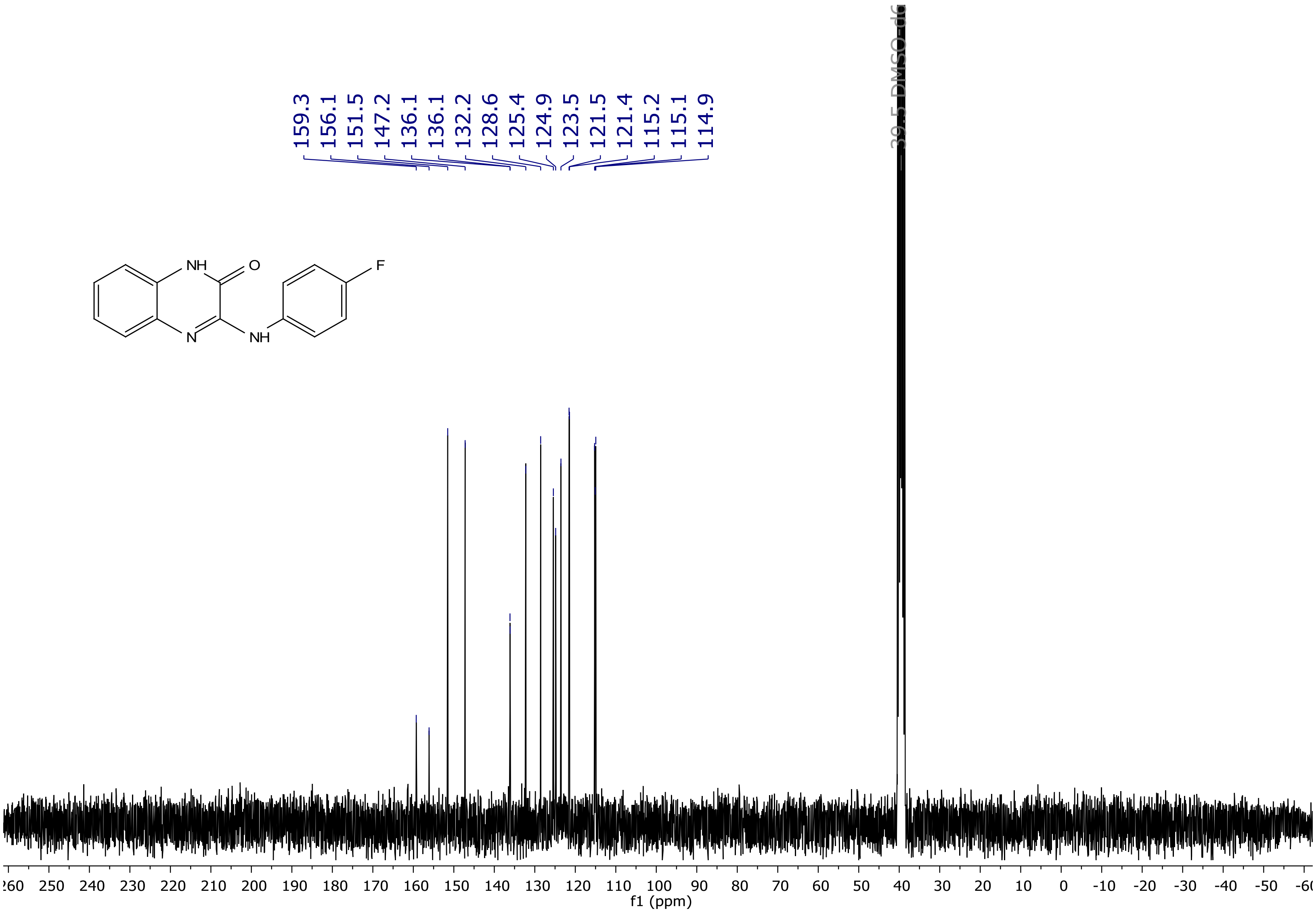

^{1}H NMR (300 MHz, DMSO-*d*6) of 3-((2,3-dihydrobenzo[*b*][1,4]dioxin-6-yl)amino)quinoxalin-2(1*H*)-one (**5d**)

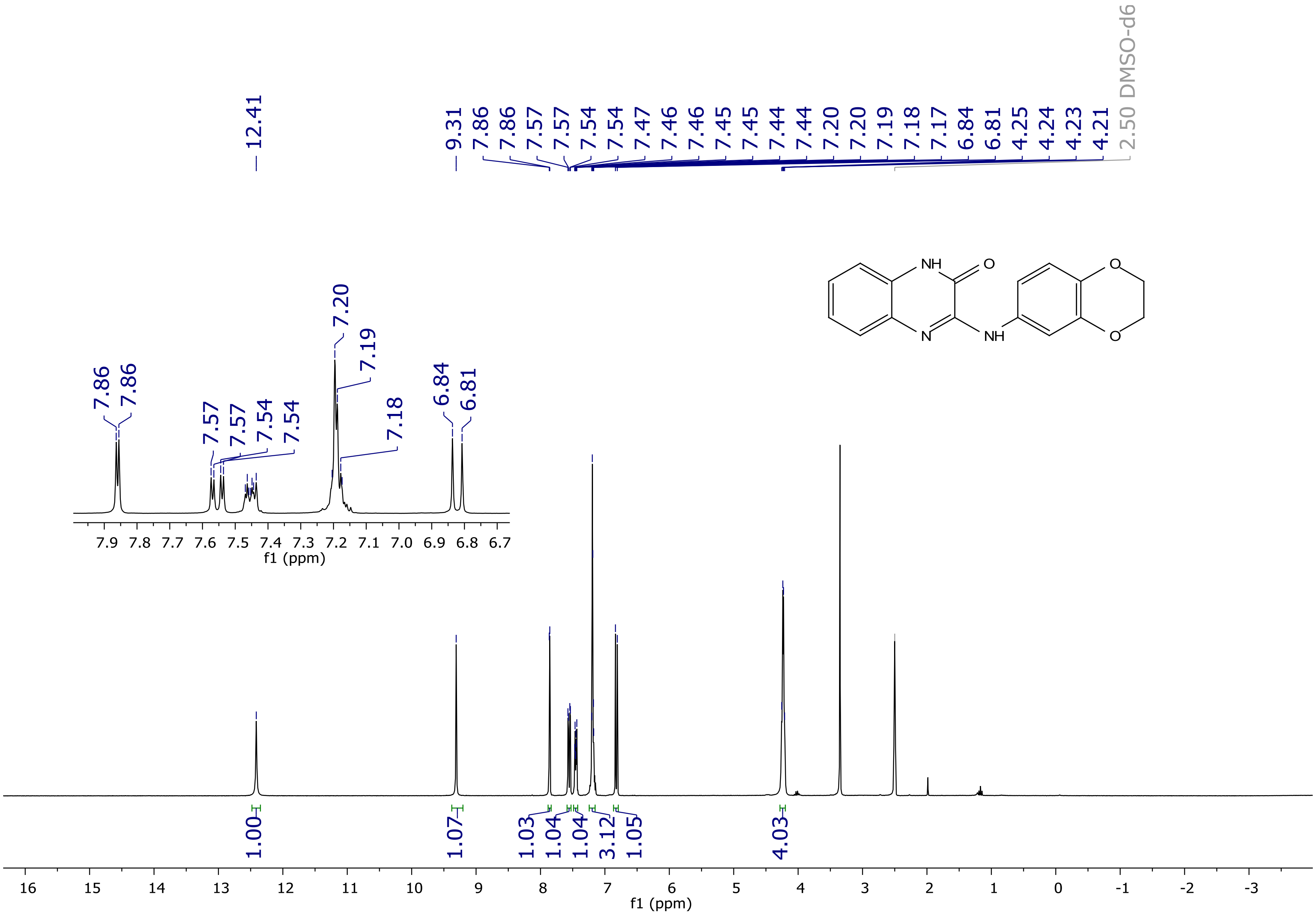

^{13}C NMR (75 MHz, DMSO-d_6) spectrum of 3-((2,3-dihydrobenzo[*b*][1,4]dioxin-6-yl)amino)quinoxalin-2(1*H*)-one (**5d**)

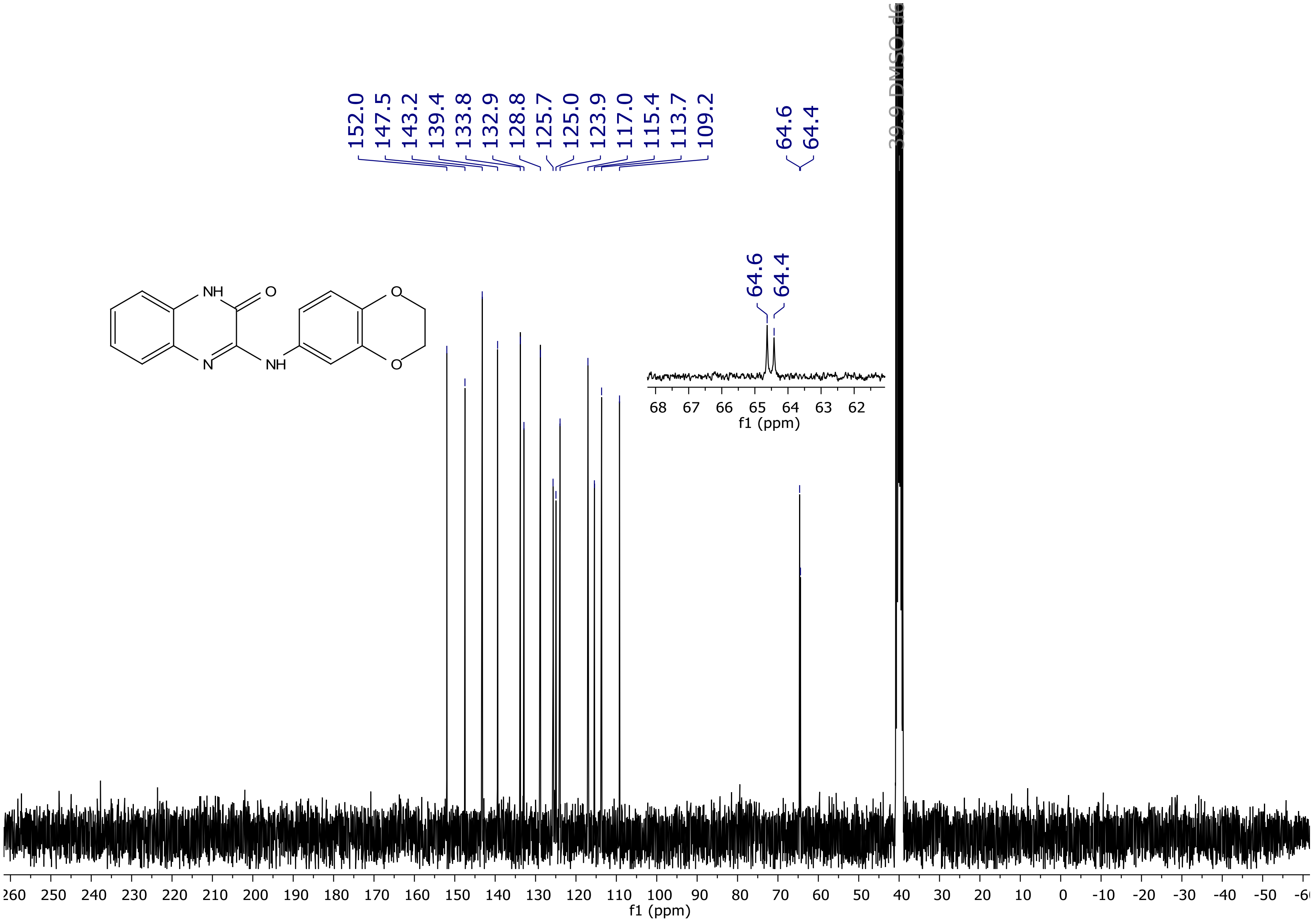

FTIR spectra of 3-((2,3-dihydrobenzo[*b*][1,4]dioxin-6-yl)amino)quinoxalin-2(1*H*)-one (**5d**)

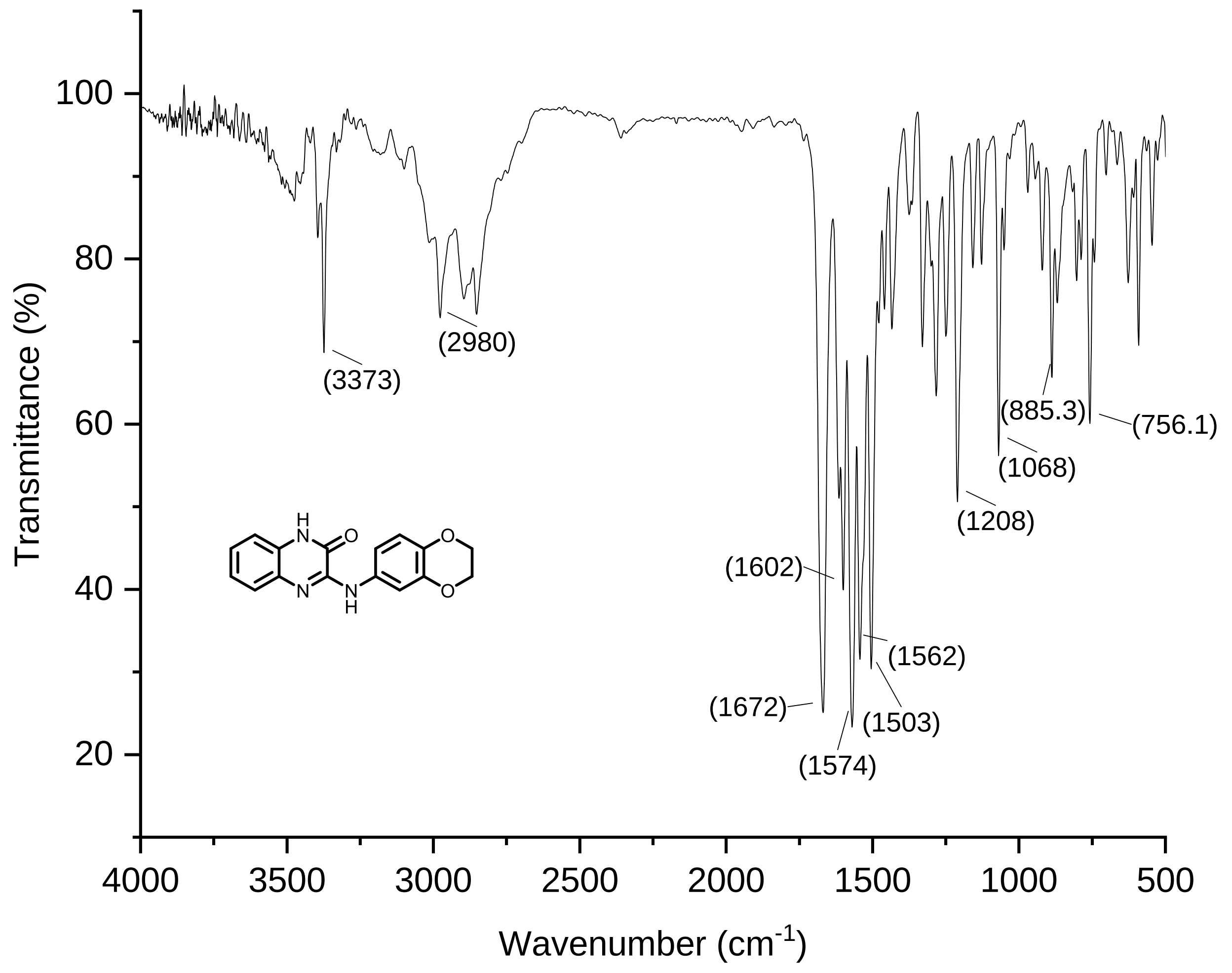

^{1}H NMR (300 MHz, DMSO-d_6) of quinazoline-2,4-diol (**7**)

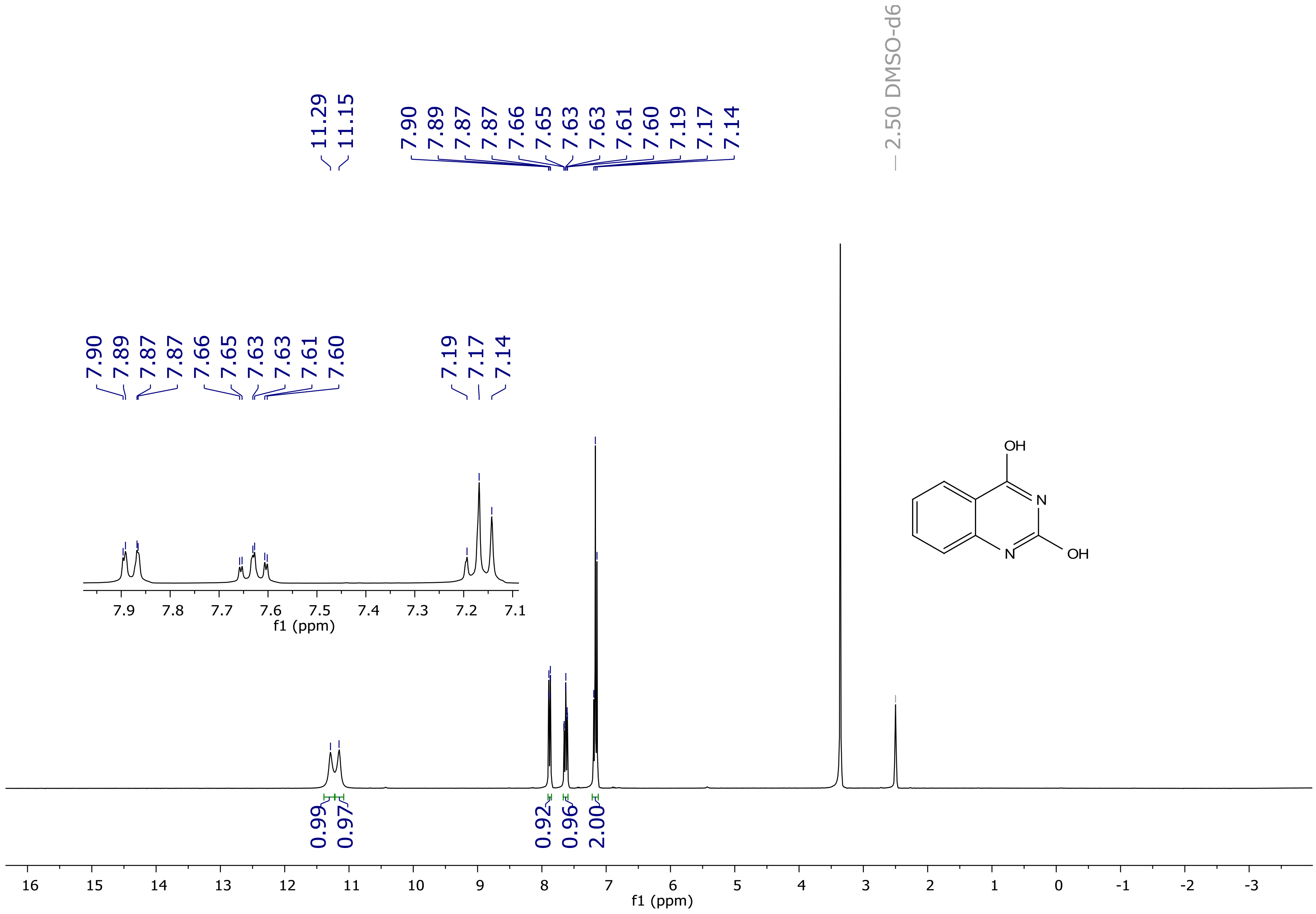

^{13}C NMR (75 MHz, DMSO-d_6) of quinazoline-2,4-diol (**7**)

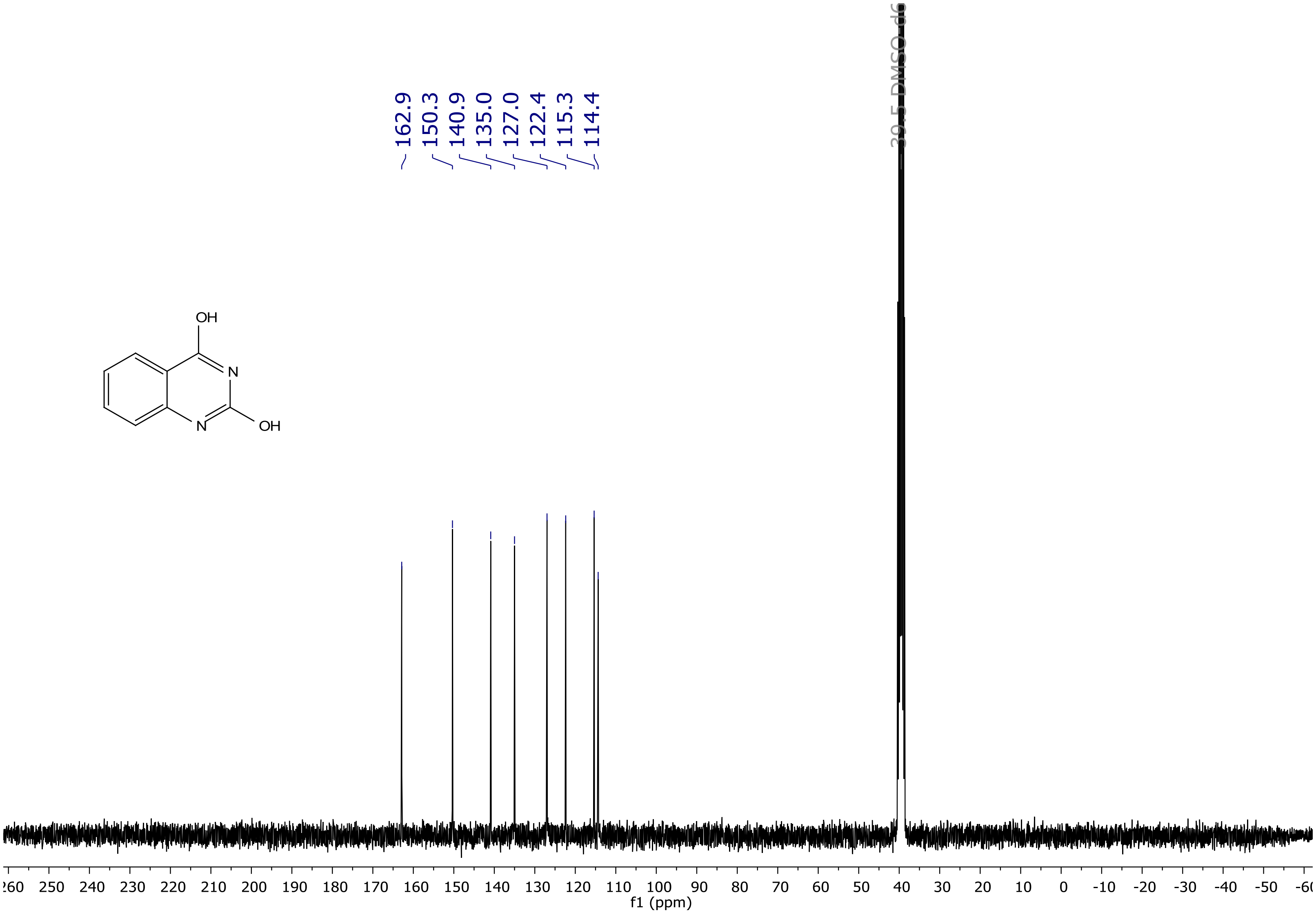

^{1}H NMR (300 MHz, DMSO-d_6) of 2,4-dichloroquinazoline (**8**)

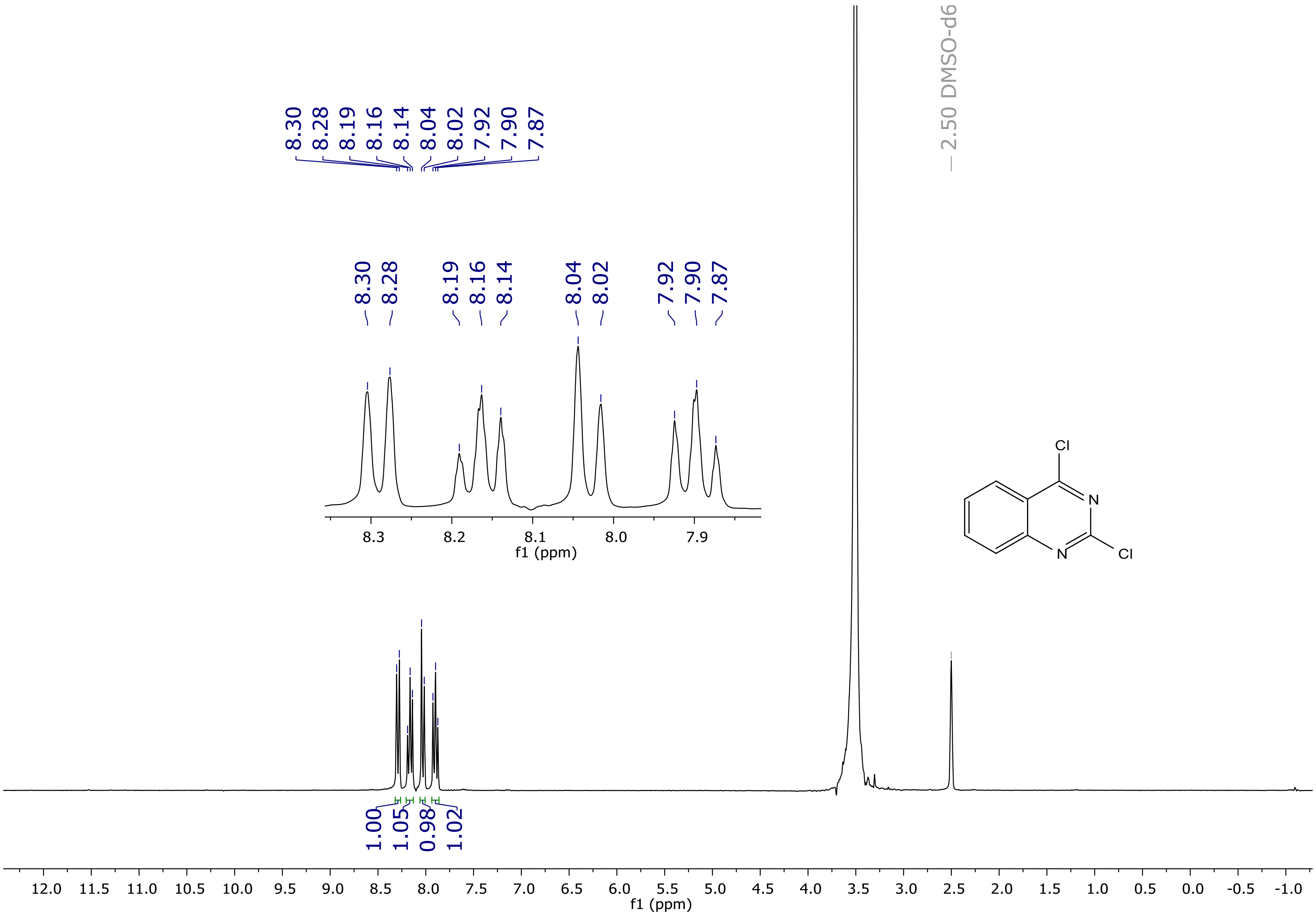

^{1}H NMR (300 MHz, DMSO-d_6) of 2-chloroquinazolin-4(3*H*)-one (**9**)

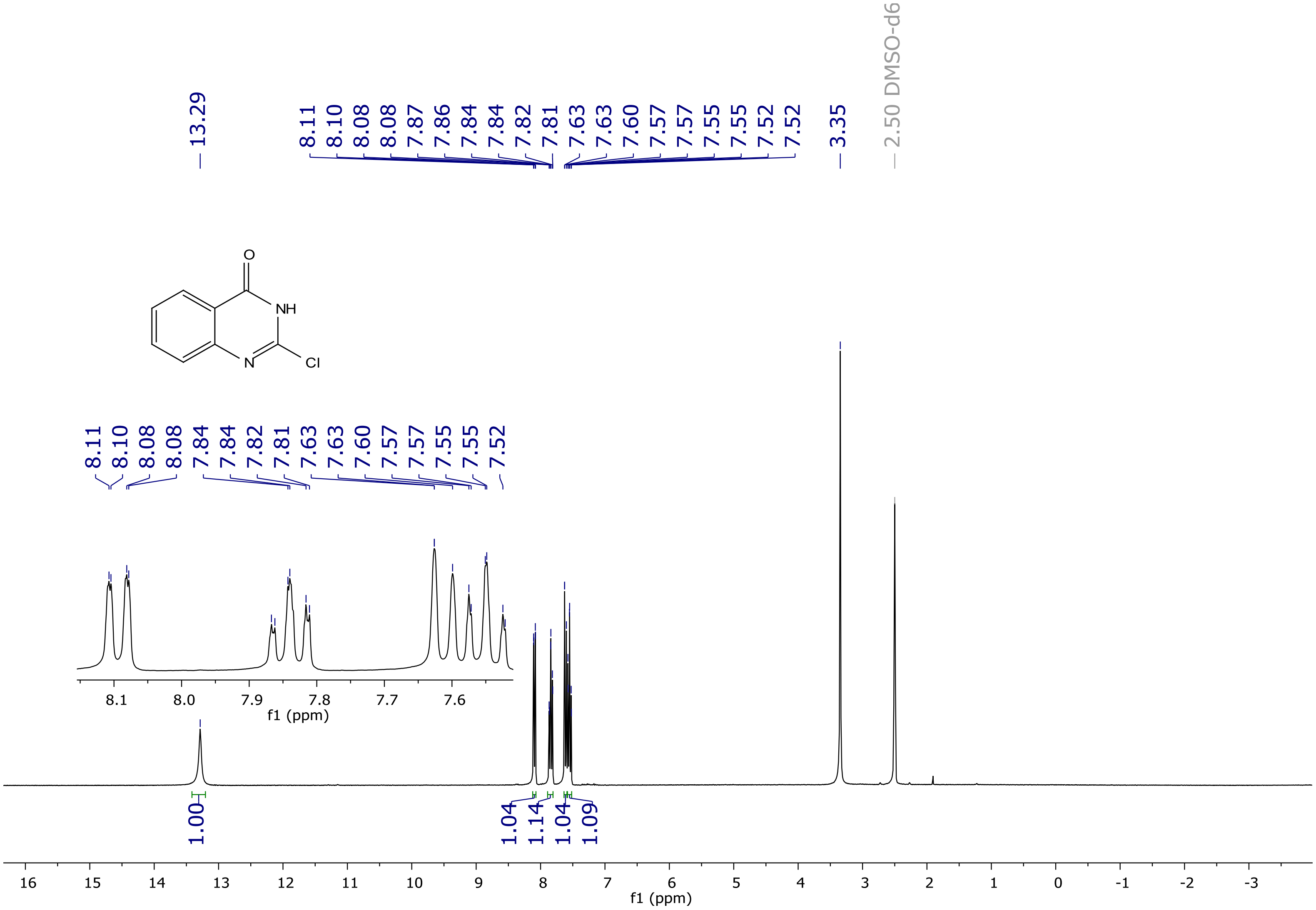

^{13}C NMR (75 MHz, DMSO-d_6) of 2-chloroquinazolin-4(3*H*)-one (**9**)

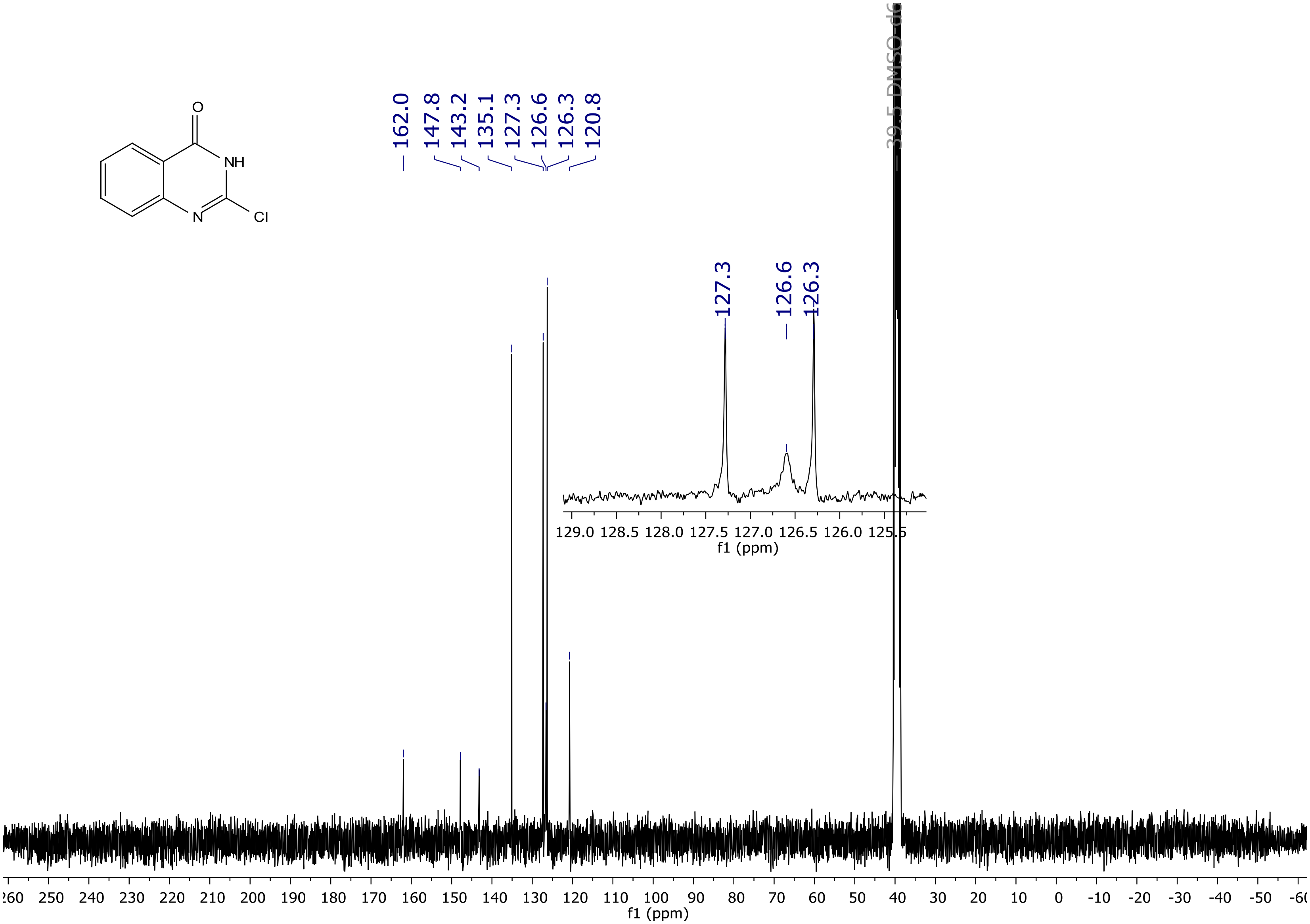

^{1}H NMR (300 MHz, DMSO-d_6) of 2-((4-methoxyphenyl)amino)quinazolin-4(3H)-one (**10a**)

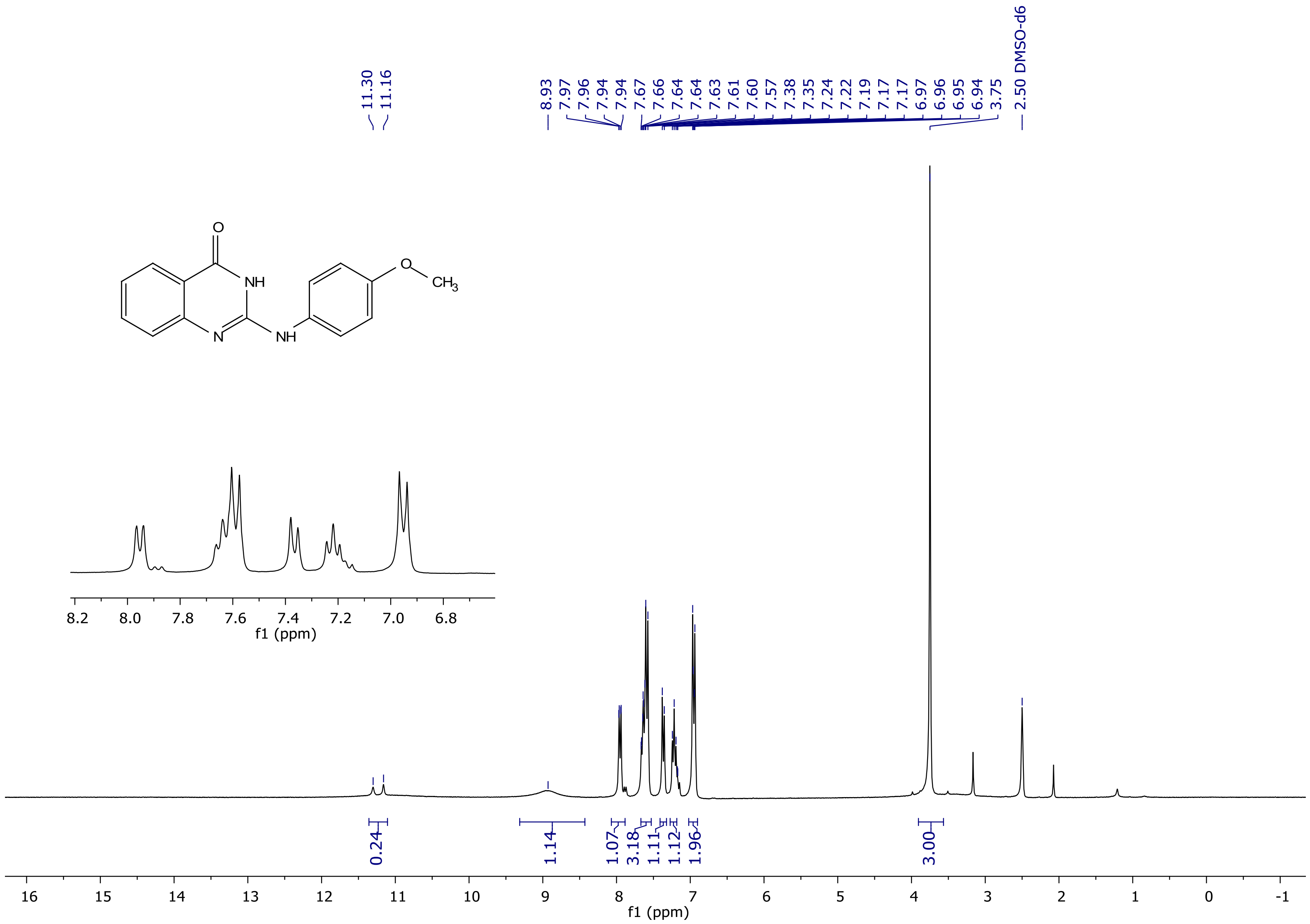

^{13}C NMR (101 MHz, DMSO-d_6) of 2-((4-methoxyphenyl)amino)quinazolin-4(3H)-one (**10a**)

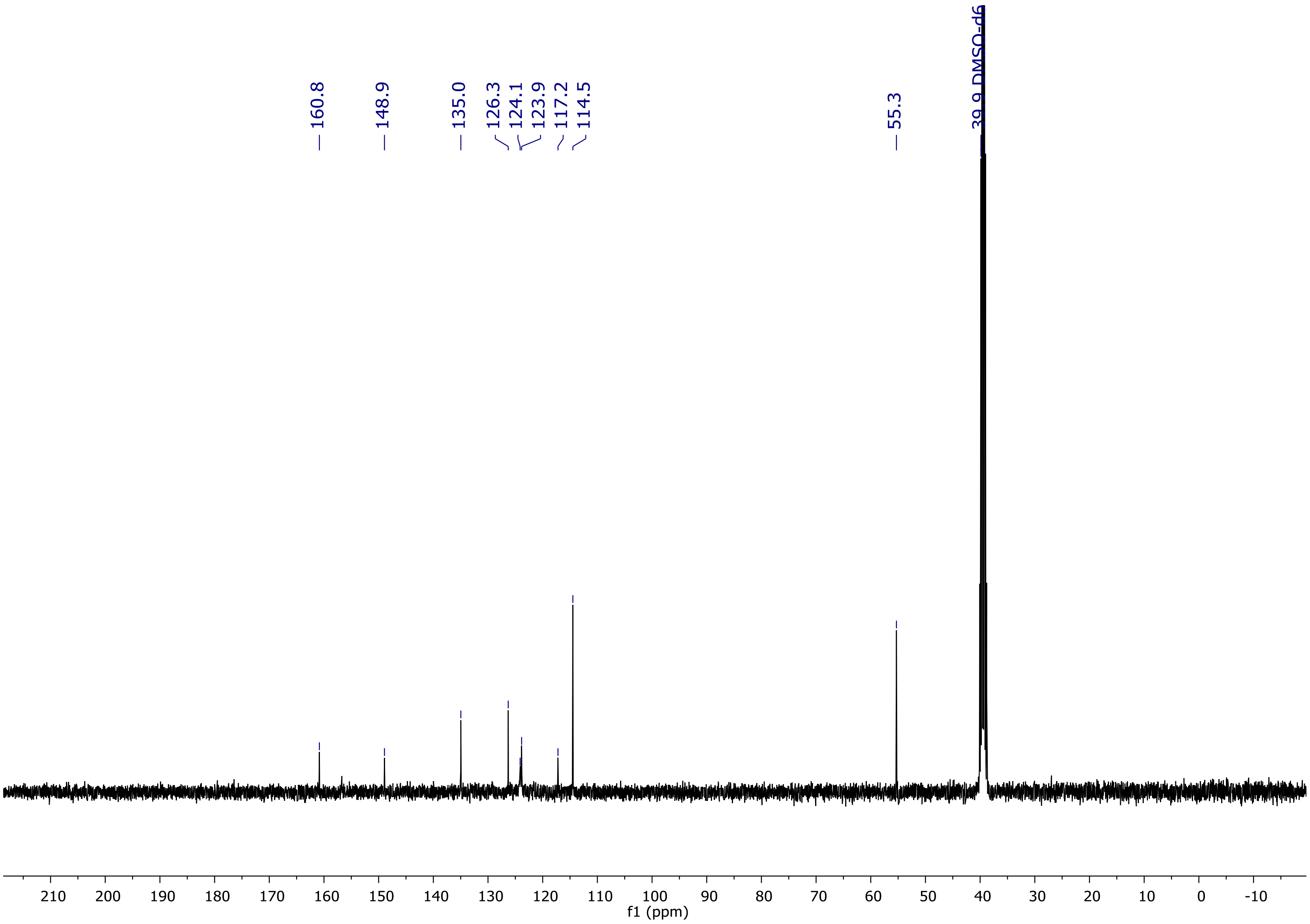

^1H NMR (300 MHz, DMSO-d_6) of 2-((4-fluorophenyl)amino)quinazolin-4(3*H*)-one (**10b**)

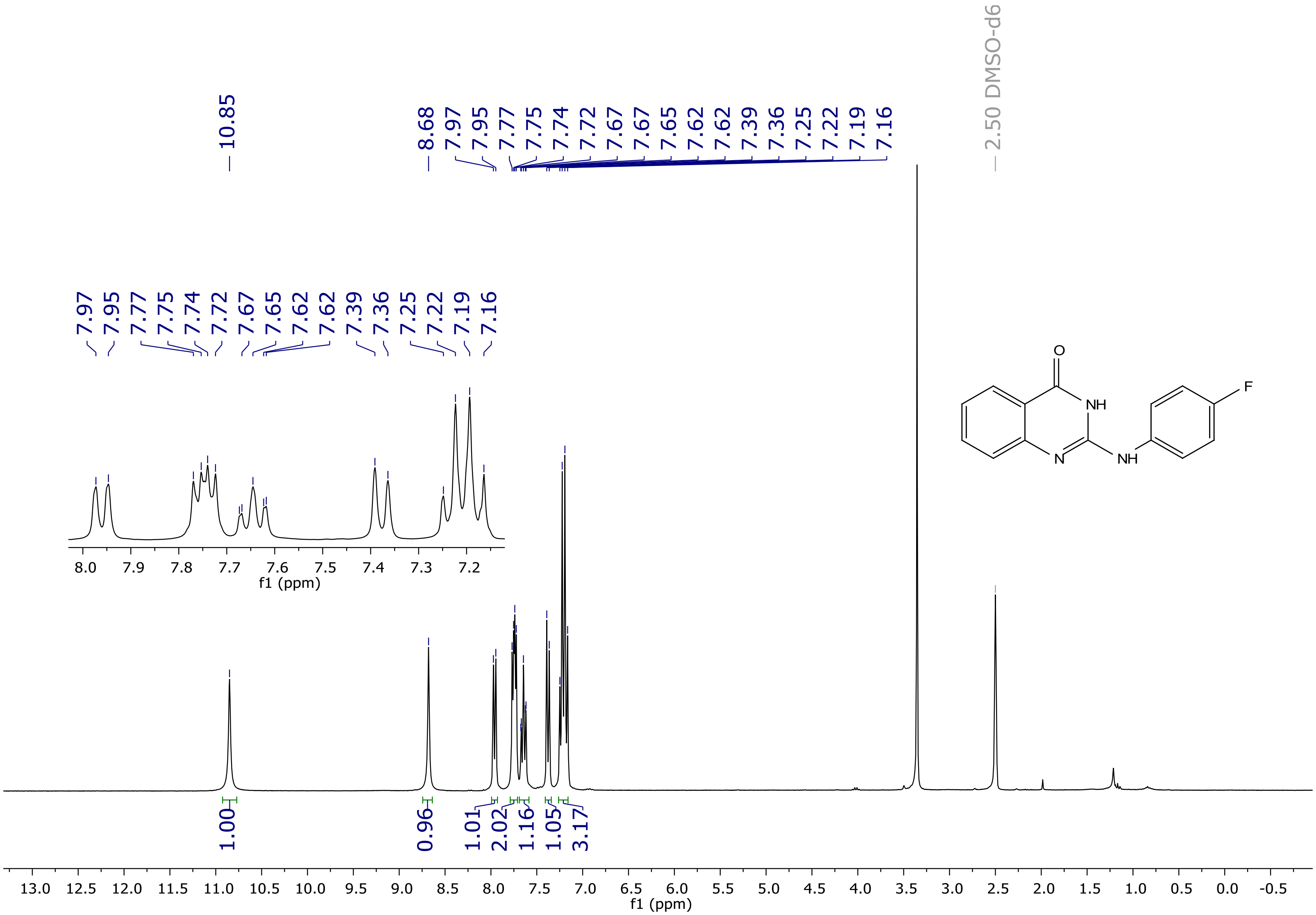

^{13}C NMR (75 MHz, DMSO-d_6) of 2-((4-fluorophenyl)amino)quinazolin-4(3*H*)-one (**10b**)

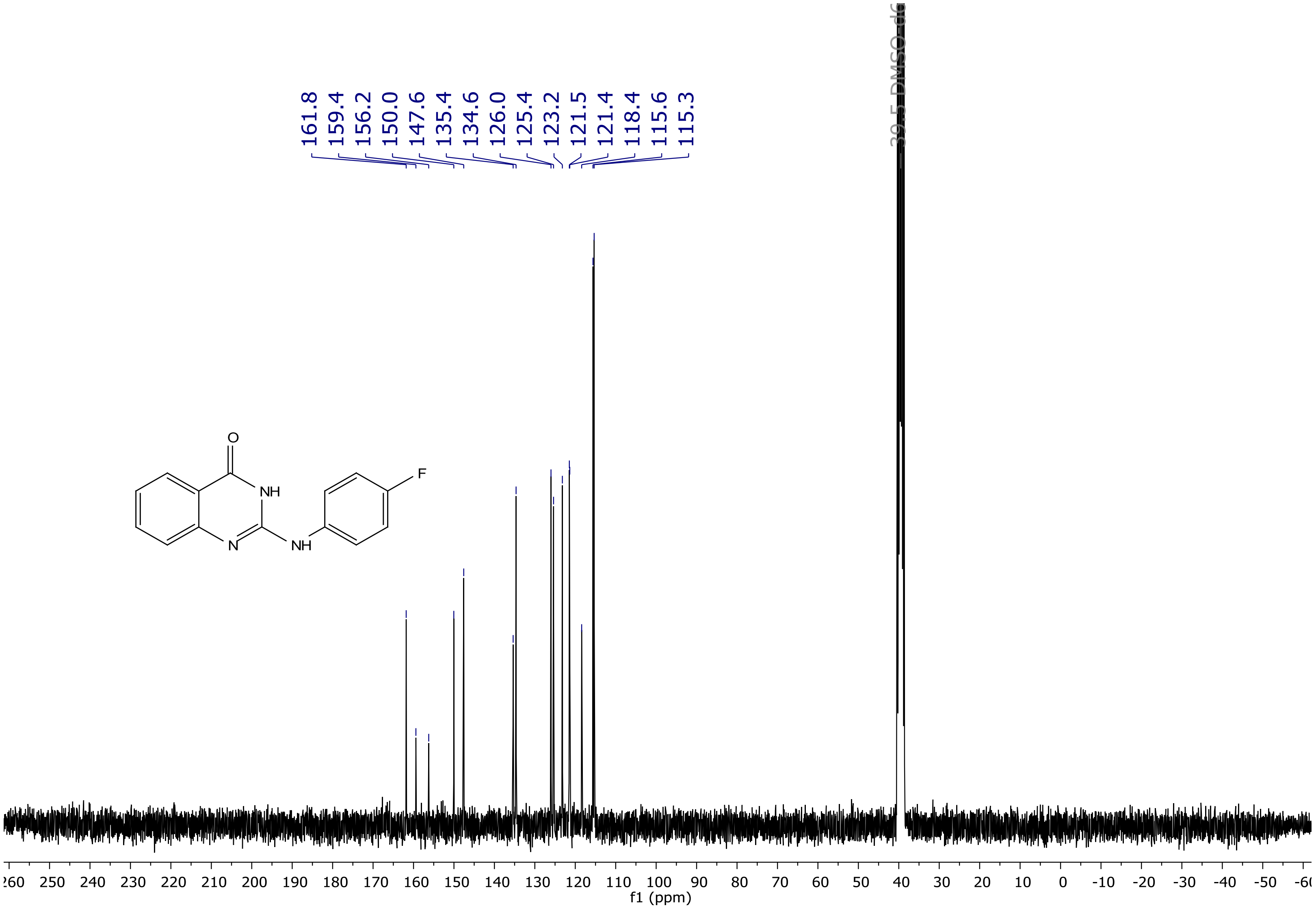

^{1}H NMR (300 MHz, DMSO-d_6) of 2-((2,3-dihydrobenzo[*b*][1,4]dioxin-6-yl)amino)quinazolin-4(3*H*)-one (**10c**)

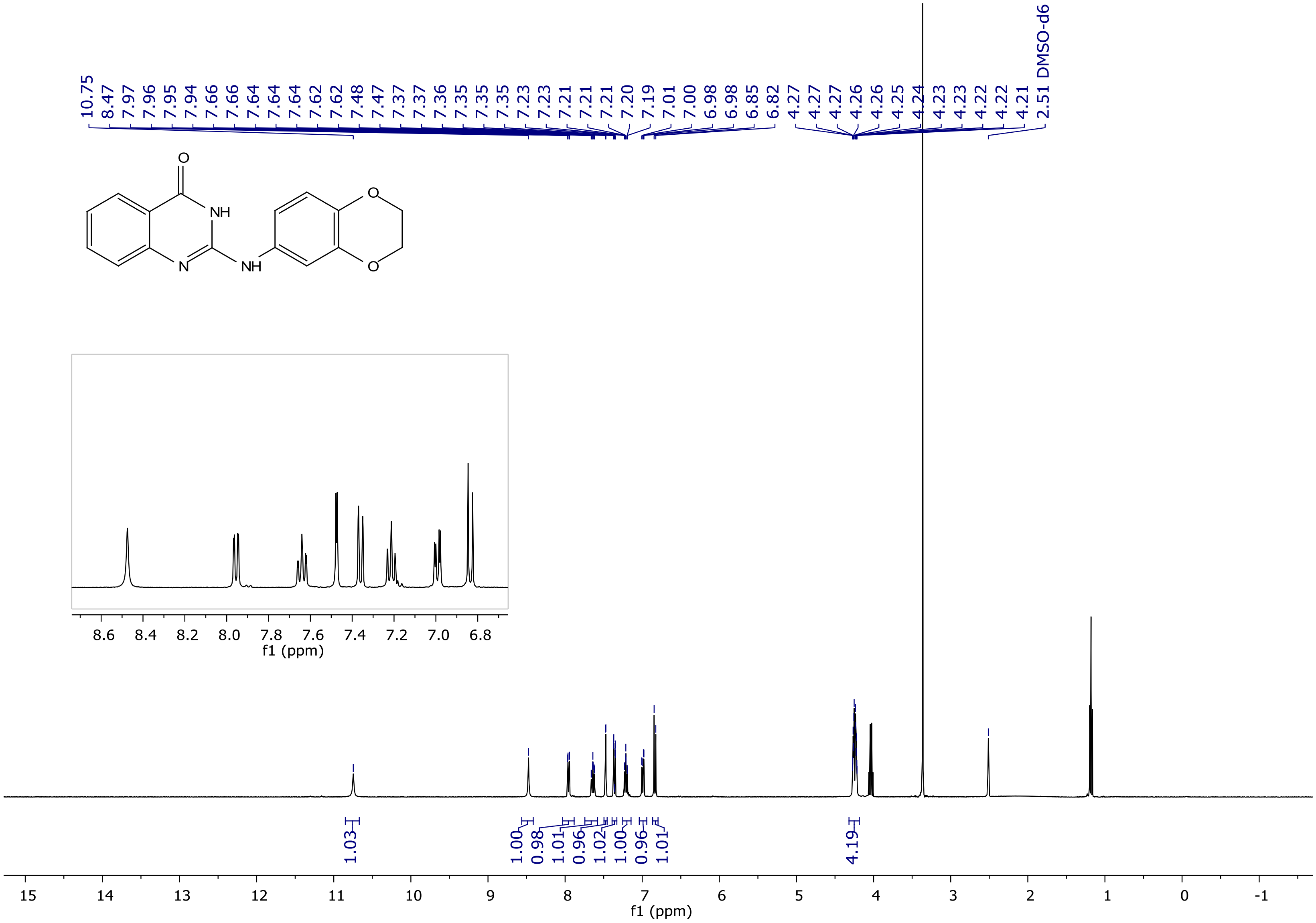

^{13}C NMR (75 MHz, DMSO-d_6) of 2-((2,3-dihydrobenzo[*b*][1,4]dioxin-6-yl)amino)quinazolin-4(3*H*)-one (**10c**)

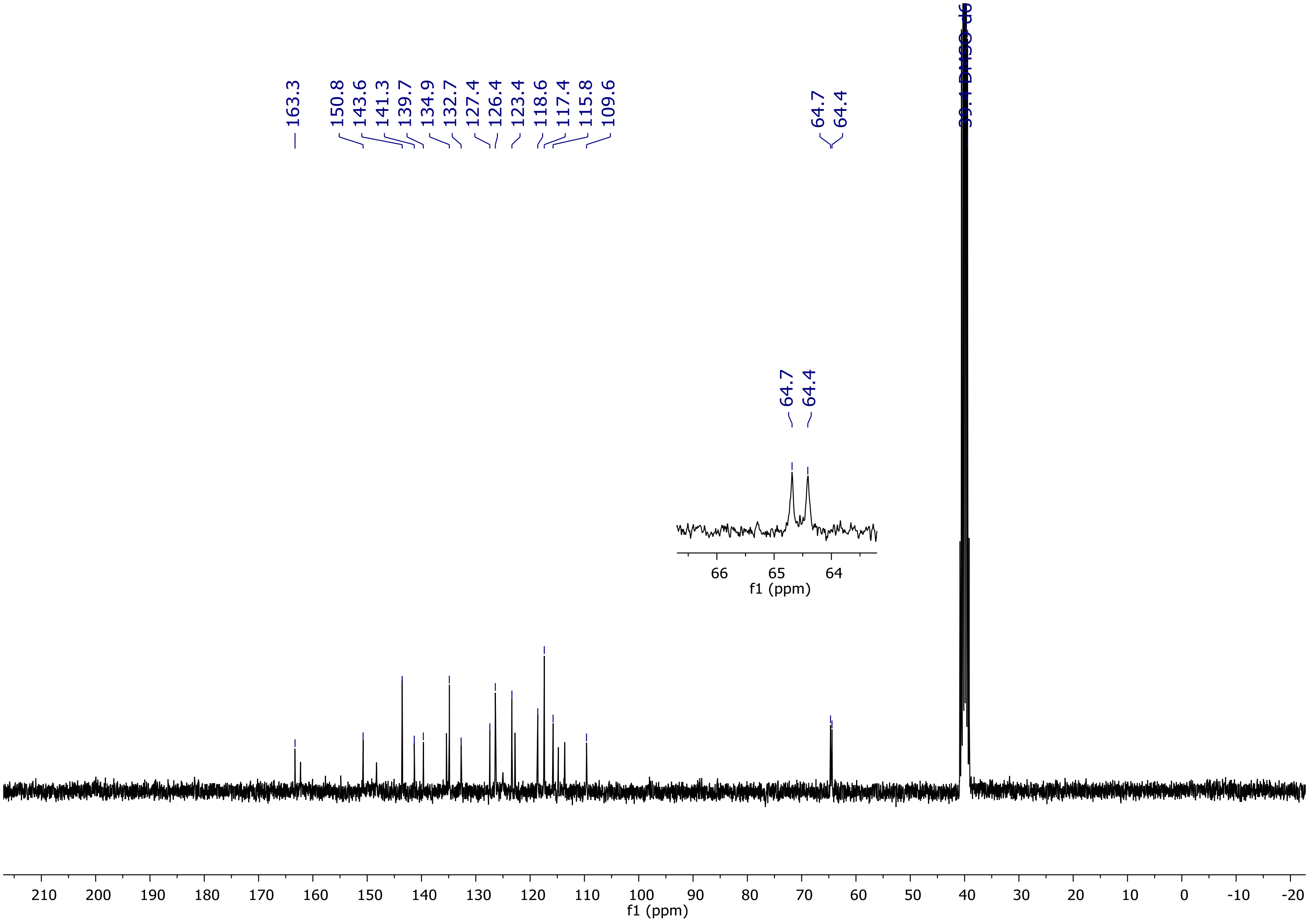

FTIR of 2-((2,3-dihydrobenzo[*b*][1,4]dioxin-6-yl)amino)quinazolin-4(3*H*)-one (**10c**)

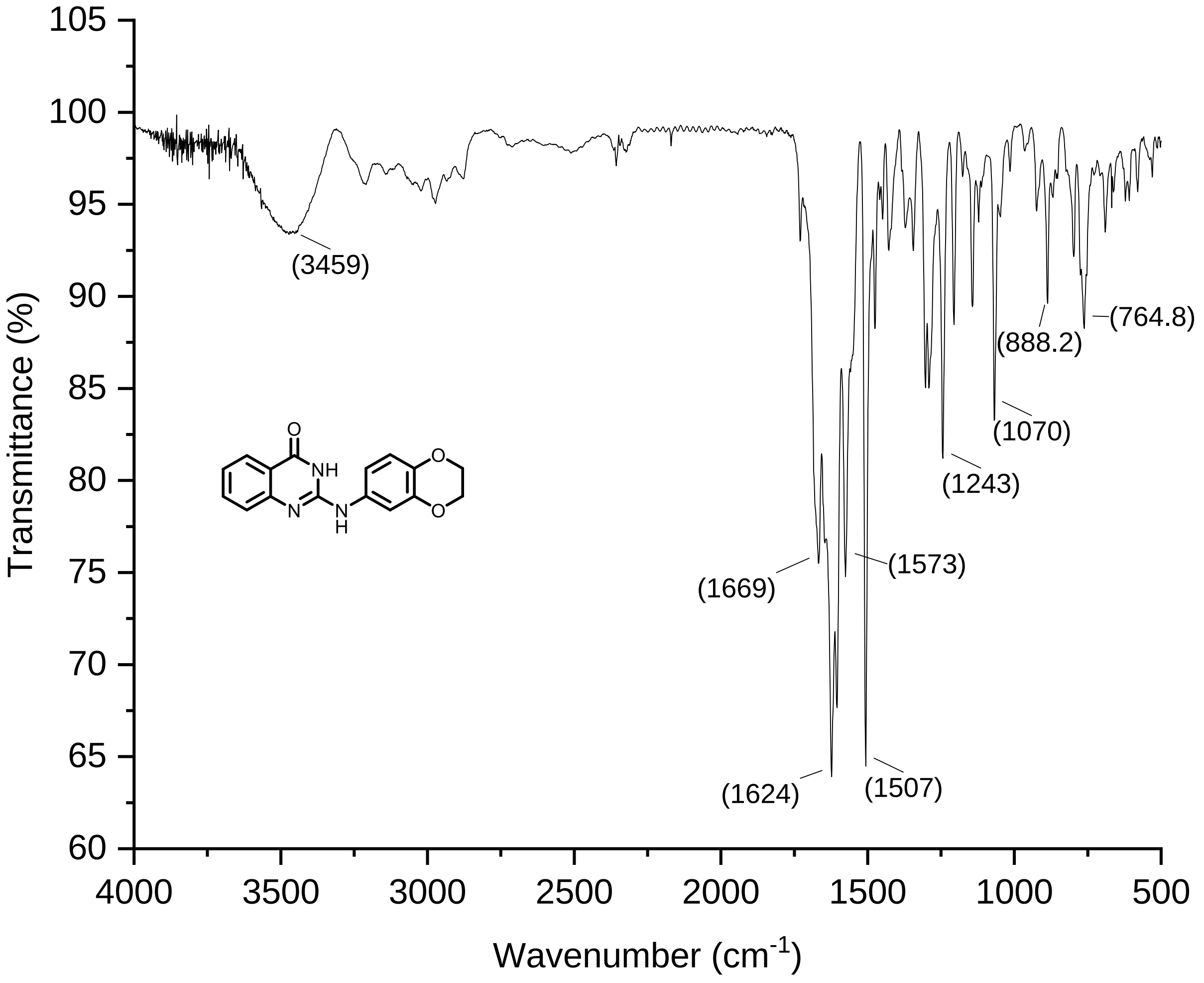

^{1}H NMR (400MHz, DMSO-d6) of 2-([1,1'-biphenyl]-4-ylamino)quinazolin-4(3H)-one (**10d**)

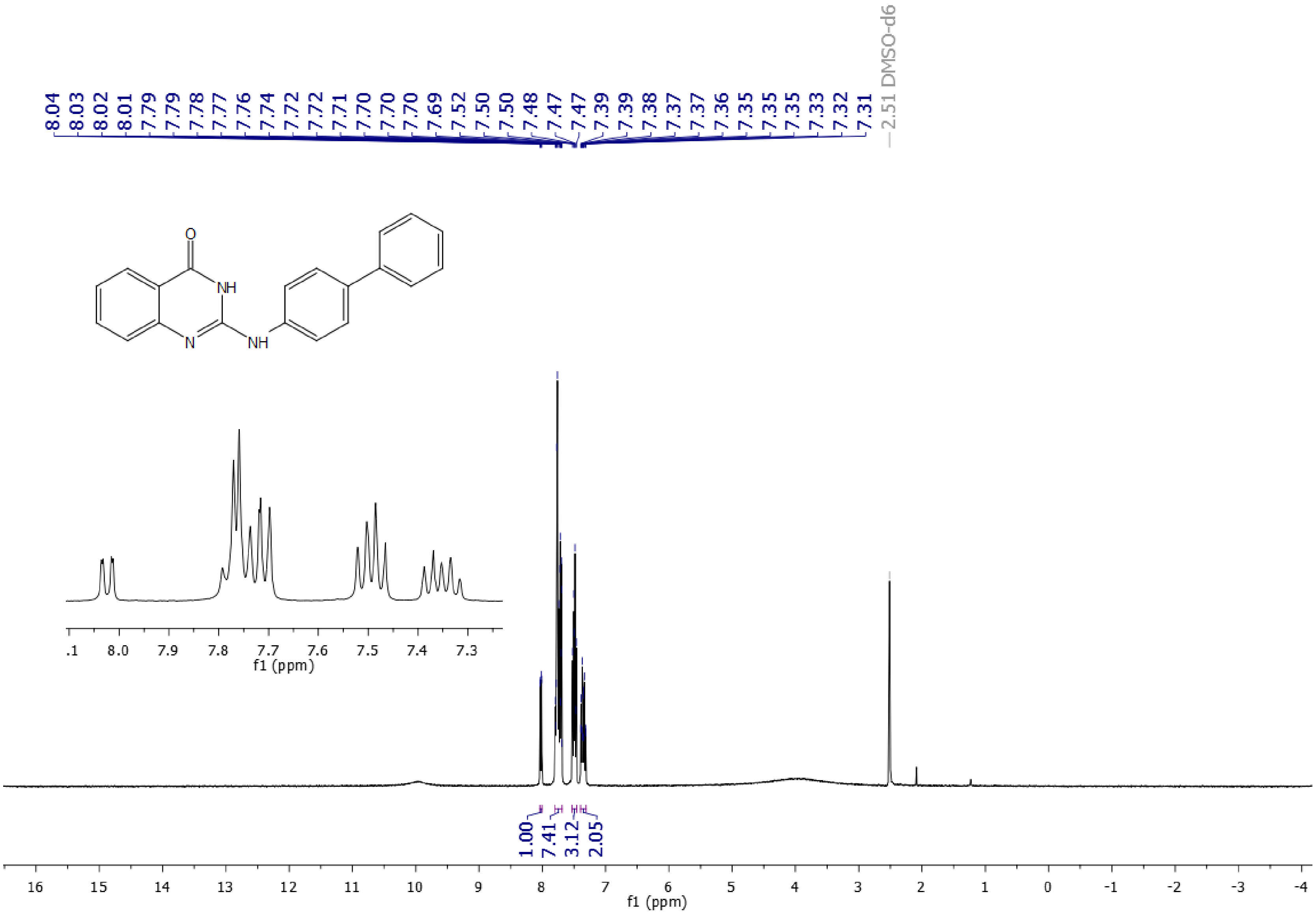

^{13}C NMR (101MHz, DMSO-d6) of 2-([1,1'-biphenyl]-4-ylamino)quinazolin-4(3H)-one (**10d**)

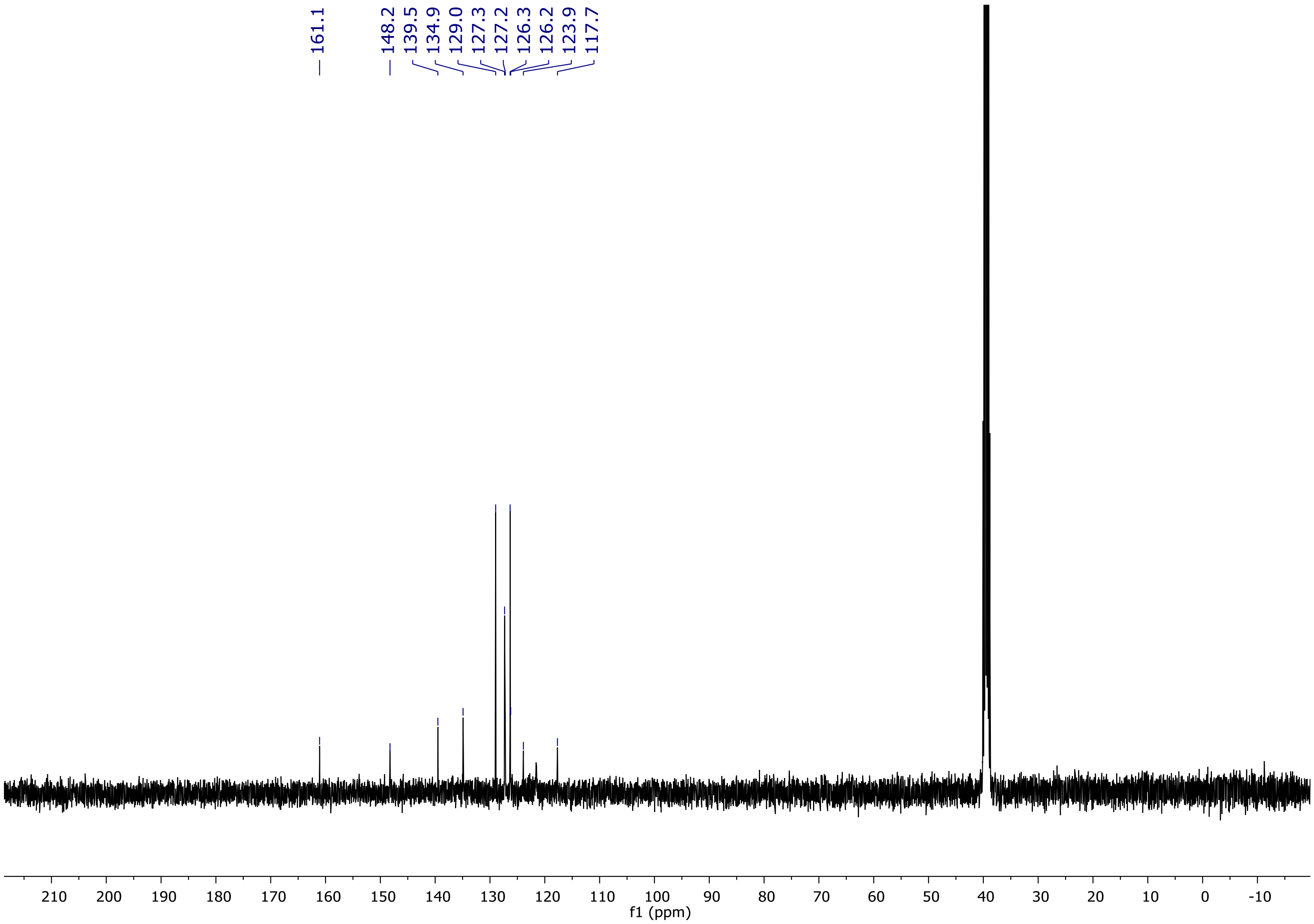

^{1}H NMR (400MHz, DMSO-d6) of 2-([1,1'-biphenyl]-3-ylamino)quinazolin-4(3H)-one (**10e**)

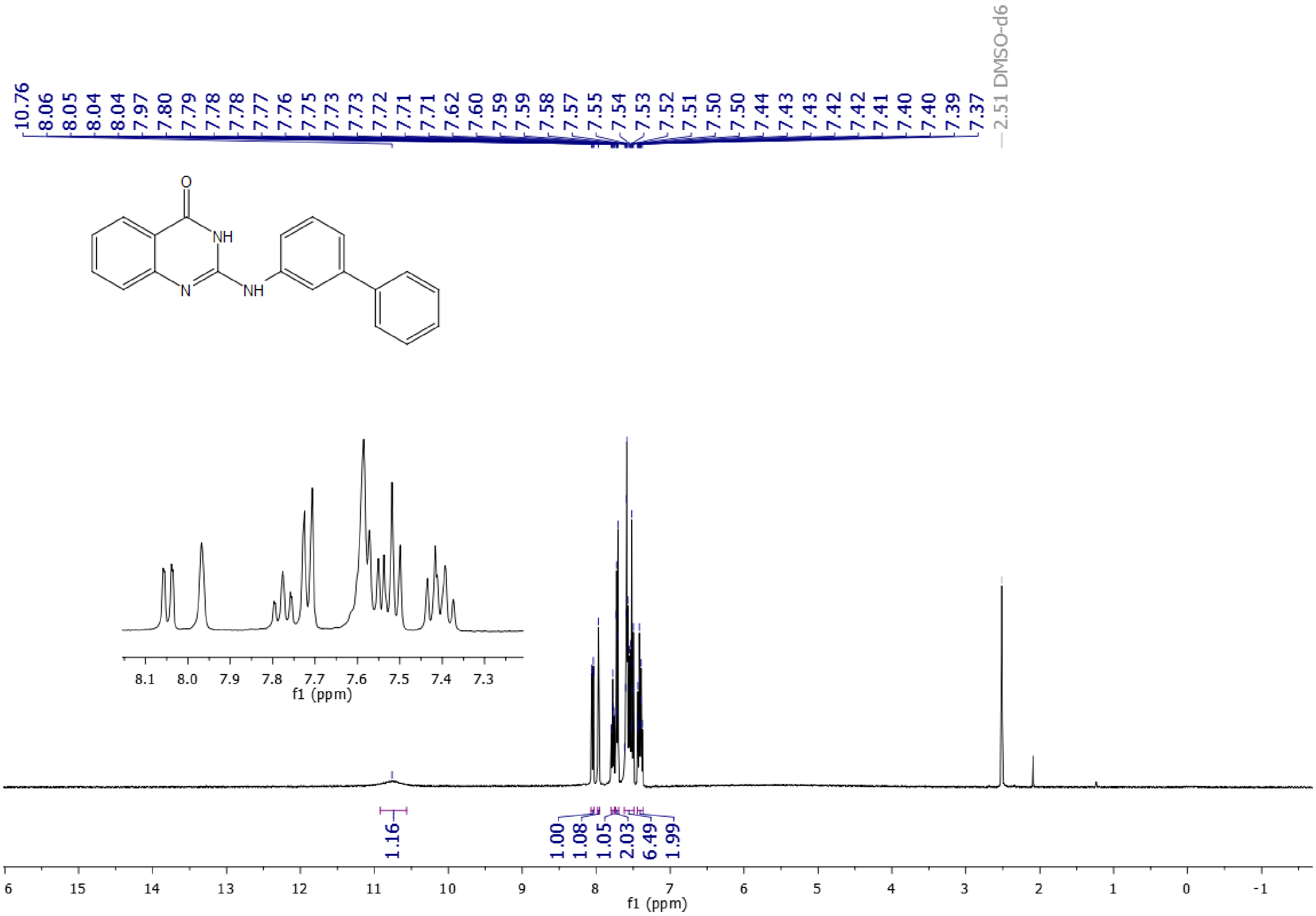

^{13}C NMR (101MHz, DMSO-d6) of 2-([1,1'-biphenyl]-3-ylamino)quinazolin-4(3H)-one (**10e**)

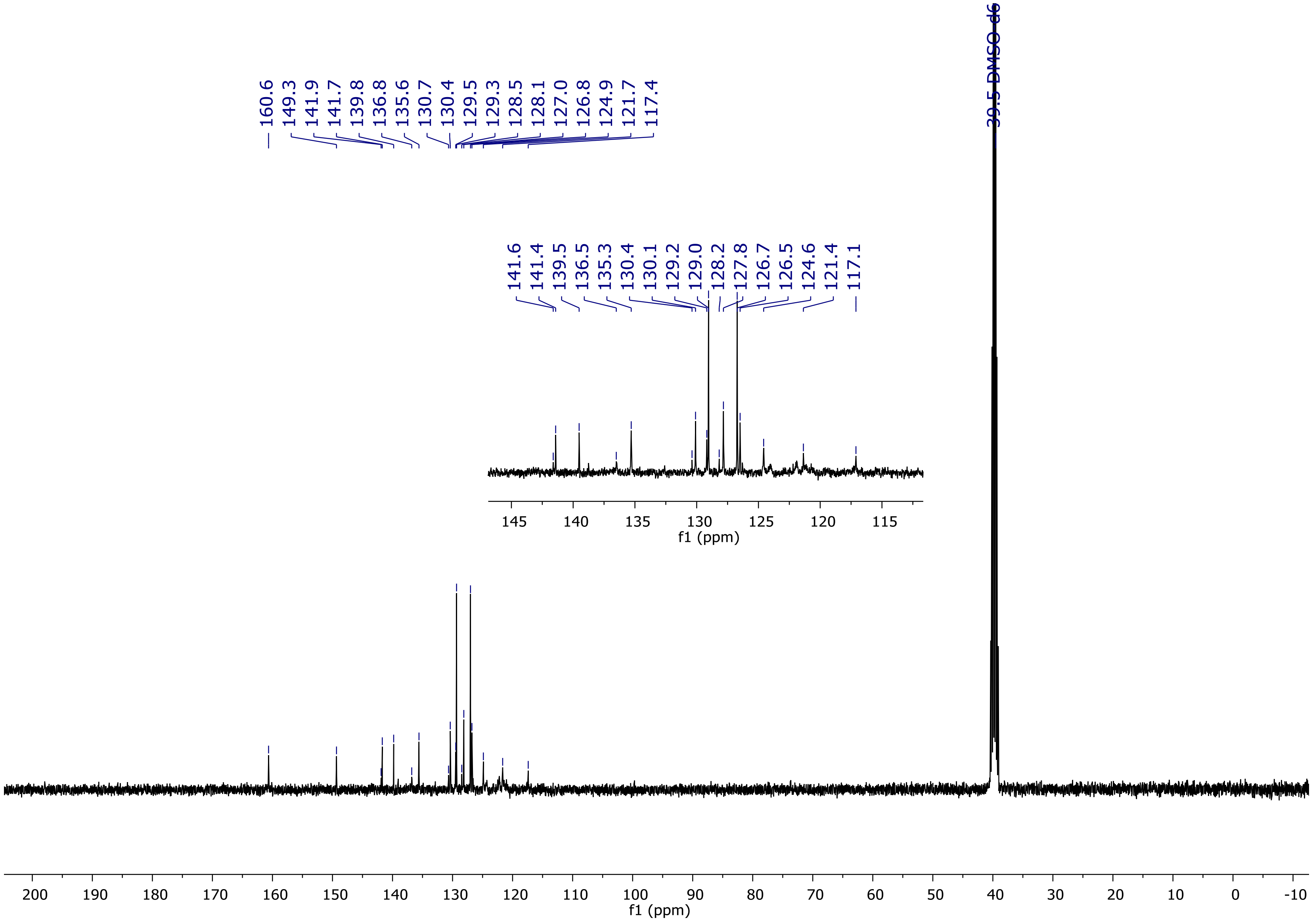

^{1}H NMR (300MHz, DMSO-d6) of 2-(4-(3-(trifluoromethyl)phenyl)piperazin-1-yl)quinazolin-4(3H)-one **(10f)**

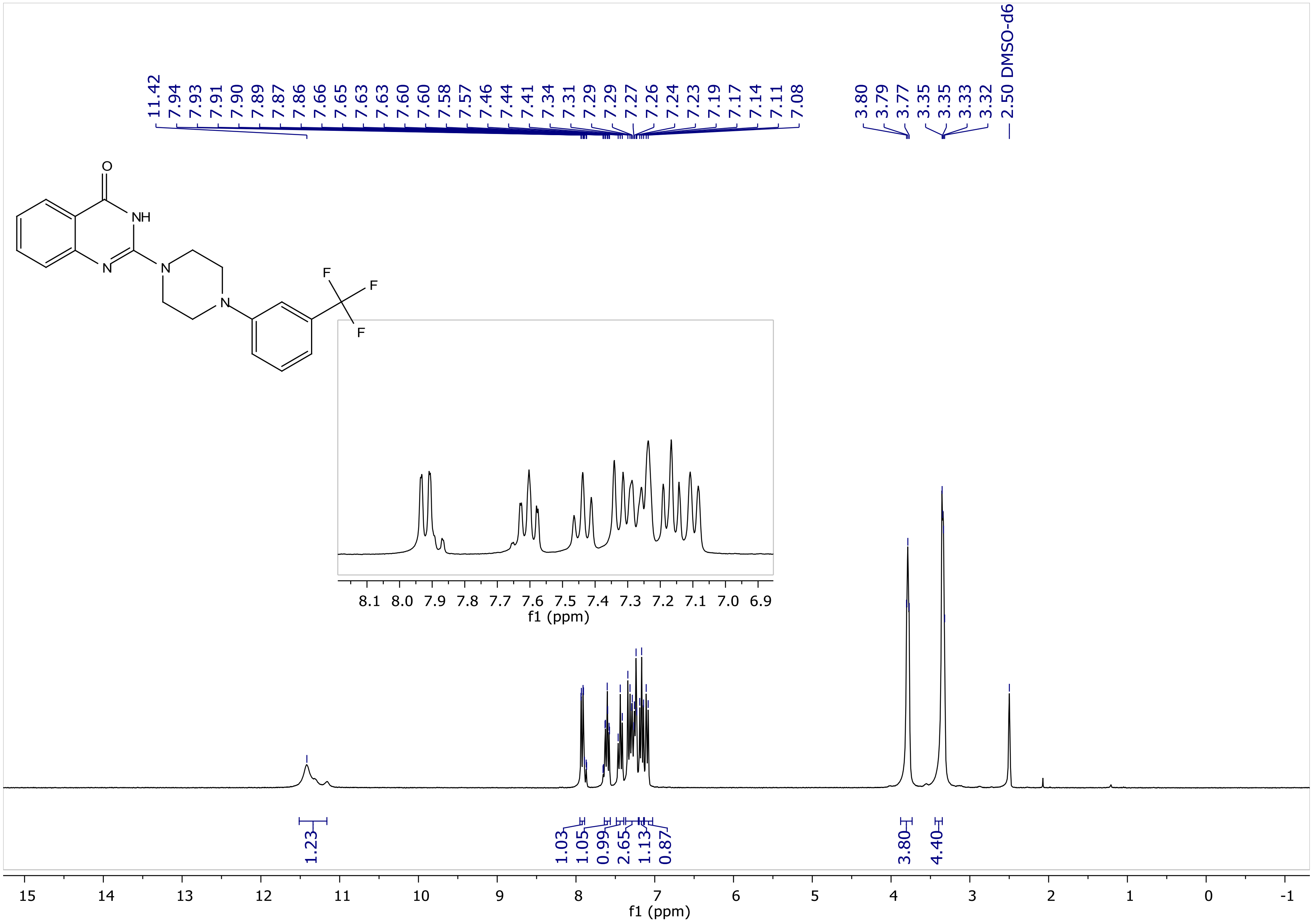

^{13}C (75MHz, DMSO-d6) of 2-(4-(3-(trifluoromethyl)phenyl)piperazin-1-yl)quinazolin-4(3H)-one **(10f)**

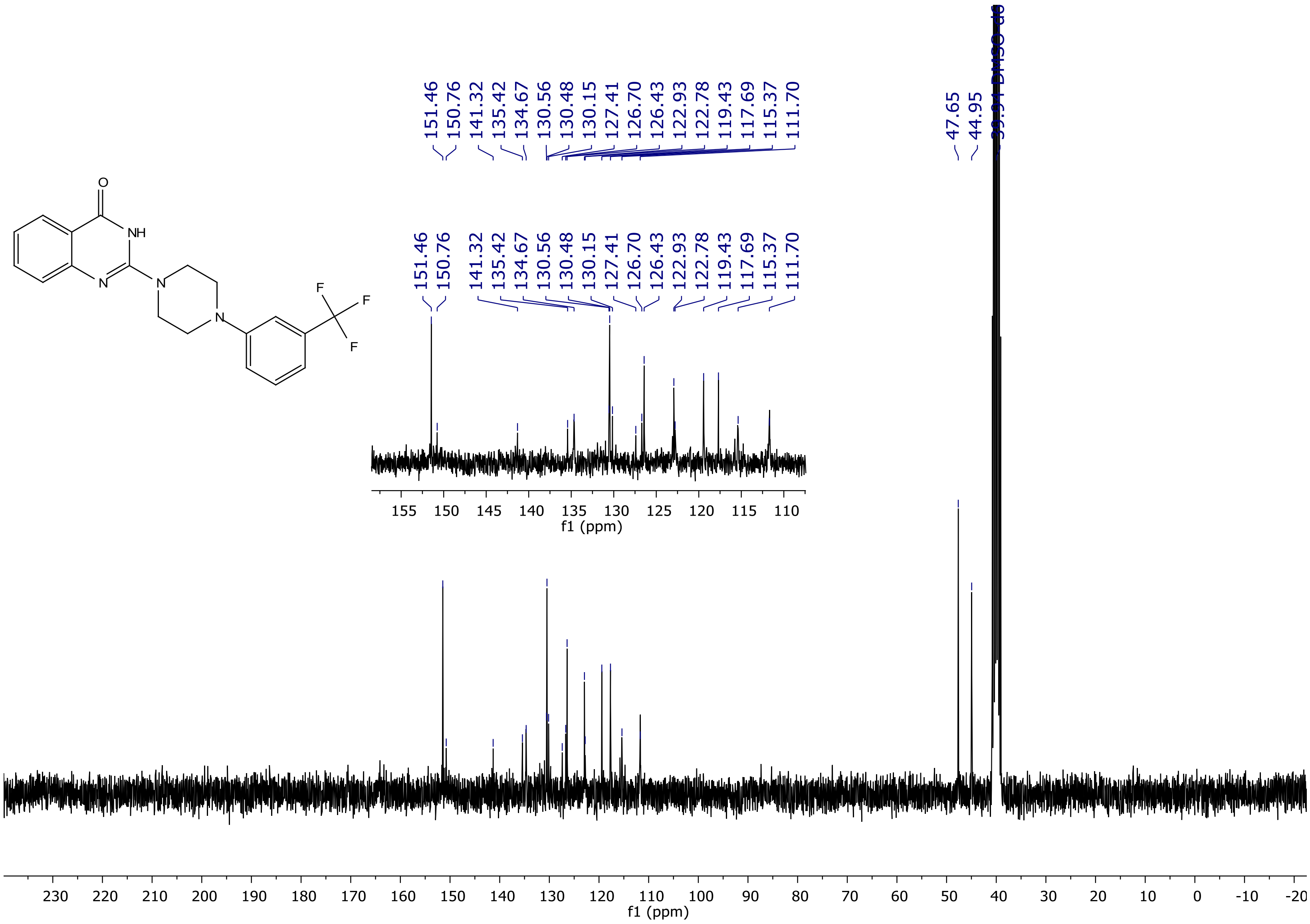

FTIR of 2-(4-(3-(trifluoromethyl)phenyl)piperazin-1-yl)quinazolin-4(3H)-one **(10f)**

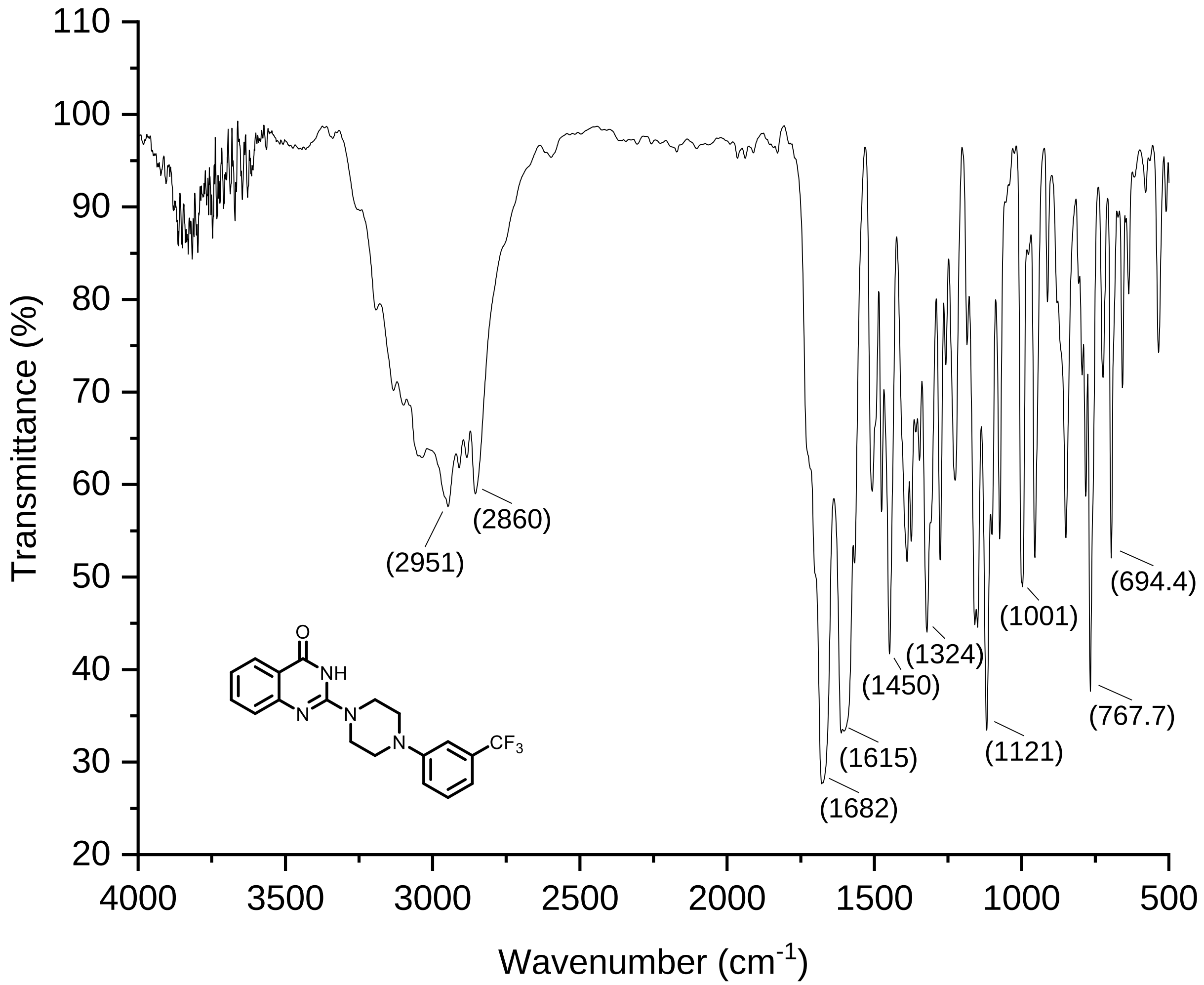

^{1}H (300MHz, DMSO-d6) of 2-(4-phenylpiperazin-1-yl)quinazolin-4(3H)-one (**10g**)

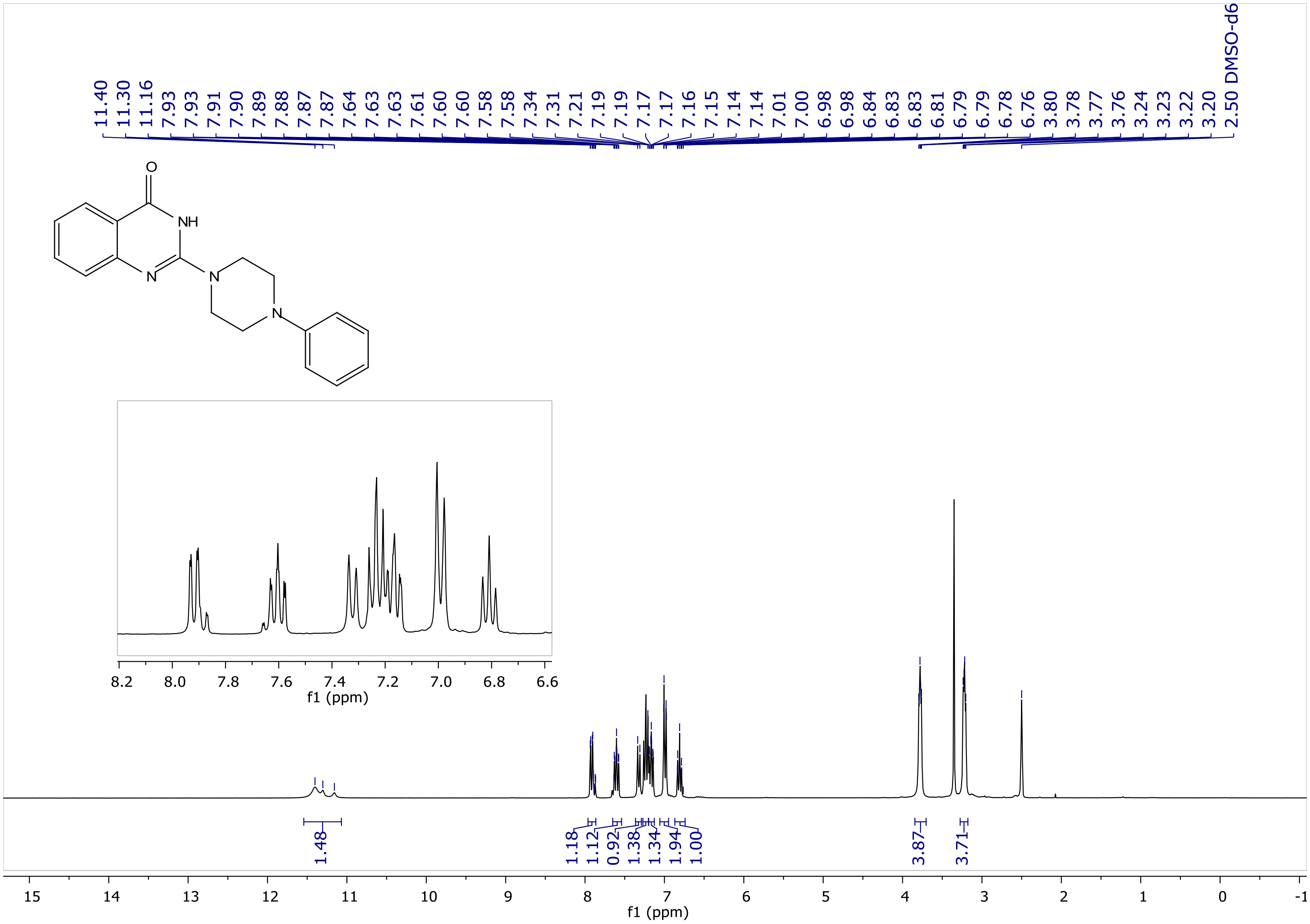

^{13}C (75MHz, DMSO-d6) of 2-(4-phenylpiperazin-1-yl)quinazolin-4(3H)-one (**10g**)

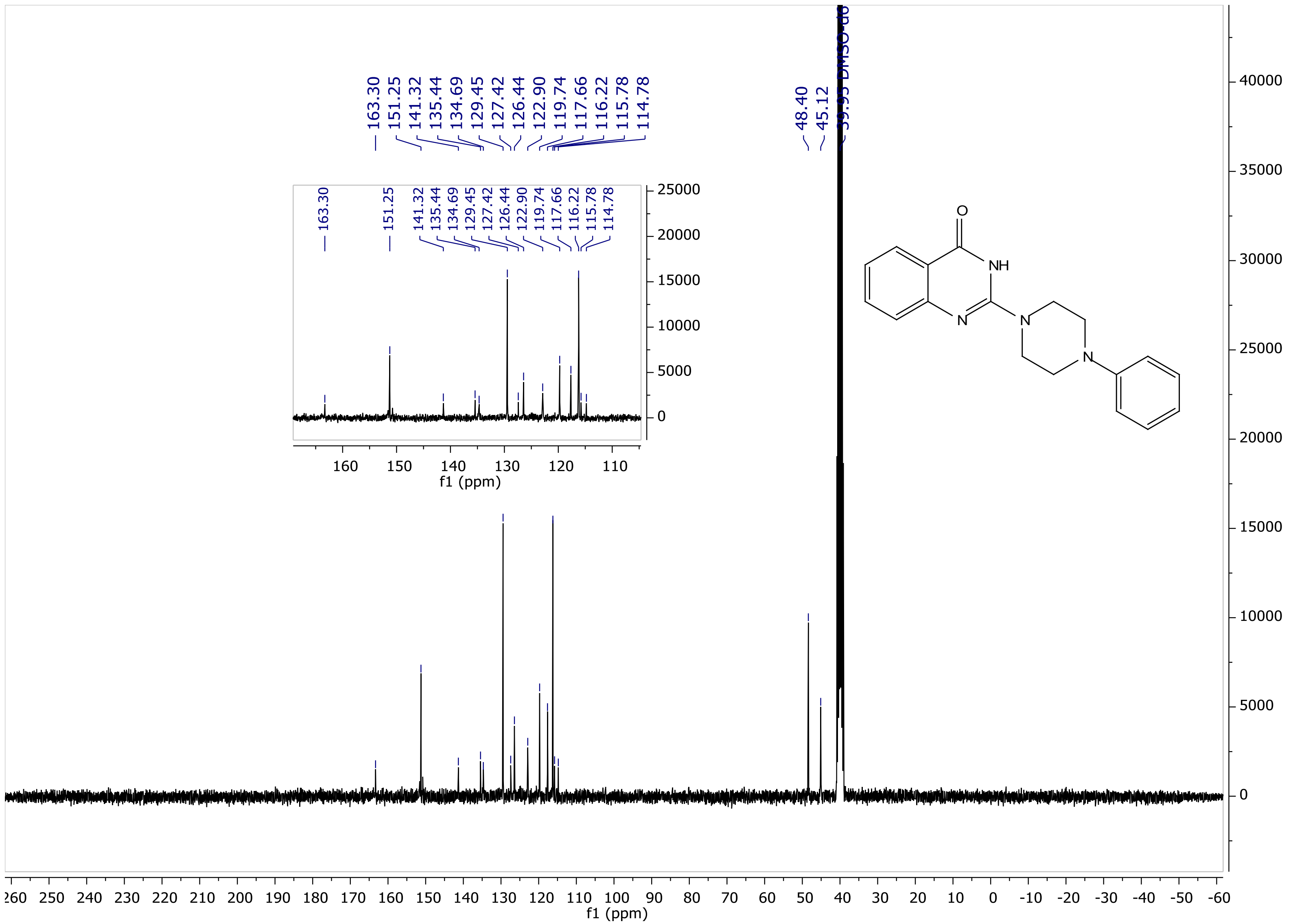

^{1}H NMR (300MHz, DMSO-d6) of 2-(benzylamino)quinazolin-4(3H)-one (**10h**)

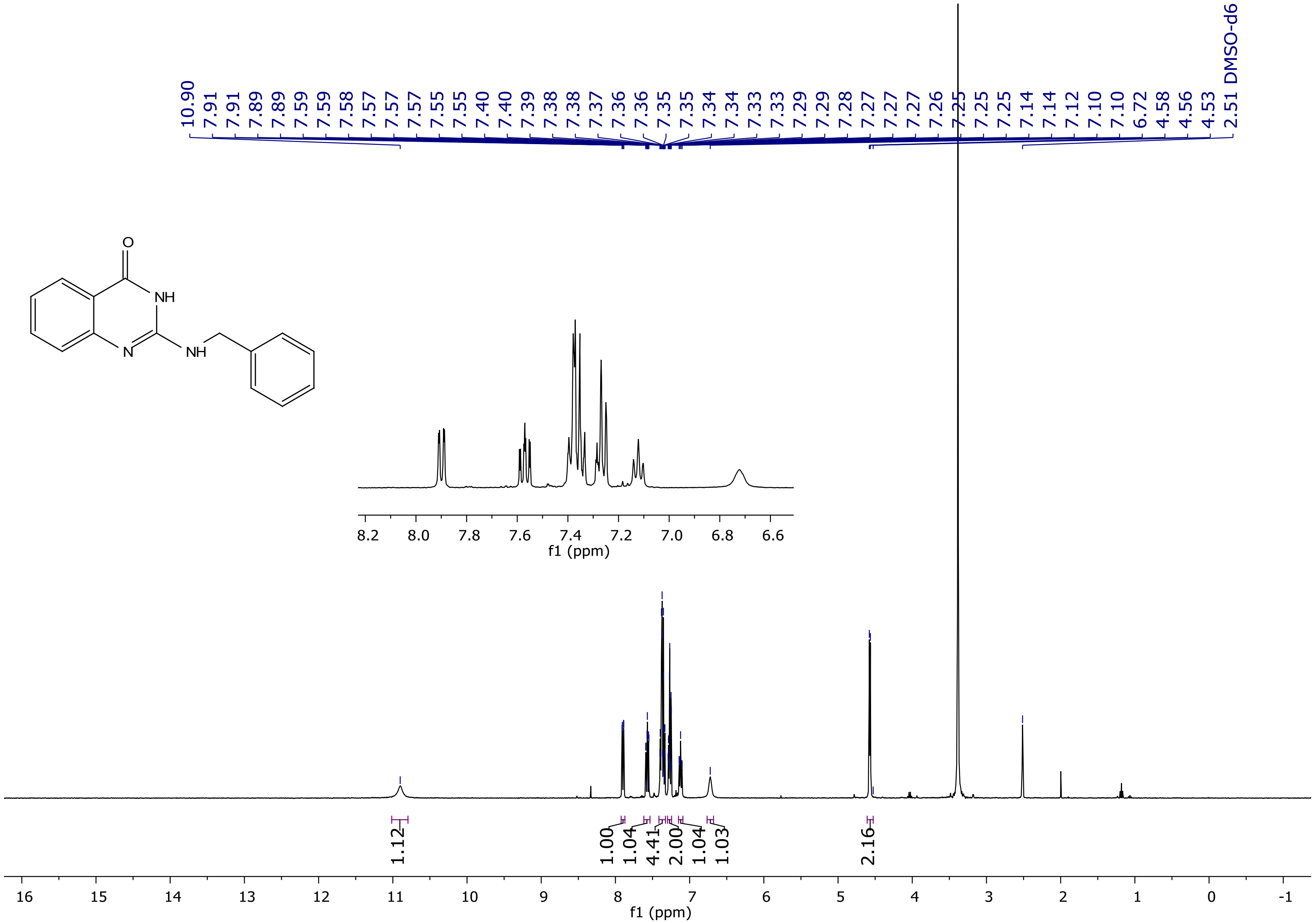

^{13}C NMR (101MHz, DMSO-d6) of 2-(benzylamino)quinazolin-4(3H)-one (**10h**)

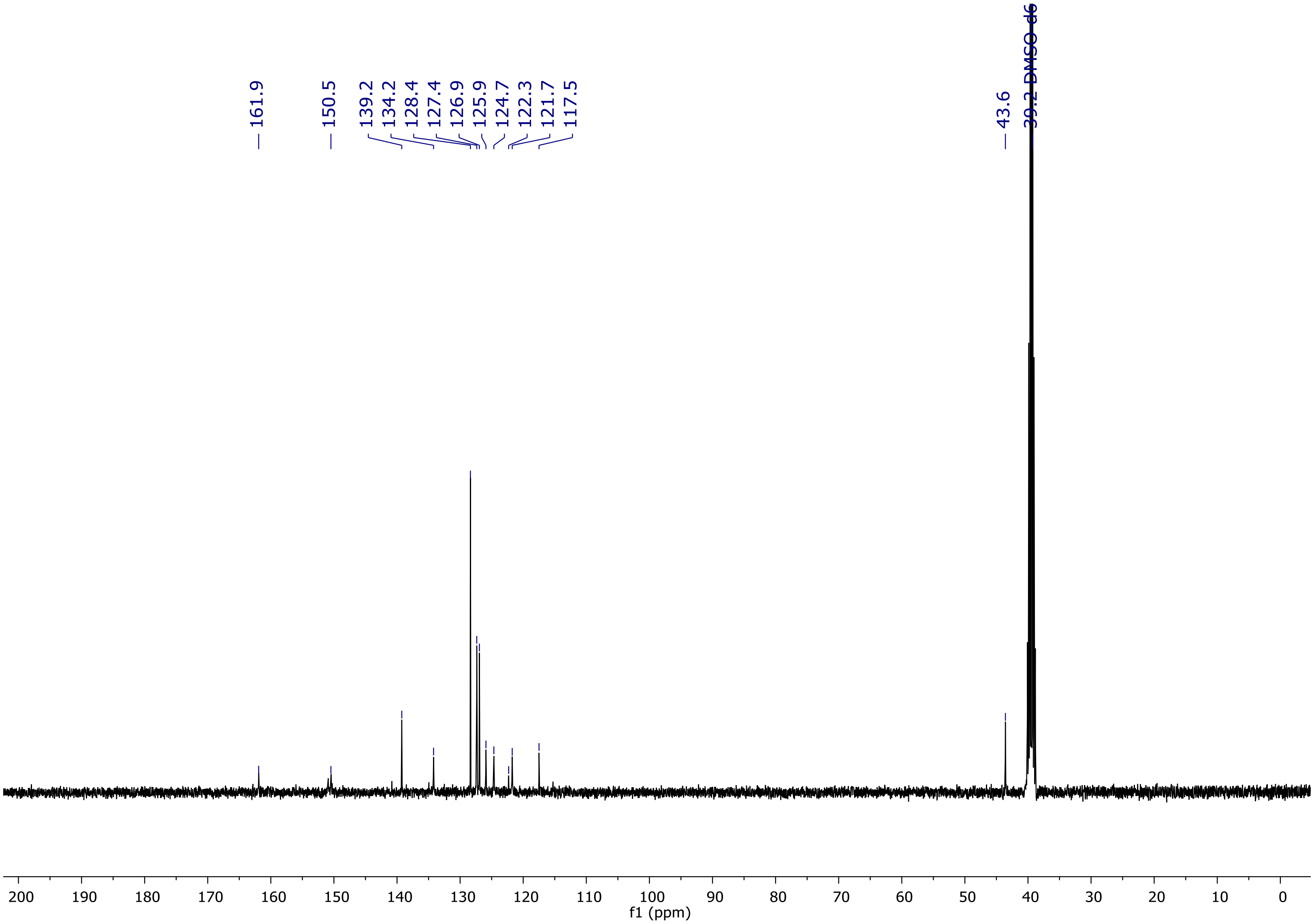

^{1}H NMR (400MHz, DMSO-d6) of 2-(phenethylamino)quinazolin-4(3H)-one (**10i**)

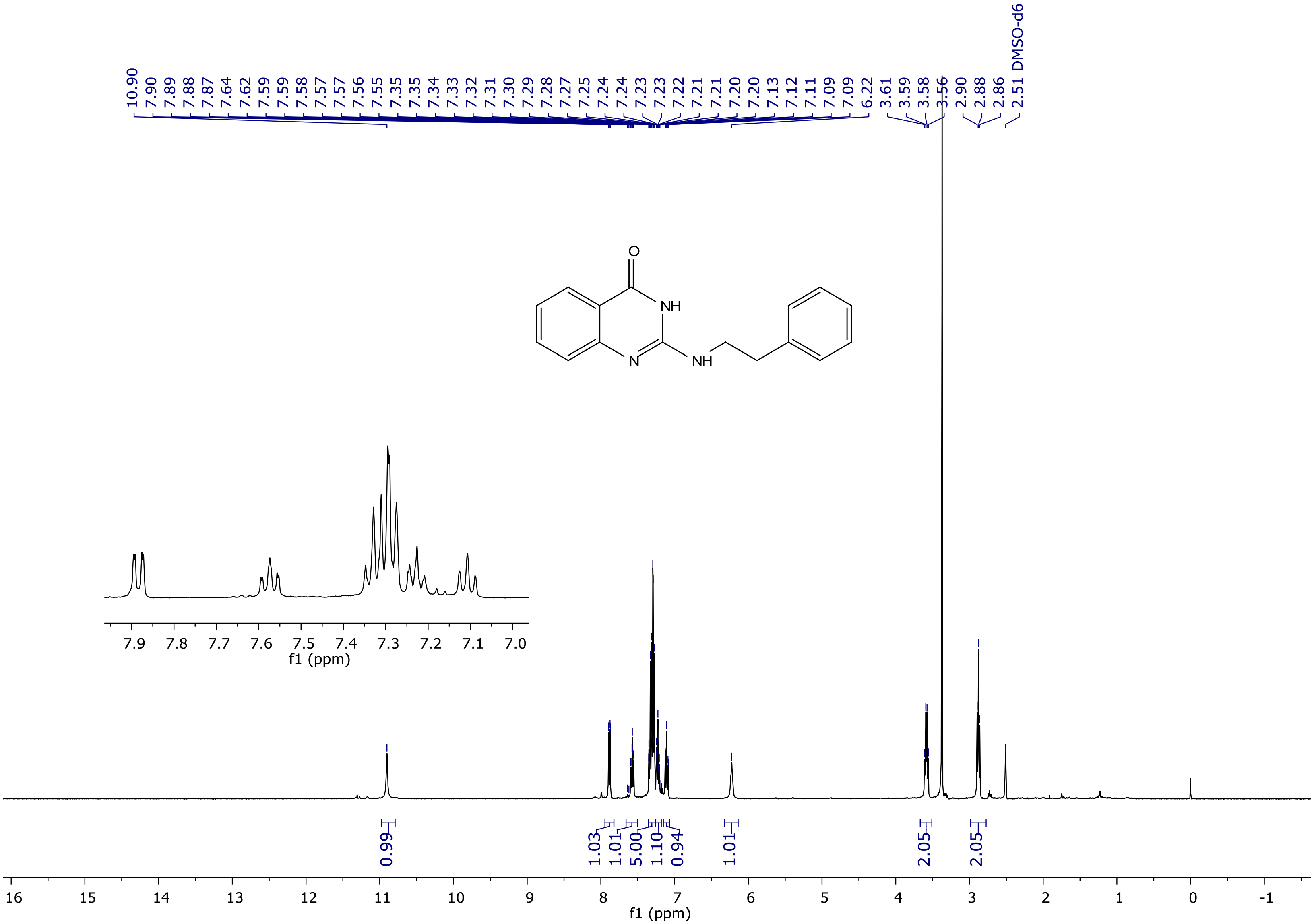

^{13}C NMR (101MHz, DMSO-d6) of 2-(phenethylamino)quinazolin-4(3H)-one (**10i**)

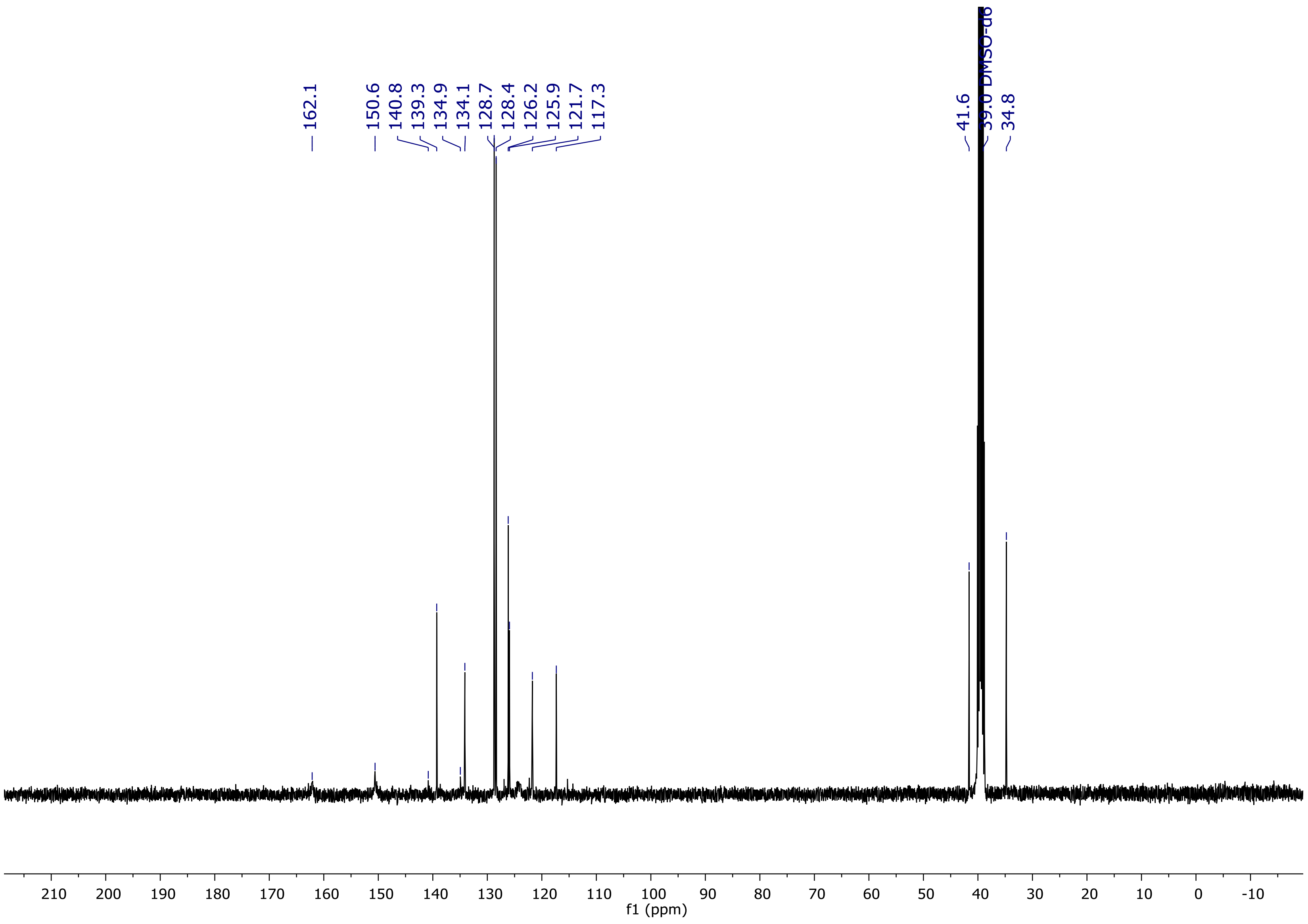

^{1}H (300MHz, DMSO-d6) of 2-((4-fluorophenethyl)amino)quinazolin-4(3H)-one **(10j)**

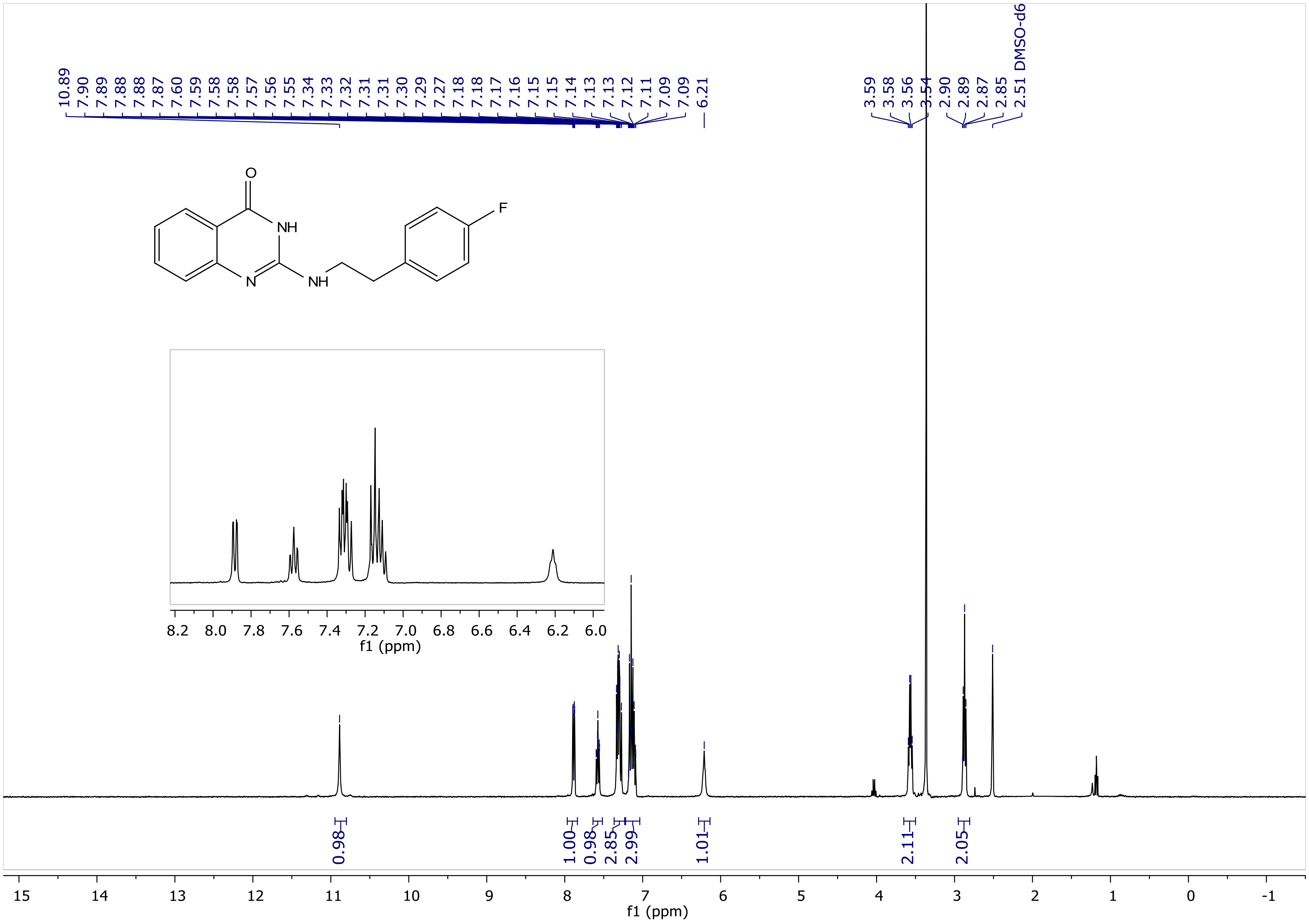

^{13}C NMR (75MHz, DMSO-d6) of 2-((4-fluorophenethyl)amino)quinazolin-4(3H)-one **(10j)**

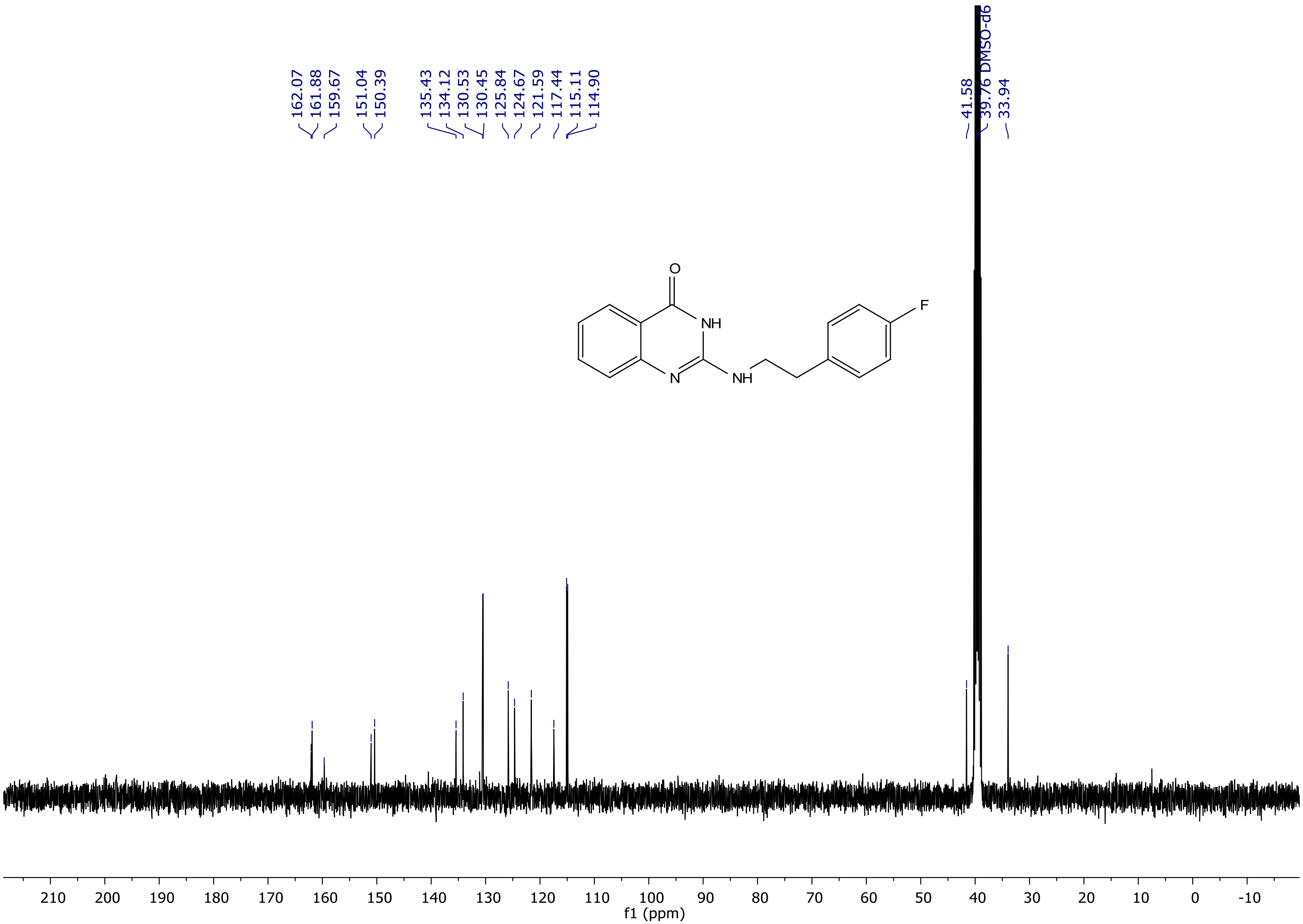

FTIR spectra of 2-((4-fluorophenethyl)amino)quinazolin-4(3H)-one **(10j)**

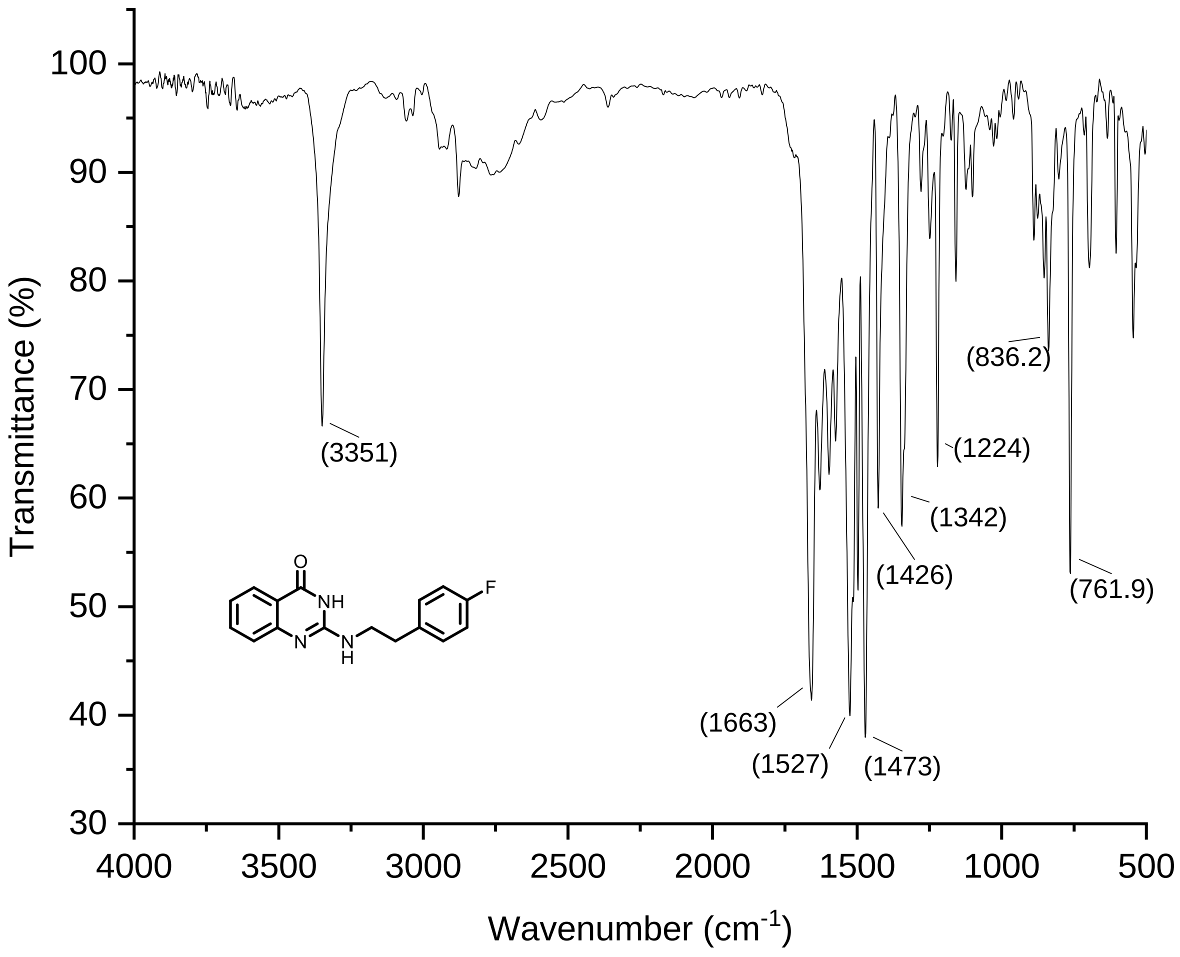

^{1}H NMR (400MHz, DMSO-d6) of 2-((3-phenylpropyl)amino)quinazolin-4(3H)-one (**10k**)

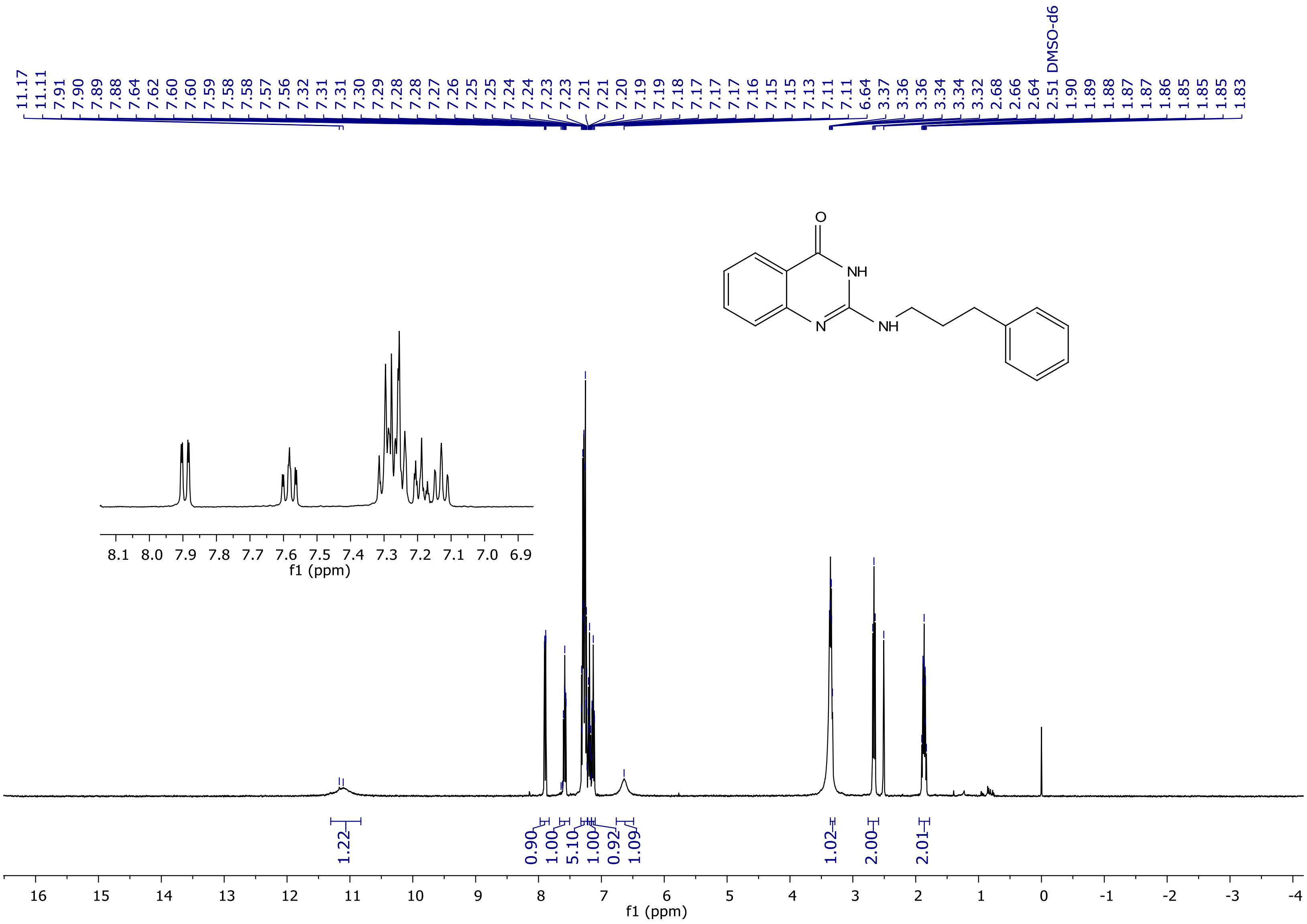

^{13}C NMR (100MHz, DMSO-d6) of 2-((3-phenylpropyl)amino)quinazolin-4(3H)-one (**10k**)

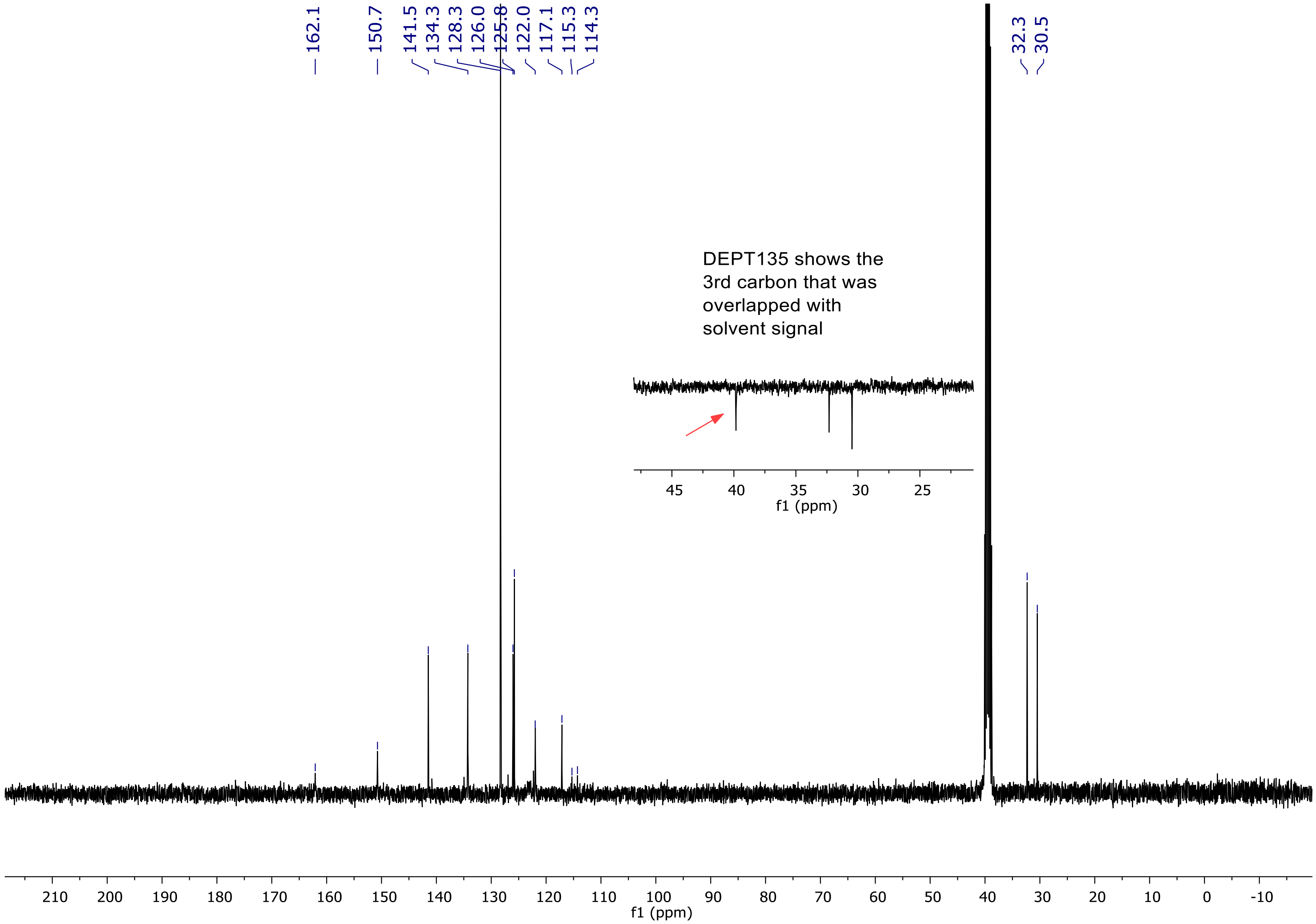

^{1}H NMR (400MHz, DMSO-d6) of 2-((4-phenylbutyl)amino)quinazolin-4(3H)-one (**10l**)

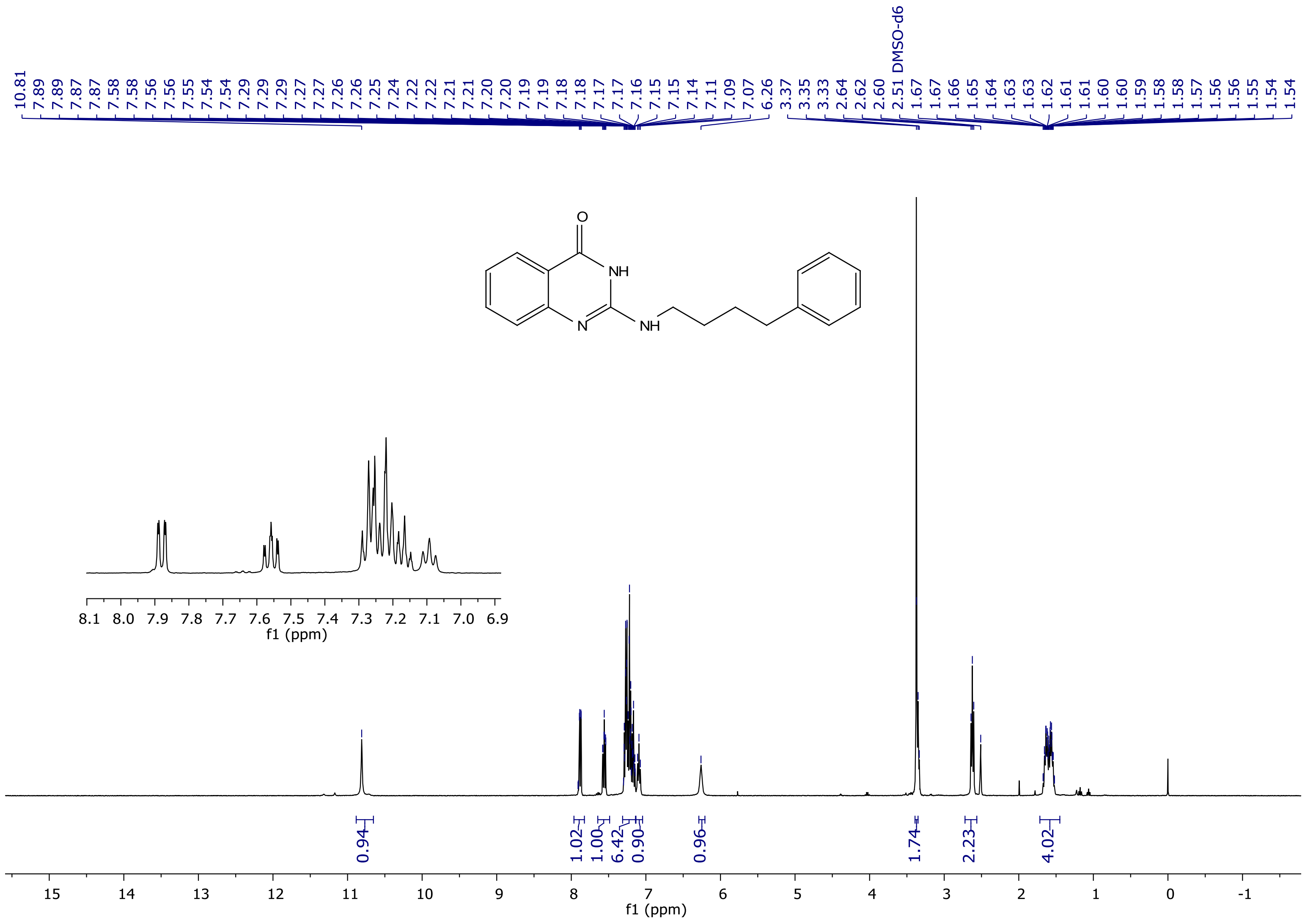

^{13}C NMR (101MHz, DMSO-d6) of 2-((4-phenylbutyl)amino)quinazolin-4(3H)-one **(10l)**

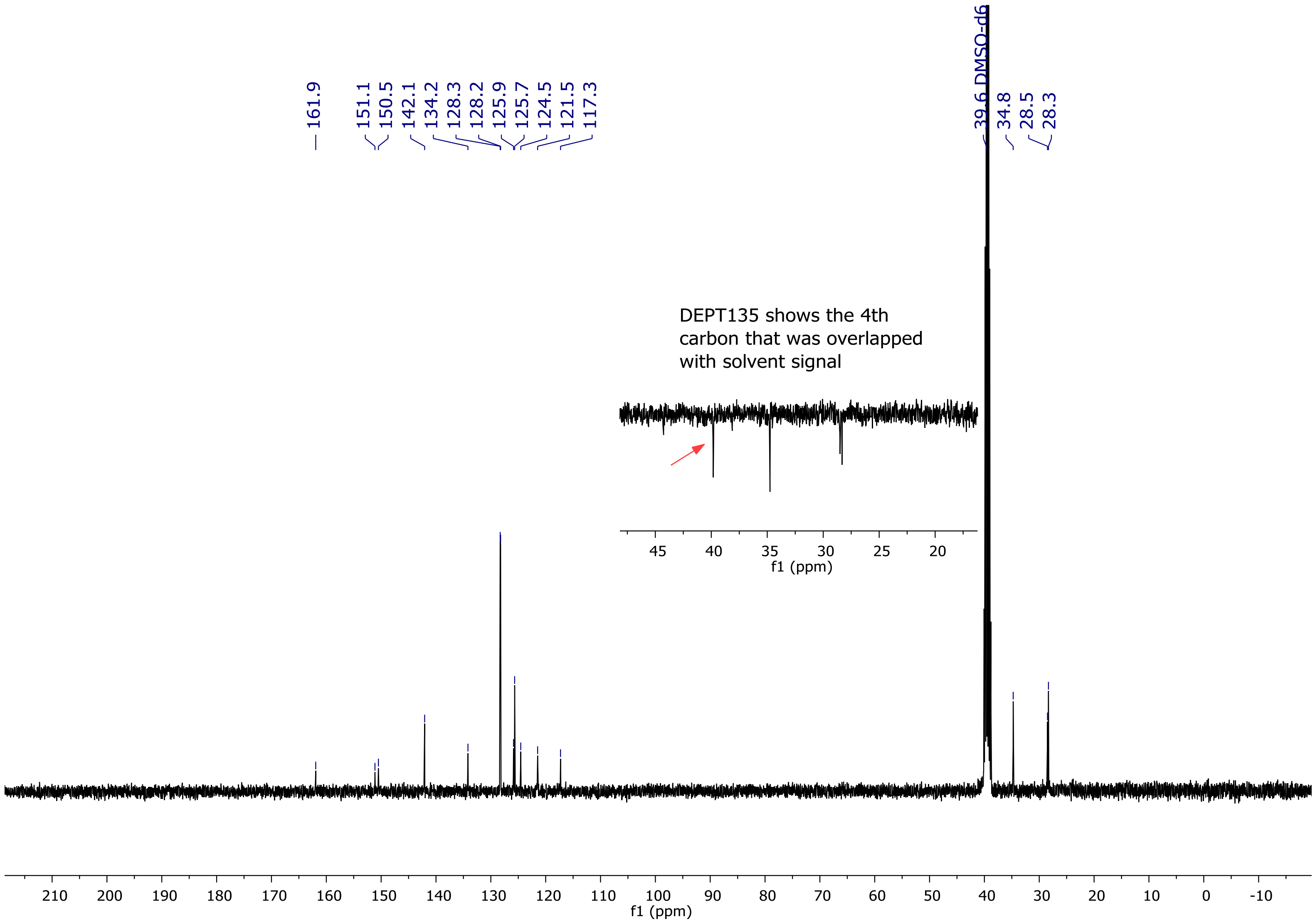

FTIR of 2-((4-phenylbutyl)amino)quinazolin-4(3H)-one (**10l**)

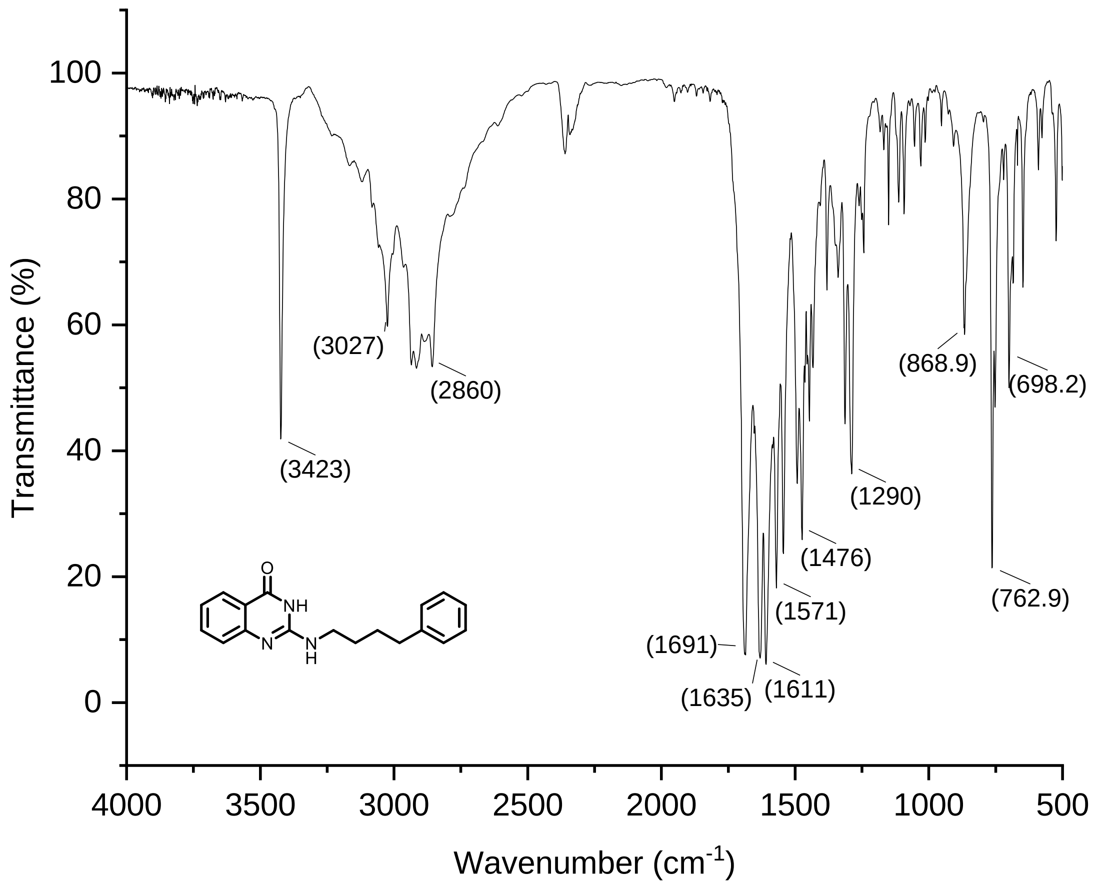

Supplement: Supplementary file 1 [file ml5c00237_si_001.pdf]
